# Affinity‐Directed Site‐Specific Protein Labeling and Its Application to Antibody‐Drug Conjugates

**DOI:** 10.1002/advs.202306401

**Published:** 2023-11-30

**Authors:** Sooin Kim, Sanggil Kim, Sangji Kim, Namkyoung Kim, Sang Won Lee, Hanbin Yi, Seungeun Lee, Taebo Sim, Yongseok Kwon, Hyun Soo Lee

**Affiliations:** ^1^ Department of Chemistry Sogang University 35 Baekbeom‐ro, Mapo‐gu Seoul 04107 Republic of Korea; ^2^ New Drug Development Center Osong Medical Innovation Foundation 123 Osongsaengmyeong‐ro, Heungdeok‐gu Cheongju Chungbuk 28160 Republic of Korea; ^3^ School of Pharmacy Sungkyunkwan University 2066 Seobu‐ro, Jangan‐gu Suwon 16419 Republic of Korea; ^4^ Department of Biomedical Sciences Graduate School of Medical Science Brain Korea 21 Project Yonsei University College of Medicine 50 Yonsei‐ro, Seodaemun‐gu Seoul 03722 Republic of Korea

**Keywords:** antibody‐drug conjugates, bioorthogonal chemistry, genetic code expansion, native protein labeling, small helical binding protein

## Abstract

Chemically modified proteins have diverse applications; however, conventional chemo‐selective methods often yield heterogeneously labeled products. To address this limitation, site‐specific protein labeling holds significant potential, driving extensive research in this area. Nevertheless, site‐specific modification of native proteins remains challenging owing to the complexity of their functional groups. Therefore, a method for site‐selective labeling of intact proteins is aimed to design. In this study, a novel approach to traceless affinity‐directed intact protein labeling is established, which leverages small binding proteins and genetic code expansion technology. By applying this method, a site‐specific antibody labeling with a drug, which leads to the production of highly effective antibody‐drug conjugates specifically targeting breast cancer cell lines is achieved. This approach enables traceless conjugation of intact target proteins, which is a critical advantage in pharmaceutical applications. Furthermore, small helical binding proteins can be easily engineered for various target proteins, thereby expanding their potential applications in diverse fields. This innovative approach represents a significant advancement in site‐specific modification of native proteins, including antibodies. It also bears immense potential for facilitating the development of therapeutic agents for various diseases.

## Graphical abstract

A novel method for precise, affinity‐driven site‐specific protein labeling, leveraging small binding proteins and genetic code expansion technology is presented. This innovative approach enables the conjugation of intact target proteins, with a specific emphasis on its utility in advancing the field of antibody‐drug conjugates. The inherent versatility of these binding proteins unveils promising avenues for a wide spectrum of applications.

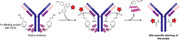

## 1 Introduction

Proteins are indispensable biomolecules that play fundamental roles in nature. Despite their versatility in performing numerous functions with only 20 amino acids as building blocks, more complex functions often require additional posttranslational modifications and cofactors. Although naturally occurring proteins may be evolutionarily optimized for their cellular functions, the continuous demand for proteins with diverse functionalities in biochemical and pharmaceutical applications persists. For example, the conjugation of antibodies with cytotoxic drugs plays a crucial role in the development of antibody‐drug conjugates (ADCs).^[^ ^1^ ^]^ Consequently, significant efforts have been directed toward labeling proteins with useful functional groups.^[^ ^2^ ^]^

In the initial stages, chemical modification techniques utilizing nucleophilic residues in native proteins, such as Lys^[^ ^3^ ^]^ and Cys,^[^ ^3^, ^4^ ^]^ among others,^[^ ^5^ ^]^ were developed and widely adopted owing to their technical simplicity. Recently, site‐specific variants of these chemical modifications have emerged, addressing the issue of nonspecific labeling.^[^ ^6^ ^]^ Another well‐known approach involves protein modification using enzymes. This method allows for the selective introduction of desired functional groups at specific positions, facilitated by enzymes recognizing particular residues or amino acid sequences.^[^ ^7^ ^]^ A protein modification method employing genetic code expansion (GCE) technology has recently gained substantial popularity.^[^ ^8^ ^]^ This innovative technique enables protein modification through the manipulation of the cellular translation system, allowing the incorporation of non‐canonical amino acids (ncAAs) at predetermined positions within the target protein.^[^ ^9^ ^]^ These methods also find wide application in antibody labeling, enabling the introduction of biochemical probes, such as biotin or fluorophores, and drugs for ADC synthesis.^[^ ^1^, ^10^ ^]^ Although these methods are extensively utilized in diverse biochemical and pharmaceutical applications, each approach confers specific advantages and limitations. These limitations include nonspecific modifications, low modification efficiency or protein yield, and complexities associated with genetic manipulation.

**Figure 1:**
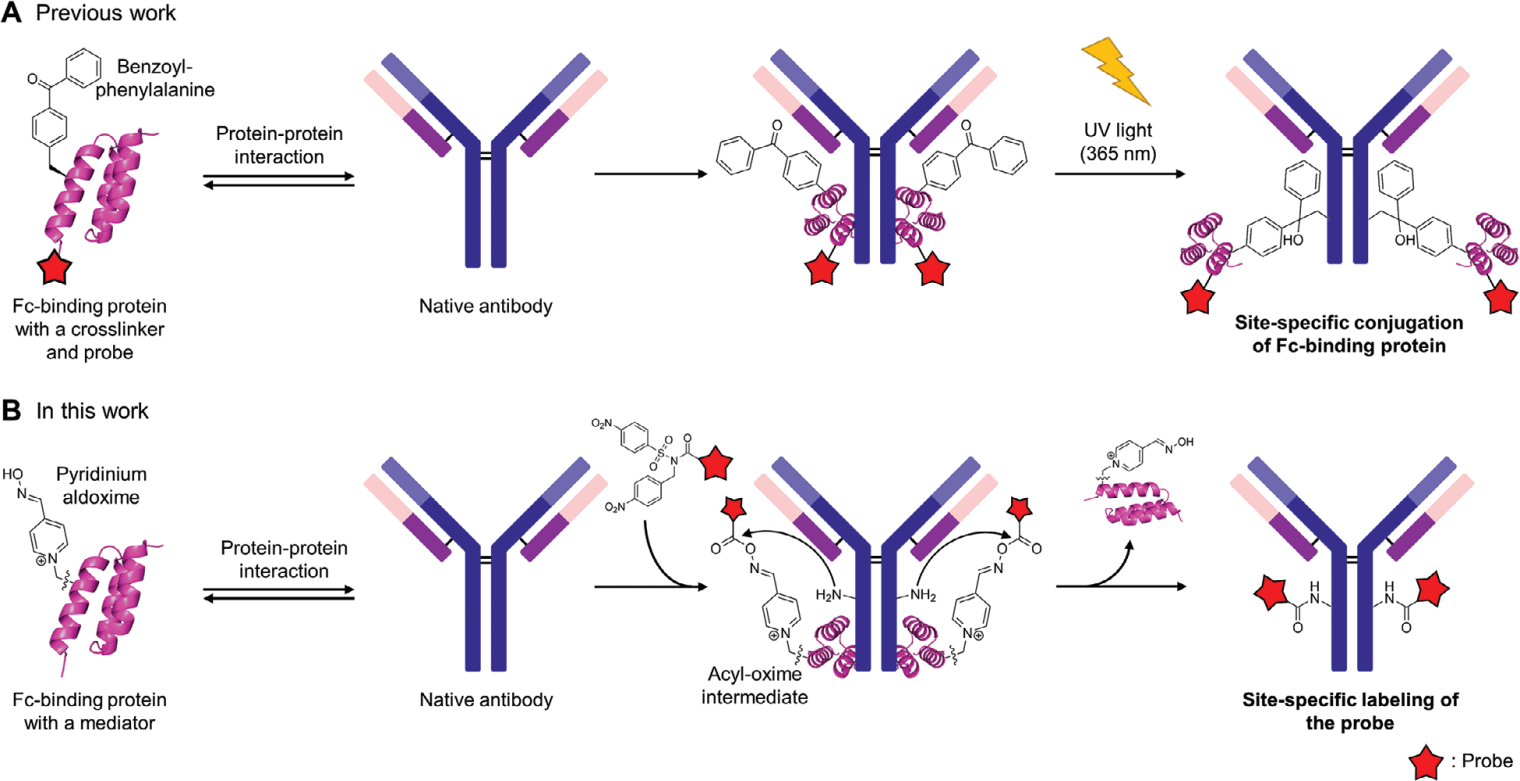
Previous and current work on affinity‐driven antibody labeling using Fc‐binding proteins. A) The labeling of a native antibody using cross‐linking of the entire Fc‐binding protein containing a probe. B) In this work, an Fc‐binding protein with a mediator is employed and facilitates the labeling reaction, allowing traceless labeling of an antibody.

To address these limitations, site‐specific modification methods for intact proteins have primarily been developed based on affinity‐driven strategies. In these methods, labeling agents are covalently attached to specific binders for a target protein. This positions the reactive moiety of the labeling agent close to the desired residue within the target protein for subsequent conjugation. The binding molecules utilized in this method include small molecules,^[^ ^11^ ^]^ nucleic acid aptamers,^[^ ^12^ ^]^ peptides,^[^ ^13^ ^]^ and proteins.^[^ ^14^ ^]^ Among these, proteins are noteworthy for their relatively high binding affinity and the availability of many binding proteins for a diverse range of interesting proteins, including antibodies. For instance, widely known binding proteins for antibodies, such as Staphylococcal Protein A and Protein G, have been extensively employed for affinity‐driven native antibody labeling.^[^ ^14a,b^ ^]^ In addition, new binding proteins with high affinity for useful target proteins can be obtained through protein engineering techniques such as phage display. However, applying binding proteins for native protein modification has been challenging owing to the technical limitation regarding the tendency of the binding proteins to remain attached to the target proteins (**Figure** **1**A).^[^ ^14c–i^ ^]^

In this study, we introduce an approach involving traceless affinity‐driven labeling of native proteins, in which small‐affinity proteins were used as mediators to efficiently transfer a chemical probe to a target protein (Figure 1B). Using small *α*‐helical proteins as a binding protein, Z‐domain protein (Z‐DM), Fc fragment, and trastuzumab were site‐specifically labeled with a fluorophore, biotin (Bt), and a drug. Excellent efficiency and site specificity were achieved, as confirmed through high‐performance liquid chromatography (HPLC) and mass spectrometry (MS) analyses. Using this method, a highly homogeneous ADC was generated by attaching a cytotoxic drug to trastuzumab; this ADC exhibited high cytotoxicity in breast cancer cell lines. Notably, this developed method allowed for the labeling of intact target proteins without necessitating genetic, chemical, or enzymatic modifications—an important advantage across various applications. In addition, the conjugation formed a stable and traceless amide bond with lysine, offering distinct benefits for pharmaceutical applications. Furthermore, many small helical proteins with high affinity for various important proteins are either readily available or can be easily engineered for specific target proteins, broadening their applications to a more diverse set of target proteins.^[^ ^15^ ^]^

## 2 Results and Discussion

### 2.1 Design of the Model System

**Figure 2:**
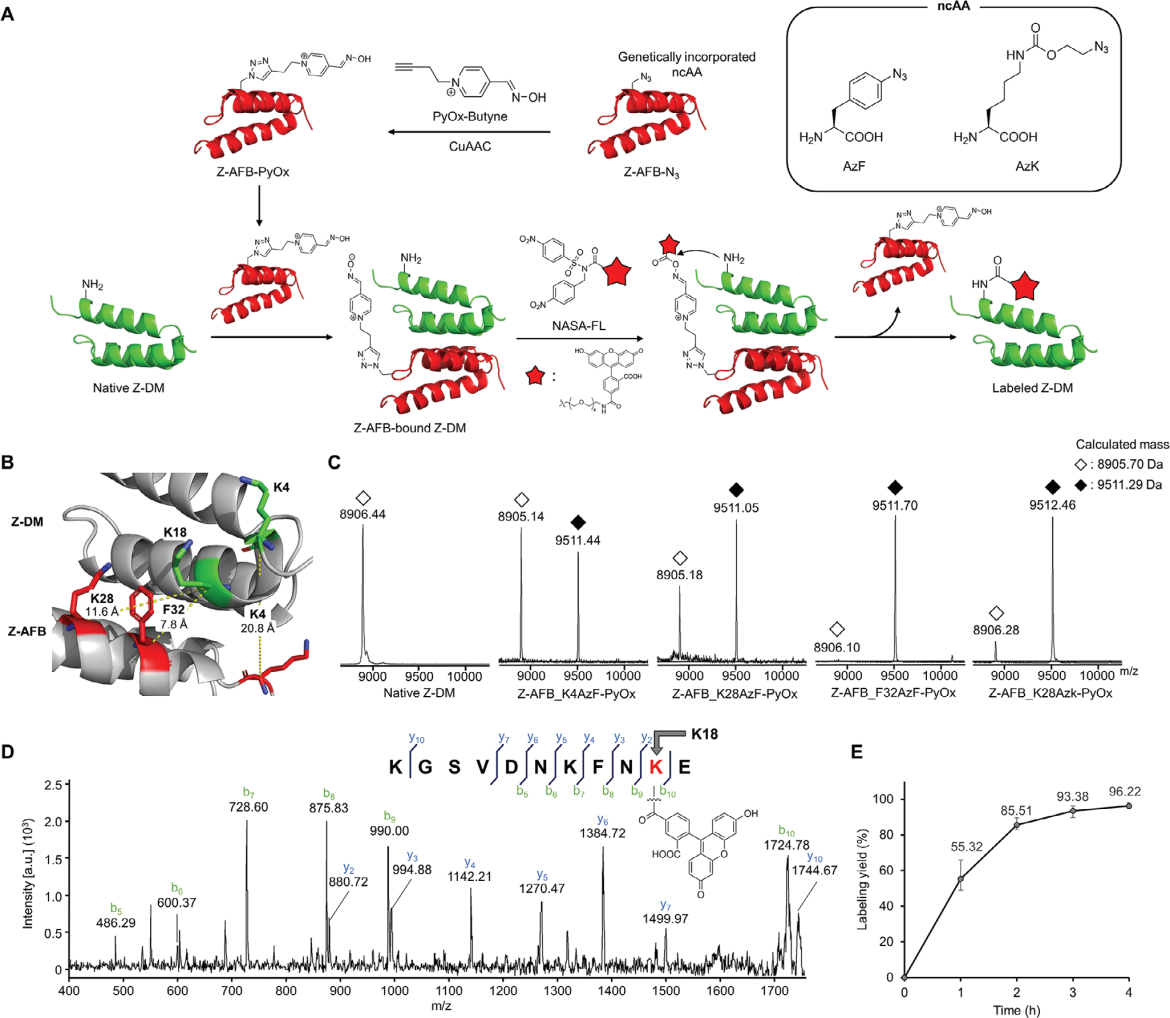
Labeling experiments for intact Z‐DM A) Workflow of the labeling process. A ncAA containing an azide group (AzF, AzK) was genetically incorporated into Z‐AFB. A subsequent CuAAC reaction was used to couple PyOx‐Butyne to Z‐AFB‐N_3_, yielding Z‐AFB‐PyOx. A mixture of Z‐DM and Z‐AFB‐PyOx was then combined, followed by the addition of NASA‐FL, resulting in the generation of labeled Z‐DM. B) Site selection for ncAA incorporation and PyOx conjugation. Three specific sites in Z‐AFB (F32, K28, and K4) were selected for azide‐containing ncAA incorporation and subsequent PyOx conjugation. These selected sites (F32, K28, and K4) were positioned close to K18, K18, and K9 in Z‐DM, respectively. C) MALDI‐TOF MS analysis revealed the outcomes of Z‐DM labeling reactions using each Z‐AFB‐PyOx mutant. Non‐labeled Z‐DM is represented as ◇ (calculated mass, 8905.70 Da), whereas FL‐labeled Z‐DM is denoted as ◆ (calculated mass, 9511.29 Da). D) MALDI‐TOF/TOF tandem MS analysis of the labeled peptide fragment obtained from Glu‐C digestion was conducted to demonstrate the labeling of K18. E) Time‐dependent labeling of Z‐DM using Z‐AFB_F32AzF‐PyOx. Reaction conditions: 50 mm HEPES pH 7.2, Z‐DM (15 µm), Z‐AFB‐PyOx (30 µm), and NASA‐FL (75 µm), 37 °C, and 4 h.

The study was undertaken to develop a method for site‐selective labeling of intact proteins. To achieve this, we considered the possibility of a proximity‐based labeling system employing a protein capable of binding to a target protein. This binding protein would act as a mediator, facilitating the selective reaction of the labeling with it to form a highly reactive intermediate near the target protein. This, in turn, would enable the formation of covalent bonds with adjacent lysine residues in the target protein. To ensure the mediating role of the binding protein, incorporating a mediating group capable of generating a reactive intermediate with the labeling agent was essential; therefore, we selected pyridinium aldoxime (PyOx) for this purpose (Figure 1B).^[^ ^16^ ^]^ PyOx rapidly reacts with *N*‐alkylsulfonamide (NASA) to form a reactive ester, which then efficiently forms amide bonds with amines.^[^ ^11b^ ^]^ To implement and optimize this labeling method, we considered a simplified protein complex system. We selected a variant of the B‐domain of Staphylococcal Protein A, Z‐DM,^[^ ^17^ ^]^ as the target protein and a Z‐domain‐specific affibody (Z‐AFB)^[^ ^18^ ^]^ as the binding protein (**Figure** **2**A). These proteins are small in size (≈8 kDa), and their high‐resolution complex structures are readily available, rendering them suitable for use as a model system. To introduce PyOx at a specific location on the Z‐AFB, we utilized GCE technology. This enabled the site‐specific incorporation of ncAA containing an azide group into the Z‐AFB. Subsequently, we used a Cu‐catalyzed azide–alkyne cycloaddition (CuAAC) reaction to attach PyOx to a specific Z‐AFB site (Figure 2A). We anticipated that this system would effectively enable the site‐specific conjugation of the probe onto the target protein through the simple mixing of three components: Z‐DM, Z‐AFB containing PyOx, and the probe derivatized with NASA (Figure 2A).

### 2.2 Labeling Experiments for Intact Z‐DM

The linchpin of the proposed model system hinges on Z‐AFB containing PyOx. To introduce PyOx at a specific position in Z‐AFB, the introduction of a ncAA into Z‐AFB was imperative. First, the positions for ncAA incorporation were selected based on the X‐ray crystal structure of the Z‐AFB and Z‐DM complex: F32, K28, and K4 were proximal to Lys in Z‐DM, without affecting the binding between Z‐AFB and Z‐DM (Figure 2B). The ncAA, 4‐azido‐l‐phenylalanine (AzF, Figure 2A), was first selected as the candidate to be introduced at these positions, as it is widely used for protein labeling through bioorthogonal reactions. To incorporate AzF into Z‐AFB, the codons corresponding to F32, K28, and K4 in Z‐AFB were modified to amber codons (TAG). The modified genes were expressed along with aminoacyl‐tRNA and aminoacyl‐tRNA synthetase (AzFRS) pairs^[^ ^9b^ ^]^ in the presence of AzF, resulting in Z‐AFB variants containing AzF (Z‐AFB_K4AzF, Z‐AFB_K28AzF, and Z‐AFB_F32AzF). Sodium dodecyl sulfate‐polyacrylamide gel electrophoresis (SDS‐PAGE) and matrix‐assisted laser desorption/ionization time‐of‐flight mass spectrometry (MALDI‐TOF MS) analyses were used to confirm the quantitative incorporation of AzF (Figures S1 and S2, Supporting Information). Next, we introduced PyOx into the AzF‐containing Z‐AFB via a CuAAC reaction. Butyne‐substituted PyOx (PyOx‐Butyne, Figure 2A) was reacted with Z‐AFB mutants under previously described conditions,^[^ ^19^ ^]^ and SDS‐PAGE and MALDI‐TOF MS analyses were employed to confirm the introduction of PyOx into all Z‐AFB variants (Z‐AFB_K4AzF‐PyOx, Z‐AFB_K28AzF‐PyOx, and Z‐AFB_F32AzF‐PyOx) (Figures S1 and S2, Supporting Information). As the labeling agent, NASA‐FL was prepared by linking fluorescein (FL) and NASA using a previously established method (Figure 2A).^[^ ^11b^ ^]^

We assessed the potential for site‐specific labeling of native Z‐DM using Z‐AFB‐PyOx and NASA‐FL. Labeling experiments were conducted by simply mixing Z‐DM with Z‐AFB‐PyOx and NASA‐FL. The labeling efficiency was determined using MALDI‐TOF MS analysis. Notably, distinct labeling efficiencies were observed across the three Z‐AFB‐PyOx variants: Z‐AFB_K4AzF‐PyOx and Z‐AFB_K28AzF‐PyOx exhibited a labeling efficiency of 35% and 70%, respectively, whereas Z‐AFB_F32AzF‐PyOx demonstrated the highest efficiency at 96% (Figure 2C; Figure S3, Supporting Information). These variations in labeling efficiency were strongly correlated with the proximity of PyOx introduced on Z‐AFB to the nearest Lys residue in Z‐DM. A clear trend emerged, wherein shorter distances between sites led to enhanced labeling efficiencies. To substantiate this distance‐dependent effect, we synthesized Z‐AFB_K28AzK‐PyOx, which incorporated *N^6^*‐[(2‐azidoethoxy)carbonyl]‐l‐lysine (AzK, Figure 2A) with an elongated and more flexible alkyl chain (Figure S4, Supporting Information). Under the same conditions, the labeling reaction exhibited a significantly heightened efficiency (93%) compared to that of Z‐AFB_K28AzF‐PyOx (70%), providing conclusive evidence for the distance‐dependent nature of the labeling reaction (Figure 2C; Figure S3, Supporting Information). Collectively, our established labeling approach demonstrated exceptional efficiency (96% using Z‐AFB_F32AzF‐PyOx) in labeling the native Z‐DM protein. Notably, the labeling efficiency can be further optimized by leveraging GCE technology to screen PyOx insertion sites and by adjusting the linker length within Z‐AFB‐PyOx.

We explored the possibility of directly labeling proteins with NASA by incorporating NASA into the binding protein. However, this approach resulted in a notable decrease in labeling efficiency due to the slow and spontaneous reactions between NASA and the lysine or N‐terminal amines on the binding protein (self‐labeling). While NASA exhibits rapid reactivity with PyOx, its reaction with adjacent amines is also observed in the absence of PyOx.

### 2.3 Confirmation of Labeled Residues in Z‐DM

Next, we assessed the correspondence of the labeled positions on Z‐DM to the predicted Lys residues based on the crystal structure. The FL‐labeled Z‐DM resulting from the labeling experiment with Z‐AFB_F32AzF‐PyOx was treated with a protease (Glu‐C). Subsequently, the resulting peptides were analyzed using MALDI‐TOF/TOF tandem MS to precisely determine the labeled positions. The results revealed that the labeling occurred at K18 of Z‐DM, which matched the predicted position based on the crystal structure (Figure 2D; Figures S5–S7, Supporting Information). This result demonstrates the effectiveness of the designed labeling system in labeling intact proteins and the predictability of labeling positions through structural analysis.

In addition, we measured the efficiency of Z‐DM labeling over various time periods. The results showed a progressive increase in K18‐labeled proteins up to 4 h after the reaction initiation. Over half of Z‐DM was labeled within 1 h, and by a 4 h mark, 96% of the protein was labeled (Figure 2E; Figure S8, Supporting Information). Upon extending the reaction time further, a minimal amount of labeling was observed at the N‐terminal amine (data not shown). However, for up to 4 h, labeling was predominantly observed on K18. When non‐binding proteins were subjected to the labeling reaction in the presence of Z‐AFB_F32AzF‐PyOx, little (<5%) to no labeling occurred, indicating that the labeling reaction requires the binding of the target protein to Z‐AFB_F32AzF‐PyOx (Figure S9, Supporting Information).

### 2.4 Experimental Design for Fc Fragment Labeling

**Figure 3:**
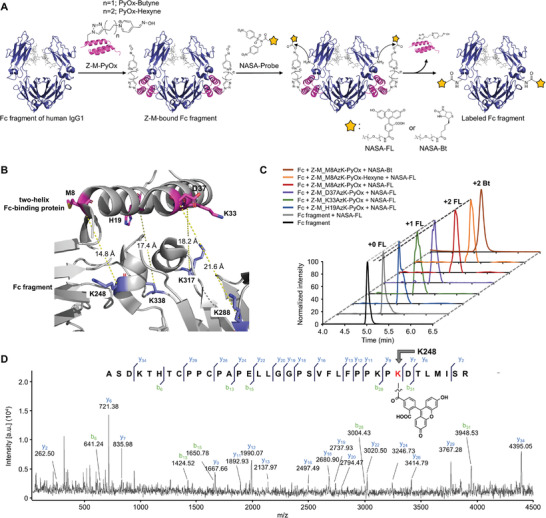
Experimental design and labeling results for the Fc fragment. A) Schematic representation of the labeling of the Fc fragment. In addition to PyOx–Butyne and NASA‐FL, PyOx–Hexyne and NASA–Bt were also subjected to analysis for comparison; the structural representation was derived from PDB ID 1H3X. B) Site selection for the incorporation of ncAAs and subsequent PyOx conjugation in Z‐M protein. The designated sites (M8, H19, K33, and D37) were positioned close to K248, K338, K317, and K288 in the Fc fragment, respectively; the image was derived from PDB ID 5U52. C) HPLC analyses of the labeling experiments for the Fc fragment using Z‐M variants and NASA probes (FL and Bt). D) The labeling of K248 is shown through MALDI‐TOF/TOF tandem MS analysis of the labeled‐peptide fragment obtained from trypsin digestion of FL‐labeled Fc fragment using Z‐M_M8AzK‐PyOx. Reaction conditions: Fc fragment (7.5 µm), Z‐M‐PyOx (60 µm), NASA‐FL or Bt (225 µm), and 50 mm HEPES (pH 8.0), 37 °C, and 6 h.

Subsequently, we labeled the Fc fragment of human immunoglobulin G1 (IgG1) (**Figure** **3**A). The Fc fragment was selected as the target protein before antibody labeling, as it facilitates easy analysis of the labeled product and optimization of reaction conditions. As the Fc fragment is conserved in various therapeutic antibodies, the labeling method developed for the Fc fragment can be directly applied to antibodies containing the same Fc fragment. To achieve site‐specific labeling of the Fc fragment, we searched for an Fc‐binding protein with high affinity and selectivity, after which we selected a modified two‐helix Fc‐binding protein,^[^ ^20^ ^]^ which we further optimized by introducing an additional helix to improve protein stability and expression. The design of this supplementary helix was based on the sequence of existing three‐helix Fc‐binding proteins.^[^ ^17^ ^]^ Through computational analysis using the AlphaFold2 program,^[^ ^21^ ^]^ we confirmed that the additional helix maintained the desired three‐helical structure (Figure S10, Supporting Information). We anticipated that the resultant three‐helix protein would exhibit high and specific binding affinity to the Fc fragment.

Next, using the designed Z‐M protein, we identified suitable positions to introduce PyOx. To determine these positions, we referred to the crystal structure of the complex formed by the two‐helix Fc‐binding protein and the Fc fragment,^[^ ^22^ ^]^ after which we selected M8, H19, K33, and D37 in the Z‐M protein for PyOx incorporation. These positions were expected to be proximal to the Fc fragment with minimal perturbations in Fc binding, ensuring effective labeling (Figure 3B). The distances between the selected residues in Z‐M and the nearest Lys on the Fc fragment were ≈15–22 Å, which was considerably farther compared to those measured for Z‐DM/Z‐AFB. Considering the correlation between labeling efficiency and the distance between the PyOx incorporation sites and the nearest Lys, we anticipated that AzK, instead of AzF, would be a more suitable ncAA for labeling the Fc fragment owing to its longer chain length (Figure S4, Supporting Information). Consequently, AzK was incorporated into Z‐M at positions M8, H19, K33, and D37 using GCE technology. The successful incorporation of AzK was confirmed through SDS‐PAGE and MALDI‐TOF MS, which showed its quantitative incorporation into Z‐M (Figures S11 and S12, Supporting Information). Subsequently, PyOx was introduced into each Z‐M variant through a CuAAC reaction, and its efficient conjugation was verified through SDS‐PAGE and MALDI‐TOF MS (Figures S11 and S12, Supporting Information).

Furthermore, we evaluated the binding affinity of Z‐M variants containing AzK or AzK‐PyOx to an antibody (trastuzumab) containing the same Fc fragment using surface plasmon resonance analysis. All variants showed nearly the same binding affinity compared to that of the Z‐M wild‐type (WT) protein (Figure S13 and Table S1, Supporting Information).^[^ ^22^ ^]^ This finding indicates that the incorporation of PyOx did not significantly impact the binding affinity between the Z‐M and Fc fragments.

### 2.5 Labeling and Characterization of the Intact Fc Fragment

We tested the labeling efficiency for the Fc fragment using the Z‐M variants containing PyOx. Initially, we screened for the optimal Z‐M variant that exhibited the highest labeling efficiency. Labeling reactions were performed by mixing each Z‐M variant and NASA‐FL with the Fc fragment, and the labeling efficiency was evaluated using HPLC. Z‐M_M8AzK‐PyOx exhibited the highest labeling efficiency, labeling the Fc fragment with little to no unlabeled product remaining (Figure 3C). In contrast, the other Z‐M variants yielded a significant amount of unlabeled Fc fragments. In the control experiment without Z‐M‐PyOx, almost no labeling was observed, indicating minimal background labeling (Figure 3C).

In contrast to Z‐DM labeling using Z‐AFB, the labeling efficiency in Fc fragment labeling with Z‐M was not strongly correlated with the distance between PyOx and the target Lys. While the distances between the PyOx sites and adjacent Lys were in the order M8 > H19 > K33 > D37, the labeling efficiency followed the sequence M8 > K33 > D37 > H19 (Figure 3B,C). Although the most efficient labeling occurred when PyOx was introduced at the closest position (M8), the next‐closest position (H19) exhibited the lowest labeling efficiency. This result can be explained through a close examination of the complex structure of the Fc fragment and Z‐M. K338 within the Fc fragment is a potential residue involved in the labeling reaction when Z‐M_H19AzK‐PyOx was used. In the complex structure, K338 was partially buried by neighboring residues, potentially causing structural hindrance to the labeling reaction (Figure S14, Supporting Information). Therefore, the efficiency of the labeling reaction using Z‐M_H19AzK‐PyOx was lower than that using Z‐M_D37AzK‐PyOx or Z‐M_K33AzK‐PyOx despite the shorter distance. This analysis underscores the importance of considering both the distance to the labeled Lys and the surrounding environment of the Lys to be labeled when selecting the PyOx incorporation site.

We performed additional labeling experiments using Z‐M_M8AzK‐PyOx, which showed the best labeling efficiency in the Fc fragment labeling experiments. NASA‐Bt, in addition to NASA‐FL, was also synthesized and used for labeling experiments (Figure 3A). HPLC analysis confirmed high labeling efficiency, similar to that of NASA‐FL (Figure 3C). Furthermore, we prepared Z‐M_M8AzK‐PyOx‐Hexyne, which has a two‐carbon longer alkyl chain in the PyOx linker, and analyzed its labeling efficiency (Figure 3A). Z‐M_M8AzK‐PyOx‐Hexyne exhibited a nearly quantitative labeling efficiency similar to that of Z‐M_M8AzK‐PyOx (Figure 3C). Given that Z‐M_M8AzK‐PyOx and Z‐M_M8AzK‐PyOx‐Hexyne showed efficient Fc fragment labeling, no further optimization of the PyOx linker length was conducted.

Next, we characterized the residues in the Fc fragment labeled using Z‐M_M8AzK‐PyOx. The complex structure of the Fc fragment with the Z‐M protein showed that the nearest Lys in the Fc fragment to M8 in Z‐M was K248, and another Lys residue, K246, was observed nearby (Figure S15, Supporting Information). To identify the labeled residue, the labeled Fc fragment was treated with trypsin, and the resulting peptide fragments were analyzed using MALDI‐TOF/TOF tandem MS. The peptide fragment containing K248 was clearly shifted to the corresponding labeled fragments for both the FL‐ and Bt‐labeled fragments, and MALDI‐TOF MS analysis revealed little to no unlabeled fragments (Figure S16, Supporting Information), as observed in the HPLC results (Figure 3C). MALDI‐TOF/TOF tandem MS analysis of the peptide fragment unequivocally identified K248 as the labeled residue without any indication of labeling at neighboring residues (e.g., K246) (Figure 3D; Figures S17–S19, Supporting Information).

### 2.6 Labeling Experiments for Intact Trastuzumab

**Figure 4:**
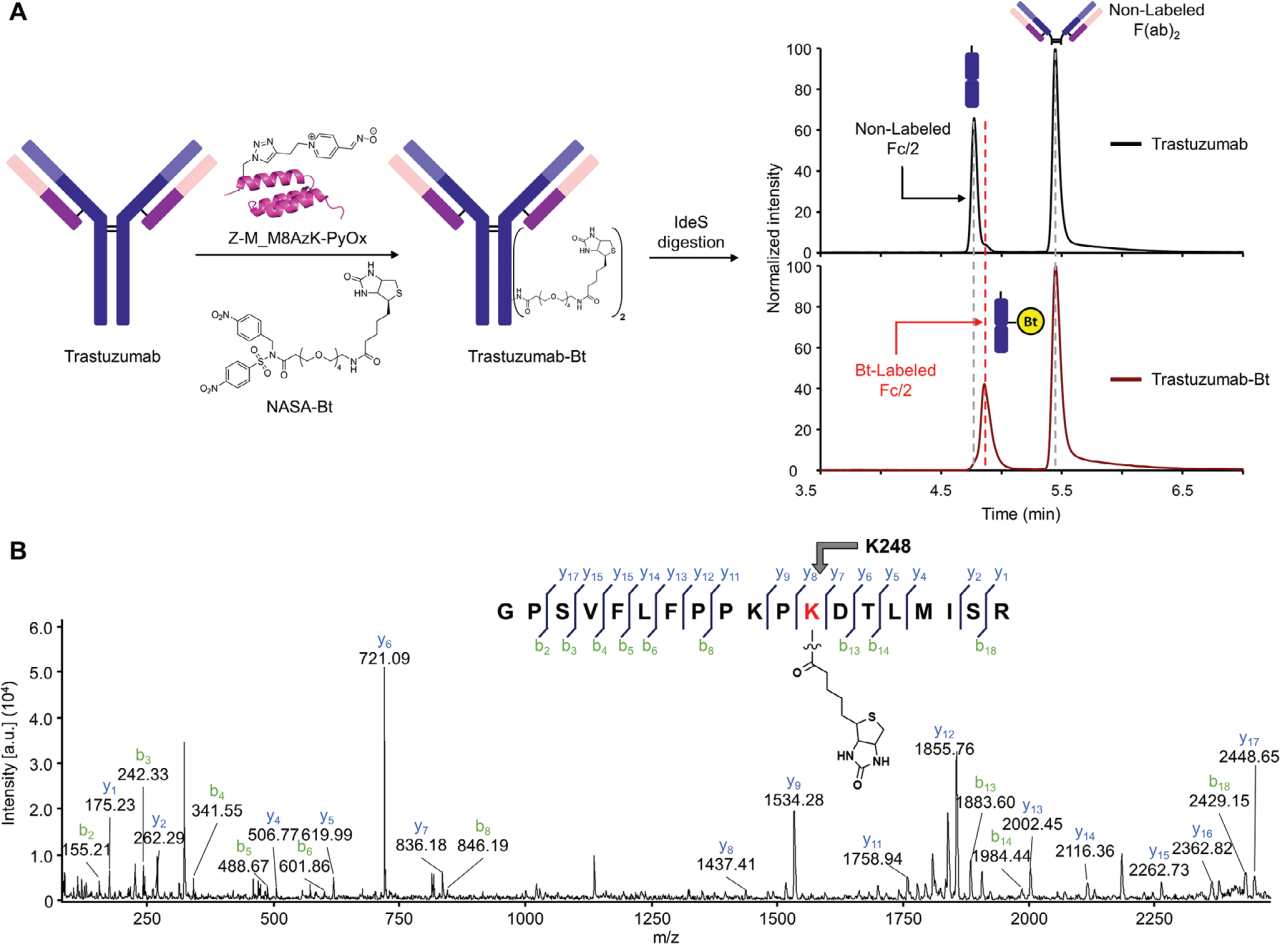
Labeling of intact trastuzumab. A) HPLC analyses of biotin labeling experiments for trastuzumab using Z‐M_M8AzK‐PyOx and NASA‐Bt; IdeS digestion were conducted after the reaction. B) The labeling of K248 is shown via MALDI‐TOF/TOF tandem MS analysis of the labeled peptide fragment obtained from trypsin digestion of Bt‐labeled trastuzumab. Reaction conditions: trastuzumab (5 µm), Z‐M‐PyOx (60 µm), NASA‐Bt (250 µm), 50 mm HEPES (pH 8.0), 37 °C, and 6 h.

We confirmed efficient and selective labeling of the Fc fragment using Z‐M_M8AzK‐PyOx. To apply this system for antibody labeling, we used trastuzumab, which specifically binds to human epidermal growth factor receptor 2 (HER2) and has been used for cancer therapy.^[^ ^23^ ^]^ Trastuzumab was subjected to a labeling reaction using Z‐M_M8AzK‐PyOx and NASA‐Bt, and the reaction was analyzed using HPLC. Given that the resolution was insufficient when analyzing the entire antibody using HPLC, the antibody was divided into F(ab)_2_ and Fc/2 fragments using IdeS before HPLC analysis.^[^ ^24^ ^]^ Only the Fc/2 peak shifted, confirming the specific labeling of the Fc fragment (**Figure** **4**A). Subsequently, MALDI‐TOF/TOF tandem MS analysis revealed specific biotin labeling at K248, consistent with the Fc fragment labeling results (Figure 4B; Figures S20–S23, Supporting Information).

**Figure 5:**
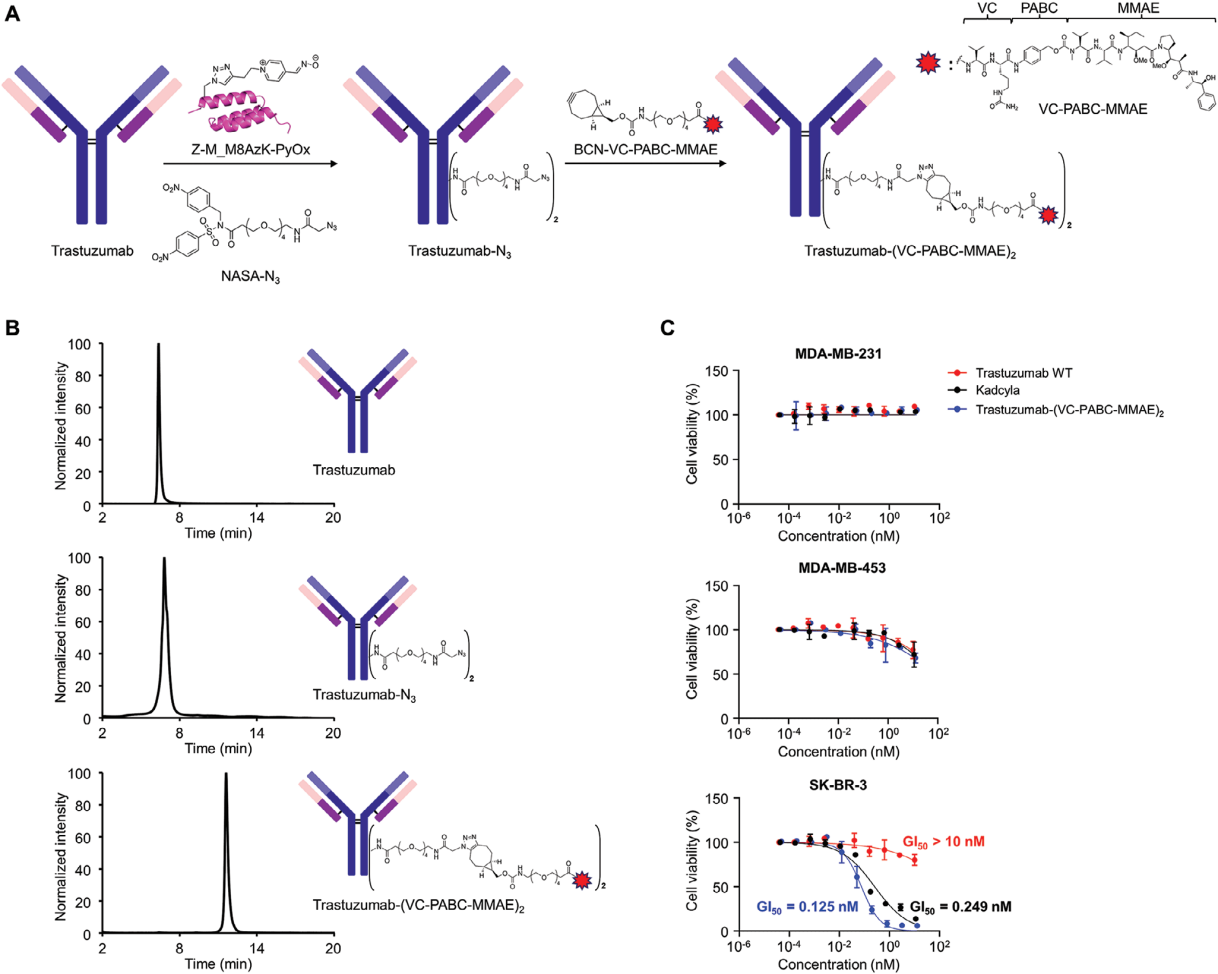
Production and assessment of ADCs. A) The schematic representation of the labeling of N_3_ and subsequent conjugation of BCN‐VC‐PABC‐MMAE to trastuzumab‐N_3_ for the production of the ADC, trastuzumab‐(VC‐PABC‐MMAE)_2_. B) Analytical hydrophobic interaction chromatography data for trastuzumab WT, trastuzumab‐N_3_, and trastuzumab‐(VC‐PABC‐MMAE)_2_. C) In vitro antitumor activity of trastuzumab WT, Kadcyla, and trastuzumab‐(VC‐PABC‐MMAE)_2_ against MDA‐MB‐231, MDA‐MB‐453, and SK‐BR‐3.

Next, we labeled trastuzumab with a cytotoxic drug to synthesize ADC. Using monomethyl auristatin E (MMAE), the most widely used drug in FDA‐approved ADCs, we synthesized an ADC using a valine‐citrulline‐*p*‐aminobenzyloxycarbonyl (VC‐PABC) linker derivatized with NASA for conjugation. However, complications arising from the solubility of NASA‐VC‐PABC‐MMAE impeded direct incorporation into trastuzumab. Consequently, we devised an alternative strategy: the introduction of an azide moiety into trastuzumab using NASA‐N_3_, followed by strain‐promoted azide–alkyne cycloaddition (SPAAC) to incorporate VC‐PABC‐MMAE (**Figure** **5**A). The trastuzumab labeling reaction was performed using Z‐M_M8AzK‐PyOx and NASA‐N_3_, which resulted in efficient azide labeling of trastuzumab, albeit at a slightly lower efficiency than that of NASA‐Bt (Figures S24–S26, Supporting Information). MALDI‐TOF/TOF tandem MS analysis confirmed the labeled position (K248) to be identical to that observed in Fc fragment labeling (Figures S27 and S28, Supporting Information).

To incorporate VC‐PABC‐MMAE into trastuzumab‐N_3_, bicyclo[6.1.0]non‐4‐yne (BCN), which is widely used in SPAAC reactions, was selected as the strained alkyne group, and BCN‐VC‐PABC‐MMAE was prepared for subsequent conjugation (Figure 5A). Next, trastuzumab‐N_3_ and BCN‐VC‐PABC‐MMAE were subjected to SPAAC reaction to produce trastuzumab‐(VC‐PABC‐MMAE)_2_, and the synthesized ADC underwent fast protein liquid chromatography purification to yield homogeneous trastuzumab‐(VC‐PABC‐MMAE)_2_ product (Figure 5B; Figure S29, Supporting Information).

### 2.7 Cell Viability Assay

To assess the cytotoxicity of the synthesized ADC, we performed cell viability assays using established breast cancer cell lines. Three cell lines were used for these assays: two were HER2‐positive cells (SK‐BR‐3: high, MDA‐MB‐453: low), whereas one was a HER2‐negative cell (MDA‐MB‐231) (Figure S30, Supporting Information). The cells were treated with trastuzumab‐(VC‐PABC‐MMAE)_2_, trastuzumab WT, and Kadcyla. Trastuzumab‐(VC‐PABC‐MMAE)_2_ was highly effective against HER2‐positive SK‐BR‐3 cells (Figure 5C; Table S2, Supporting Information). Furthermore, the developed ADC showed slightly higher efficacy than the FDA‐approved ADC Kadcyla (Figure 5C; Table S2, Supporting Information). Conversely, HER2‐negative MDA‐MB‐231 cells did not exhibit cytotoxicity, whereas HER2‐low expression MDA‐MB‐453 cells showed moderate cytotoxicity (Figure 5C; Table S2, Supporting Information). Additionally, the binding affinity of trastuzumab‐(VC‐PABC‐MMAE)_2_ for both HER2 and FcRn was measured to confirm that the labeling of the antibody with the drug did not affect its binding affinity. The results showed that the binding affinity was similar to that of trastuzumab WT (Figures S31 and S32, Tables S3 and S4, Supporting Information). These results demonstrate that the ADC generated using our labeling strategy exhibited high cancer cell‐specific cytotoxicity and intact binding affinity for FcRn, suggesting its potential for future therapeutic applications.

## 3 Conclusion

In this study, we established a novel method for labeling target proteins. This method leveraged small binding proteins, which acted as mediators to efficiently and site‐specifically transfer a chemical probe. By employing small alpha‐helical proteins as binding agents, we successfully labeled the Z‐DM protein, Fc fragment, and trastuzumab with various probes, including fluorophores, biotin, and a drug in a site‐specific manner. The labeling efficiency and conjugation sites were extensively evaluated, and excellent efficiency and site‐specificity were confirmed through MS analysis. Furthermore, this method yielded a HER2‐targeting ADC conjugated with MMAE, which exhibited high cytotoxicity and selectivity against breast cancer cell lines with varying levels of HER2 expression. This method offers an important advantage as it enables intact protein labeling without any modifications, rendering it highly valuable for diverse applications. In addition, the resulting labeling formed a stable and traceless amide bond with lysine, which is crucial for pharmaceutical applications. Moreover, many small helical‐binding proteins with a strong affinity for various important proteins are readily available and can be easily engineered for specific target proteins using common techniques, such as phage display.^[^ ^15^ ^]^ This expandability broadens the range of proteins to which this method can be applied. Overall, this study introduces a promising approach for native protein labeling using small binding proteins, offering high site‐specificity and efficient transfer of chemical probes. Its advantages, including intact protein conjugation and traceless amide bond formation, hold significant potential for various applications, particularly in the field of protein‐based therapeutics.

## Conflict of Interest

T.S. is a shareholder of MagicBullet Therapeutics Inc.

## Supporting information

Supporting Information

## Data Availability

The data that support the findings of this study are available from the corresponding author upon reasonable request.
