# Supplementary material for: Affinity‐Directed Site‐Specific Protein Labeling and Its Application to Antibody‐Drug Conjugates
Source: Adv Sci (Weinh). 2023 Nov 30;11(4):2306401. doi: 10.1002/advs.202306401 (PMC10811483; doi:10.1002/advs.202306401)
Supplement: Supplementary file 1 — Supporting Information [file ADVS-11-2306401-s001.pdf]

ADVANCED SCIENCE
Open Access

Supporting Information

for *Adv. Sci.*, DOI 10.1002/advs.202306401

Affinity-Directed Site-Specific Protein Labeling and Its Application to Antibody-Drug Conjugates

Sooin Kim, Sanggil Kim, Sangji Kim, Namkyoung Kim, Sang Won Lee, Hanbin Yi, Seungeun Lee, Taebo Sim, Yongseok Kwon* and Hyun Soo Lee**

Supporting Information
©Wiley-VCH 2021
69451 Weinheim, Germany

Affinity-Directed Site-Specific Protein Labeling and Its Application to Antibody-Drug Conjugates

Sooin Kim, Sanggil Kim, Sangji Kim, Namkyoung Kim, Sang Won Lee, Hanbin Yi, Seungeun Lee, Taebo Sim,* Yongseok Kwon,* and Hyun Soo Lee*

Table of Content

1. Materials and instrumentation S3
1.1. *Materials S3*
1.2. *Instrumentation S4*
2. Experimental procedures S5
2.1. *Protein preparations S5*
2.2. *Procedures for protein labeling S6*
2.3. *Determination of labeled residues S7*
2.4. *Determination of binding affinity using SPR S8*
2.5. *Determination of cytotoxicity of ADC products S9*
3. Organic synthesis S10
3.1. *Synthesis of PyOx-Butyne S10*
3.2. *Synthesis of PyOx-Hexyne S10*
3.3. *Synthesis of tert-butyl (15-((4-nitrophenyl)sulfonamido)-15-oxo-3,6,9,12-tetraoxapentadecyl)carbamate (S1) S11*
3.4. *Synthesis of tert-butyl (1-(4-nitrophenyl)-2-((4-nitrophenyl)sulfonyl)-3-oxo-6,9,12,15-tetraoxa-2-azaheptadecan-17-yl)carbamate (S2) S11*
3.5. *Synthesis of NASA-FL S12*
3.6. *Synthesis of NASA-Bt S12*
3.7. *Synthesis of NASA-N_3 .... S13*
3.8. *Synthesis of Fmoc-VC-PABC-MMAE S14*
3.9. *Synthesis of NH_2-VC-PABC-MMAE S14*
3.10. *Synthesis of BCN-VC-PABC-MMAE S15*
4. Supplementary figures and tables S16
4.1. *Supplementary figures S16*
4.2. *Supplementary tables S34*
5. Spectral data S36
6. References S46

1. Materials and instrumentation

1.1. Materials

Chemicals were purchased from commercial suppliers and used without any additional purification steps. For non-canonical amino acids (ncAAs) incorporation, *N*^{6}-[(2-azidoethoxy)carbonyl]-L-lysine (AzK) was purchased from Iris Biotech (Bayern, Germany) and 4-azido-L-phenylalanine (AzF) was purchased from Santa Cruz Biotechnology (Dallas, TX, USA). For the protein expression, NaCl, Na_2HPO_4, KH_2PO_4, $(NH_4)_2SO_4$, $MgSO_4$, $CuSO_4$, NaOH, ethylenediaminetetraacetic acid (EDTA), glycerol, sodium-L-ascorbate, aminoguanidine hydrochloride, and amino acids were purchased from Samchun Chemicals (Seoul, Korea). Lactose, iodoacetamide (IAA), and sodium dodecyl sulfate (SDS) were purchased from Sigma-Aldrich (St. Louis, MO, USA). Arabinose and chloramphenicol were purchased from Alfa Aesar (Haverhill, MA, USA). Ampicillin, kanamycin, and dithiothreitol (DTT) were purchased from Fisher BioReagent (Pittsburgh, PA, USA), Daejung Chemicals (Siheung, Korea), and BioBasic (Markham, ON, Canada), respectively. HEPES, LB broth, and Ni-NTA agarose were purchased from Dojindo Laboratories (Kumamoto, Japan), Gibco (Grand Island, NY, USA), and Qiagen (Hilden, Germany), respectively. 2-(4-((bis((1-(tert-butyl)-1H-1,2,3-triazol-4-yl)methyl)amino)methyl)-1H-1,2,3-triazol-1-yl)acetic acid (BTTAA) was purchased from Jena Bioscience (Thuringen, Germany). For matrix-associated laser desorption ionization time-of-flight (MALDI-TOF) analysis, sinapic acid (SA), α-cyano-4-hydroxycinnamic acid (CHCA), and 2,5-dihydroxybenzoic acid (DHB) were purchased from Sigma-Aldrich. For high-performance liquid chromatography (HPLC) analysis, HPLC-grade acetonitrile (ACN), formic acid, and trifluoroacetic acid (TFA) were purchased from Samchun Chemicals. For chemical synthesis, 4-nitrobenzenesulfonamide and 4-nitrobenzyl bromide were purchased from Combi-Blocks (San Diego, CA, USA). 5(6)-Carboxyfluorescein was purchased from Sigma-Aldrich. Pyridine-4-carboxaldoxime, 4-bromo-1-butyne, 6-chloro-1-hexyne were purchased from Alfa Aesar. 2-Azidoacetic acid and biotin were purchased from Tokyo Chemical Industry (Tokyo, Japan). Fmoc-Val-Cit-PABC-PNP, monomethyl auristatin E (MMAE), and BCN-PEG4-acid were purchased from Angene (London, UK), Chemscene (Monmouth Junction, NJ, USA), and BLD Pharm (Cincinnati, OH, USA), respectively. For protein labeling experiments, trastuzumab was purchased from Roche (Basel, Switzerland). IdeS enzyme was purchased from ACROBiosystems (Newark, DE, USA). Sequencing Grade Modified Trypsin was purchased from Promega (Madison, WI, USA). Endoproteinase Glu-C was purchased from Worthington Biochemical (Lakewood, NJ, USA). For DNA manipulations, PrimeSTAR HS DNA Polymerase and restriction enzymes were purchased from Takara Bio (Kusatsu, Japan). DNA oligonucleotides were purchased from Macrogen (Seoul, Korea). PCR/Gel purification kit and plasmid mini-prep kit were purchased from Nucleogen (Siheung, Korea). For protein sample preparations, Amicon® Ultra centrifugal filter, and ZipTip® Pipette Tips were purchased from Merck Millipore (Burlington, MA, USA). Pierce™ C18 Spin Columns and Slide-A-Lyzer™ Dialysis Cassettes were purchased from Thermo Fisher Scientific (Waltham, MA, USA). PD SpinTrap G-25 and Hitrap Protein A HP column were purchased from Cytiva (Marlborough, MA, USA). PEI-Transfection reagent was purchased from Polysciences (Warrington, PA, USA). For cytotoxicity assays, MDA-MB-231, MDA-MB-453, and SK-BR-3 were purchased from KCLB (Seoul, Korea). Penicillin/streptomycin and fetal bovine serum (FBS) were purchased from Welgene (Gyeongsan, Korea). HER2 (#2165) and GAPDH (#5174) primary antibodies were purchased from Cell signaling technology (Danvers, MA,

USA). Protease inhibitor cocktail (#11873580001) and phosphatase inhibitor cocktail (#04906837001) were purchased from Roche. CellTiter Glo (G7572) and HRP-conjugated goat anti-Rabbit secondary antibody (SA002-500) were purchased from Promega and Gendepot (Barker, TX, USA), respectively.

1.2. Instrumentation

Analytical HPLC analysis

For reversed-phase liquid chromatography (RPLC), each sample (3 μg) was analyzed using Agilent 1260 Infinity I with Poroshell 300SB-C8 (Agilent, 1.0 × 75 mm, 5 μm) at a flow rate of 1 mL/min. Initially, 80% of mobile phase A (0.1% TFA in water) was kept for 2 min. Mobile phase B (0.1% TFA in can) then increased from 20% to 60% at 2 min to 8 min. For an additional 2 min (from 8 min to 10 min), mobile phase B increased from 60% to 98% to elute all the samples in the column. The samples were detected by a variable wavelength detector (VWD) at 280 nm.

For hydrophobic interaction chromatography (HIC), each sample (10 μg) was analyzed using the same HPLC with MAbPac HIC-Butyl HPLC (Thermo Fisher Scientific, 4.6 × 100 mm, 5 μm) at a flow rate of 1 mL/min. Initially, 100% of buffer A (50 mM sodium phosphate, pH 7.0, 1.5 M ammonium sulfate) was kept for 1 min. Buffer B (50 mM sodium phosphate, pH 7.0, 20% IPA(v/v)) then increased from 0% to 100% at 1 min to 15 min. For an additional 5 min, 100% of buffer B was kept, eluting all the samples in the column. The samples were detected by VWD at 280 nm.

Mass analysis of Fc fragment and trastuzumab

Each sample (30 μg) was cleaned up with PD SpinTrap G-25 with ammonium bicarbonate buffer (50 mM) and treated with 30 units of IdeS enzyme prior to the analysis. The samples were analyzed using a linear ion-trap Orbitrap mass spectrometer (LTQ Orbitrap XL, Thermo Fisher Scientific) with a combination of ultra-high performance liquid chromatography, Ultimate 3000 (UHPLC, Thermo Fisher Scientific), using ACQUITY UPLC Protein BEH C4 Column (Waters, 300 Å, 2.1 × 50 mm, 1.7 μm). For the UHPLC method, mobile phase A (0.1% TFA in water) was initially kept at 80% for 1 min. Mobile phase B (0.1% TFA in ACN) then increased from 20% to 90% at 1 min to 10 min. Orbitrap-MS was detected with an extended mass range from 500 to 4000 m/z. Deconvolution of the peaks was conducted using UniDec software.[1]

Purification of ADC using HIC

To purify trastuzumab-(VC-PABC-MMAE)$_2$, a HIC column prepacked with Skillpak Phenyl-650S HIC resin (Tosoh Bioscience, 0.8 × 10 cm, 5 mL) was employed and connected to AKTA pure 25 (Cytiva) (Advanced Bio-interface Core Research Facility). The entire process was conducted at room temperature. Prior to sample injection, the column was equilibrated with 20 column volumes of buffer A (50 mM sodium phosphate, pH 7.0, 2 M NaCl). To load onto the column, 0.25 mL of unpurified ADC (1 mg/mL in formulation buffer) was mixed with 0.25 mL of buffer A. This resulting mixture (total volume of 0.5 mL) was then injected into the column and eluted using a linear gradient from 100% buffer A to 100% buffer B (50 mM sodium phosphate, pH 7.0, 20% IPA(v/v)).

NMR

^{1}H NMR spectra were recorded on Bruker 400 MHz spectrometers at ambient temperature and spectra were processed using MestReNova 6.0.2 using the automatic phasing and polynomial baseline correction capabilities. Otherwise, ^{1}H NMR spectra were recorded on Varian 400 MHz spectrometers. Routine ^{13}C NMR spectra were recorded on Bruker 400 MHz or Bruker 700 MHz spectrometers with protons fully decoupled. ^{13}C Resonances are reported in ppm relative to solvent residual peaks for $CDCl_3$ (77.16 ppm).

FTIR

Infrared spectra were recorded on a JASCO FT/IR-4600 spectrometer and ν_{max} are partially reported in cm^{-1}. High-resolution mass spectra were acquired on a JEOL JMS-700 instrument with an FAB mode. Analytical thin-layer chromatography was performed using 60 Å Silica Gel F_{254} pre-coated plates (0.25 mm thickness). TLC plates were visualized by irradiation with a UV lamp. Normal-phase column chromatography was performed using 60 Å Silica Gel (32–62 micron) with an appropriate mobile phase composition and gradient. Normal-phase high-performance liquid chromatography was performed using an Agilent 1260 series instrument equipped with a diode array detector and columns from COSMOSIL.

2. Experimental procedures

2.1. Protein preparations

Protein expression, ncAA incorporation, and purification in *E. coli*

To obtain Z-DM protein, the Z-DM gene was created by amplifying the Z-domain WT gene, which was from a commercial source by gene synthesis, and overlapping with strep-tag sequence (GWSHPQFEK) at the N-terminus and a His_6-tag at the C-terminus. The Z-DM gene was then inserted between the NdeI and EcoRI sites of pET20b to generate pET20b-Z-DM. The plasmid was then transformed into *Escherichia coli* BL21(DE3) cell and amplified overnight at 37 °C in 6 mL LB broth with ampicillin (100 µg/mL). Amplified cells (1 mL) were inoculated to defined media[2] (50 mM Na_2HPO_4, 50 mM KH_2PO_4, 25 mM $(NH_4)_2SO_4$, 2 mM $MgSO_4$, 0.1% trace metals, 0.5% glycerol, 0.05% glucose, 0.2% lactose, and 5% amino acids) with the same amount of ampicillin, and the cells were incubated overnight at 37 °C. Cells were harvested by centrifugal force (10000 rpm, 5 min) and cell pellets were frozen at -20 °C for further purification. The pellets were thawed and purified with Ni-NTA agarose resin according to the manufacturer's protocol. The concentration of purified proteins was measured by UV absorbance at 280 nm and purity was inspected by 8-12% SDS-PAGE. The molar extinction coefficient of the protein was calculated from Biomol Protein Extinction Coefficient Calculator (http://www.biomol.net/en/tools/proteinextinction.htm).

The Z-AFB gene was also from a commercial source by gene synthesis and a His_6-tag was attached at the C-terminus using overlapping PCR. The Z-M gene was created from the minimized Z-domain of protein A[3] with the third helix of Z-domain WT. Also, a His_6-tag was added to the C-terminus of the gene. The Z-AFB gene was inserted between NcoI and KpnI sites of pBAD and the Z-M gene was inserted between NdeI and EcoRI of pET20b, generating pBAD-Z-AFB and pET20b-Z-M, respectively. pBAD-Z-AFB variants (K4, K28, F32TAG), and pET20b-

Z-M variants (M8, H19, K33, D37TAG) were generated by site-directed mutagenesis. For pBAD-Z-AFB_F32TAG, pET20b-Z-M_M8TAG, and D37TAG, the nearest lysines were substituted to Arg (CGT codon) in order to avoid the self-labeling of the probe, creating pBAD-Z-AFB_K28CGT_F32TAG, pET20b-Z-M_K5CGT_M8TAG, and pET20b-Z-M_K33CGT_D37TAG, respectively. The plasmids were then co-transformed with pEvol-AzFRS[4] or pEvol-AzKRS[5] in DH10B or BL21 cells. Transformed cells were expressed and purified the same way as described above with ncAA (100 μM of AzF or 1 mM of AzK) and chloramphenicol (35 μg/mL). Concentration and purity were also inspected by the same methods.

Expression and purification of IgG1 Fc fragment

The Fc fragment gene was amplified by PCR and then inserted between the Xho1 and BamH1 sites of pCDNA3.4 to generate pCDNA3.4-Fc fragment. The plasmid containing the Fc fragment was transiently transfected into FreeStyleTM 293 cells using PEI-Transfection reagent according to the manufacturer's protocol. For expression, the supernatant of FreeStyleTM 293 cells transiently transfected with the respective Fc fragment construct was harvested by centrifugation at 8,000 rpm for 20 min at 4 °C. The expressed Fc fragment was purified using a Hitrap Protein A HP column according to the manufacturer's protocol. The column was equilibrated with buffer A (1.8 mM KH_2PO_4, 10 mM K_2HPO_4, 137 mM NaCl, 2.7 mM KCl, pH 7.4). The supernatant containing the Fc fragment was loaded onto the column, and after washing to remove unbound impurities, the purified Fc fragment was eluted with buffer B (100 mM citrate, 100 mM NaCl, pH 3.0). Purified Fc fragments were dialyzed against A buffer three times to remove any residual purification buffers. The concentrations of Fc fragments were determined by measuring absorbance at 280 nm using the molar extinction coefficient (7.157×10^4 $cm^{-1}M^{-1}$) of the Fc fragment.

Copper(I)-catalyzed azide-alkyne cycloaddition (CuAAC)

Purified Z-AFB and Z-M variant proteins were dialyzed against 20 mM potassium phosphate buffer (100 mM NaCl, pH 7.0) using Slide-A-Lyzer™ Dialysis Cassettes, 3.5K MWCO. A mixture of 20 μM of each protein, PyOx (4 mM), $CuSO_4$:BTTAA premix (300 μM:1500 μM), sodium-L-ascorbate (5 mM), and aminoguanidine hydrochloride (5 mM) was incubated for 1h at 25 °C with 250 rpm stirring. The reaction mixture was dialyzed against 50 mM HEPES buffer (pH 7.2 or 8.0) and concentrated with Amicon® Ultra centrifugal filter, 3K MWCO for further reactions.

2.2. Procedures for protein labeling

Labeling of NASA-FL to Z-DM

Z-AFB variants containing PyOx (30 μM) were mixed with Z-DM protein (15 μM). NASA-FL (75 μM) was then added to the mixture and incubated for 4 h in HEPES buffer (50 mM, pH 7.2) at 37 °C. The reaction was quenched by a mixture of L-Lys and PyOx-Butyne at final concentrations of 5 mM. Labeled products were desalted with ZipTip with 0.6 μL C18 resin according to the manufacturer's protocol and the labeling yields were determined by MALDI-TOF MS analysis (matrix: sinapic acid). To further isolate labeled Z-DM from Z-AFB, purification using Strep•Tactin® Superflow™ Agarose (Novagen, Germany) resin could be used according to the manufacturer's protocol.

Labeling of NASA-FL or Bt to Fc fragment

Z-M variants with PyOx (60 μM) were mixed with Fc fragment (7.5 μM). NASA-probe (225 μM) was then added to the mixture and incubated for 6 h in HEPES buffer (50 mM, pH 8.0) at 37 °C. The reaction was also quenched in the same way as the previous reaction. To remove unreacted NASA-probe, the labeled products were cleaned up with PD SpinTrap G-25 for further analysis.

Labeling of NASA-Bt to Trastuzumab

Z-M M8AzK-PyOx (60 μM) were mixed with trastuzumab (5 μM). NASA-Bt (250 μM) was then added to the mixture and incubated for 6 h in HEPES buffer (50 mM, pH 8.0) at 37 °C. The reaction was also quenched in the same way as the previous reaction. To remove unreacted NASA-Bt, the labeled products were cleaned up with PD SpinTrap G-25 for further analysis.

Production of ADC

To produce ADCs, trastuzumab (5 μM) and Z-M M8AzK-PyOx (60 μM) were mixed. NASA-N_3 (60 μM) was then added to the mixture and incubated for 6 h in HEPES buffer (50 mM, pH 8.0) at 37 °C, producing trastuzumab-N_3. After the conjugation reaction, the reaction mixture was quenched with a solution containing L-Lys and PyOx-Butyne, with final concentrations of 5 mM each. Subsequently, we washed the mixture using a citric acid buffer (100 mM, pH 3.5) and processed it through an Amicon® Ultra centrifugal filter with a 50 kDa MWCO to eliminate any remaining Z-M M8AzK-PyOx. The resulting product was then exchanged and recovered by dialysis against PBS (pH 7.4) in preparation for further Strain-Promoted Azide-Alkyne Cycloaddition (SPAAC) reactions. The recovered product, trastuzumab-N_3 (10 μM), was mixed with BCN-MMAE (50 μM) and subjected to SPAAC reaction for 36 h at 25 °C. The product was then exchanged against PBS for further purification using FPLC.

2.3. Determination of labeled residues

Protease digestion of WT and FL-labeled Z-DM

WT and FL-labeled Z-DM proteins (15 μg) were cleaned up with PD SpinTrap G-25 with ammonium bicarbonate buffer (50 mM). Urea (final concentration of 6M) was added to the reaction mixture and incubated for 45 min at 50 °C. Endoproteinase Glu-C was added at a final concentration of 2 μM and incubated overnight at 37 °C. The reaction mixture was quenched by 0.4% of TFA and further purification was conducted with pierce™ C18 Spin Columns. The purified products were concentrated by solvent evaporation using N_2 blowing. For sample examination, the concentrated products were equivalently mixed with DHB. MALDI-TOF MS was used for finding labeled peptide fragments and MALDI-TOF/TOF tandem MS for the determination of labeled residue.

Protease digestion of WT and probe-labeled Fc fragment

WT and labeled Fc fragment (30 μg) were cleaned up with PD SpinTrap G-25 with ammonium bicarbonate buffer (50 mM) containing 0.1% SDS. DTT was added at a final concentration of 20 mM and incubated for 20 min at 95 °C.

Subsequently, IAA was added at a final concentration of 50 mM and incubated for 30 min at 25 °C in the dark and the excess IAA was quenched by 5 mM DTT. Sequencing grade trypsin was added to 1/20 of the amount of the Fc fragment and incubated overnight at 37 °C. The reaction mixtures were quenched by 0.4% of TFA and purified with C18 spin columns. The samples were also concentrated by solvent evaporation using N_2 blowing product and were equivalently mixed with CHCA for the examination. MALDI-TOF MS was used for finding labeled peptide fragments and MALDI-TOF/TOF tandem MS for the determination of labeled residue.

Protease digestion of WT and probe-labeled antibody

WT and labeled trastuzumab (30 μg) were cleaned up with PD SpinTrap G-25 with ammonium bicarbonate buffer (50 mM) and treated with 30 units of IdeS enzyme. After the treatment, 0.1% SDS was added to the samples and Sequencing grade trypsin was added to 1/20 of the amount of the antibody. The sample preparations for MALDI-TOF MS and MALDI-TOF/TOF tandem MS were conducted the same as Fc fragment analysis.

2.4. Determination of binding affinity using SPR

Binding affinity analysis of Z-M proteins with antibody

The binding constant (K_D) was determined using a CM5 chip that was amino-coupled using the His capture method. HBS-EP+ buffer (150 mM NaCl, 10 mM HEPES, 3 mM EDTA, and 0.05% (v/v) surfactant P20, pH 7.4) was used as the running buffer. The blank channel of the chip served as the negative control, while Z-M proteins were captured on the chip. Two-fold serial dilutions of antibodies at varying concentrations (1.56, 3.13, 6.25, 12.50, 25.00, and 50.00 nM) were flowed over the chip surface. After each cycle, the chip surface was regenerated using 10 mM glycine-HCl buffer (pH 1.5). The affinity was calculated using a 1:1 (Langmuir) binding fit model with BIA evaluation software.

Binding affinity analysis of HER2 with antibody or ADC

The binding constant (K_D) was determined using a CM5 chip that was amino-coupled via the Human Fc method. HBS-EP+ buffer (150 mM NaCl, 10 mM HEPES, 3 mM EDTA, and 0.05% (v/v) surfactant P20, pH 7.4) was used as the running buffer. The blank channel of the chip served as the negative control, while trastuzumab or trastuzumab-$MMAE_2$ was captured on the chip. Two-fold serial dilutions of antibodies at different concentrations (0.78, 1.56, 3.13, 6.25, and 12.50 nM) were flowed over the chip surface. The chip surface was regenerated with 3M magnesium chloride buffer after each cycle. The affinity was calculated using a 1:1 (Langmuir) binding fit model with BIA evaluation software.

Binding affinity analysis of FcRn with Antibody or ADC

To evaluate the binding kinetics, surface plasmon resonance (SPR) assays were conducted using the BIAcore T200 system. The binding constant (K_D) was determined using a CM5 chip that was amino-coupled through the directed immobilization method. A phosphate buffer (50 mM sodium phosphate, 150 mM NaCl, and 0.05% (v/v)

surfactant P20) at either pH 6.0 or pH 7.4 was used as the running buffer. The blank channel of the chip served as the negative control, while FcRn was immobilized on the chip. Two-fold serial dilutions of antibodies at varying concentrations (1.96, 3.91, 7.81, 15.63, and 31.25 nM) were flowed over the chip surface. After each cycle, the chip surface was regenerated using Tris-HCl buffer (0.1 M Tris-HCl, pH 8.0). The affinity was calculated using a 1:1 (Langmuir) binding fit model with BIA evaluation software.

2.5. Determination of cytotoxicity of the ADC product

Cell culture

Cells (MDA-MB-231, MDA-MB-453, and SK-BR-3) were cultured at RPMI1640, supplemented with 10% (v/v) FBS, 1% (v/v) penicillin/streptomycin. Cells were incubated at 37 °C in a humidified 5% CO_2 incubator.

Anti-proliferation assay

Cells were seeded in a 96-well plate with a density of 3,000 cells per well. After cellular attachment, a 4-fold serially diluted ADC in PBS was treated to the cells. After 72 h incubation at 37 °C, the cell viability was observed with CellTiter Glo. Fitted dose−response curves and GI50 values were obtained by GraphPad Prism 6.0 software. All experiments were conducted in duplicate.

Western blot

Cells (MDA-MB-231, MDA-MB-453, and SK-BR-3) were briefly washed by ice-cold PBS twice and lysed with a NP40 buffer (50 mM Tris-HCl pH 7.4, 1% NP40, 2 mM EDTA, 150 mM NaCl) containing protease inhibitor cocktail and phosphatase inhibitor cocktail. Each sample was loaded with an equal amount of protein and separated by SDS-PAGE gel. After transfer to a nitrocellulose membrane, it was blocked with 5% skim milk (in TBS/T). The membrane was incubated at 4 °C for overnight with uniformly diluted primary antibodies at 1:1000 (v/v) in TBS/T. After incubation with secondary antibodies (1:10000, v/v) for 1 h at room temperature, ECL solution was treated and chemiluminescence signals were detected by ImageQuant™ LAS 4000 (GE Healthcare). Western blot images were quantified with ImageJ (n = 3).

3. Organic synthesis

3.1. Synthesis of PyOx-Butyne

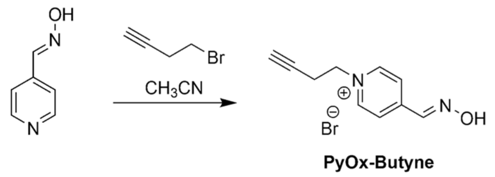

To a stirred solution of pyridine-4-carboxaldoxime (244 mg, 2.0 mmol) in dry CH_3CN (1.5 mL) was added 4-bromo-1-butyne (0.45 mL, 4.8 mmol), and the mixture was refluxed for 24 hours. The solution was cooled to room temperature, and the precipitate was collected by filtering to afford **PyOx-Butyne** (367 mg, 1.4 mmol, 72%) as a yellow solid.

^{1}H NMR (400 MHz, D_2O) δ 8.86 (d, *J* = 6.4 Hz, 2H), 8.24 (s, 1H), 8.15 (d, *J* = 6.4 Hz, 2H), 4.64 (t, *J* = 6.4 Hz, 2H), 2.88 (m, 2H), 2.48 (m, 1H).

HRMS: Exact mass calculated for $[C_{10}H_{11}N_2O_1+H]^+$ requires *m*/*z* = 175.0866, found *m*/*z* = 175.0868 (HESI+).

3.2. Synthesis of PyOx-Hexyne

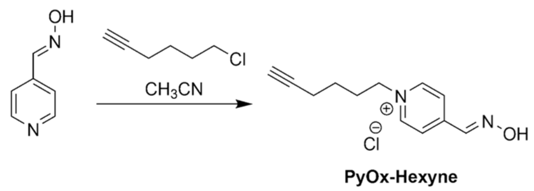

PyOx-Hexyne (288 mg, 1.20 mmol, 42%) was prepared by the same procedure used for **PyOx-Butyne** starting from pyridine-4-carboxaldoxime (350 mg, 2.87 mmol) and 6-chloro-1-hexyne (1.04 ml, 8.61 mmol).

^{1}H NMR (400 MHz, D_2O) δ 8.80 (d, *J* = 6.4 Hz, 2H), 8.34 (s, 1H), 8.16 (d, *J* = 6.4 Hz, 2H), 4.59 (t, *J* = 7.6 Hz, 2H), 2.32 (m, 1H), 2.25 (m, 2H), 2.11 (m, 2H), 1.55 (m, 2H).

HRMS: Exact mass calculated for $[C_{12}H_{15}N_2O_1+H]^+$ requires *m*/*z* = 203.1179, found *m*/*z* = 203.1182 (HESI+).

3.3. Synthesis of *tert*-butyl (15-((4-nitrophenyl)sulfonamido)-15-oxo-3,6,9,12-tetraoxapentadecyl) carbamate (S1)

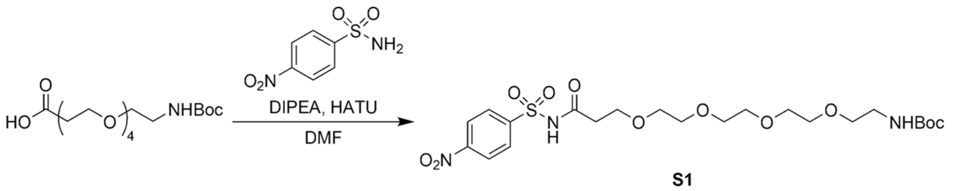

S1

To a solution of Boc-NH-PEG4-COOH (1.00 g, 2.74 mmol, 1.2 equiv) in dry DMF (10 mL), were added diisopropylethylamine (DIPEA, 1.05 mL, 6.02 mmol), 4-nitrobenzenesulfonamide (0.61 g, 3.01 mmol, 1 equiv), and 1-[bis(dimethylamino)methylene]-1H-1,2,3-triazolo[4,5-b]pyridinium 3-oxide hexafluorophosphate (HATU, 1.25 g, 3.28 mmol), and the mixture was allowed to stir at room temperature for 6 hours. The resulting mixture was diluted with ethyl acetate and washed twice with 0.5 M aqueous HCl and brine. The organic phase was dried over $MgSO_4$ and evaporated under reduced pressure. The residue was purified by flash column chromatography on silica gel (CH_2Cl_2:MeOH = 20:1) to give **S1** (0.83 g, 55% yield) as a colorless oil.

^{1}H NMR (400 MHz, $CDCl_3$) δ 8.32 (d, *J* = 8.8 Hz, 2H), 8.25 (d, *J* = 8.8 Hz, 2H), 3.71–3.51 (m, 18H), 3.32 (m, 2H), 2.56 (m, 2H), 1.42 (s, 9H).

^{13}C NMR (100 MHz, $CDCl_3$) δ 156.4, 150.3, 129.7 (2C), 123.9 (2C), 70.69, 70.62, 70.58, 70.5, 70.35, 70.32, 70.2, 70.11, 70.06, 66.7, 53.6, 40.4, 37.8, 28.5 (3C).

3.4. Synthesis of *tert*-butyl (1-(4-nitrophenyl)-2-((4-nitrophenyl)sulfonyl)-3-oxo-6,9,12,15-tetraoxa-2-azaheptadecan-17-yl)carbamate (S2)

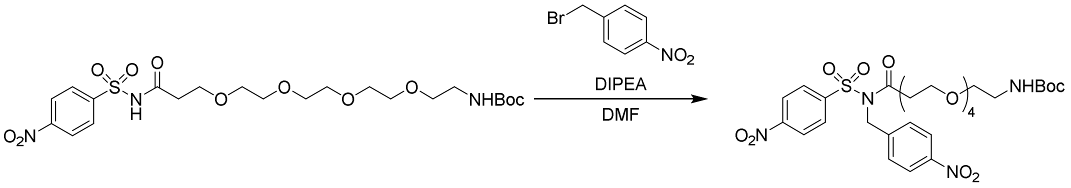

S1 S2

To a solution of compound **S1** (1.0 g, 1.8 mmol, 1 equiv) in dry DMF (6.3 mL, 0.3 M) was added 4-nitrobenzylbromide (472 mg, 2.2 mmol, 1.2 equiv) and DIPEA (0.6 mL, 3.6 mmol, 2 equiv). The mixture was allowed to stir at room temperature for 24 hours. After the removal of the solvent, the reaction mixture was diluted with EtOAc and washed with brine. The organic phase was dried over $MgSO_4$ and evaporated under reduced pressure. The residue was purified by flash column chromatography on silica gel (Hexanes:EtOAc = 1:2) to give compound **S2** (731 mg, 59% yield) as a colorless oil.

^{1}H NMR (400 MHz, $CDCl_3$) δ 8.39 (d, *J* = 9.0 Hz, 2H), 8.24 (d, *J* = 8.8 Hz, 2H), 8.13 (d, *J* = 8.9 Hz, 2H), 7.56 (d, *J* = 8.8 Hz, 2H), 5.20 (s, 2H), 3.70–3.65 (m, 2H), 3.64–3.57 (m, 8H), 3.56–3.52 (m, 2H), 3.52–3.48 (m, 4H), 3.34–3.23 (m, 2H), 2.82 (t, *J* = 6.0 Hz, 2H), 1.43 (s, 9H).

^{13}**C NMR** (175 MHz, $CDCl_3$) δ 171.5, 151, 147.9, 144.7, 143.4, 129.5 (2C), 128.4 (2C), 124.6 (2C), 124.2 (2C), 70.8, 70.7 (2C), 70.5, 70.39, 70.37, 66.5, 49.5, 40.5, 37.0, 31.7, 28.6 (3C), 22.8, 14.2.

3.5. Synthesis of NASA-FL

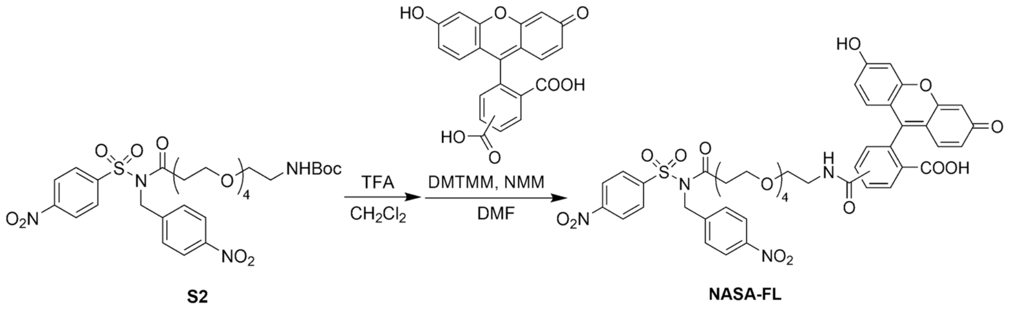

To a solution of compound **S2** (8.2 mg, 12.0 μmol) in CH_2Cl_2 (0.5 mL) was added TFA (30 μl). The mixture was stirred at room temperature for 1 hour and concentrated in vacuo. The residue was dissolved in dry DMF (0.5 mL) and 5(6)-carboxyfluorescein (5.4 mg, 14.4 μmol, 1.2 equiv), DMTMM (3.9 mg, 14.1 μmol, 1.2 equiv) and *N*-methylmorpholine (NMM) (3.9 μL, 35.5 μmol, 3 equiv) were added. The mixture was stirred at room temperature for 1 hour and concentrated. The residue was purified by flash column chromatography on silica gel (CH_2Cl_2:MeOH = 10:1) to give **NASA-FL** (6.1 mg, 54% yield).

^{1}**H NMR** (400 MHz, CD_3OD) δ 8.39 (d, J = 8.4 Hz, 2H), 8.20–8.10 (m, 6H), 7.61–7.22 (m, 3H), 6.66–6.50 (m, 6H), 5.25 (s, 2H), 3.63–3.33 (m, 18H), 2.79–2.75 (m, 2H).

HRMS: Exact mass calculated for $[C_{45}H_{42}N_4O_{17}S+H]^+$ requires m/z = 943.2338, found m/z = 943.2339 (HESI+).

3.6. Synthesis of NASA-Bt

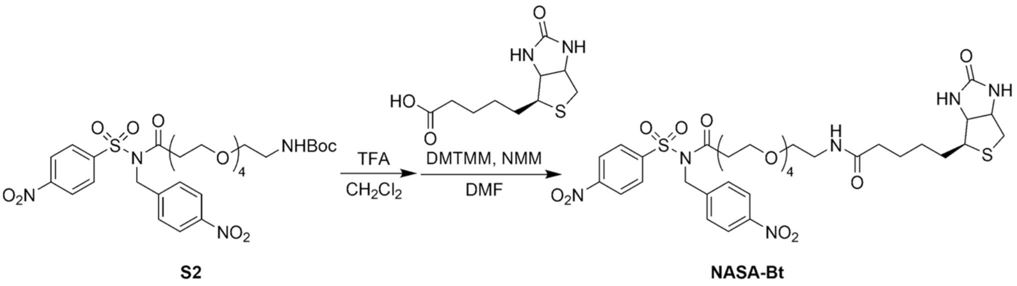

NASA-BT (6.8 mg, 55% yield) was prepared by the same procedure used for **NASA-FL**.

^{1}**H NMR** (400 MHz, CD_3OD) δ 8.34 (d, J = 8.4 Hz, 2H), 8.15 (m, 4H), 8.11 (d, J = 8.9 Hz, 2H), 7.53 (d, J = 8.8 Hz, 2H), 5.20 (s, 2H), 4.38 (m, 1H), 4.19 (m, 1H), 3.56–3.38 (m, 18H), 3.09 (m, 1H), 2.76 (m, 2H), 2.59 (dd, J = 12.8 Hz, 1H), 2.10 (m, 2H), 1.55–1.32 (m, 6H).

HRMS: Exact mass calculated for $[C_{34}H_{46}N_6O_{13}S_2+Na]^+$ requires *m*/*z* = 833.2457, found *m*/*z* = 833.2490 (HESI+).

3.7. Synthesis of NASA-N_3

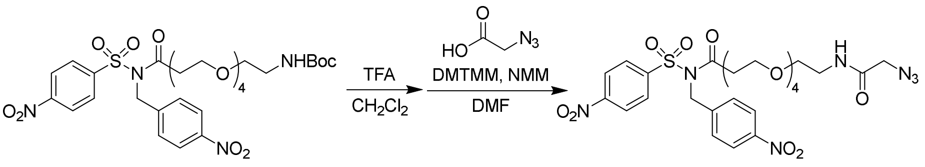

S2 **NASA-N_3**

To a solution of compound **S2** (100 mg, 0.15 mmol, 1 equiv) in CH_2Cl_2 (7 mL, 0.02 M) was added TFA (0.02 mL, 0.30 mmol, 2 equiv). The mixture was allowed to stir at room temperature for 1 hour, and the solvent was removed in vacuo. To a solution of this residue in dry DMF (7 mL, 0.02 M) was added 2-azidoacetic acid (18 mg, 0.18 mmol, 1.2 equiv), DMTMM (49 mg, 0.18 mmol, 1.2 equiv) and *N*-methylmorpholine (NMM) (0.05 mL, 0.35 mmol, 2.3 equiv). The mixture was allowed to stir at room temperature for 8 hours. After removal of the solvent, the resulting mixture was purified by flash column chromatography on silica gel (CH_2Cl_2:MeOH = 20:1) to give compound **NASA-N_3** (83 mg, 86% yield) as a colorless oil.

^{1}H NMR (400 MHz, $CDCl_3$) δ 8.37 (d, *J* = 9.0 Hz, 2H), 8.20 (d, *J* = 8.8 Hz, 2H), 8.11 (d, *J* = 8.9 Hz, 2H), 7.54 (d, *J* = 8.7 Hz, 2H), 6.94 (s, 1H), 5.18 (s, 2H), 3.91 (s, 2H), 3.66 (t, *J* = 6.1 Hz, 2H), 3.63–3.57 (m, 8H), 3.56–3.46 (m, 6H), 3.43 (m, 2H), 2.81 (t, *J* = 6.1 Hz, 2H).

^{13}C NMR (175 MHz, $CDCl_3$) δ 171.5, 166.9, 150.9, 147.9, 144.7, 143.4, 129.5 (2C), 128.4 (2C), 124.6 (2C), 124.2 (2C), 70.70, 70.66 (3C), 70.5, 70.4, 69.7, 66.5, 52.8, 49.5, 39.3, 37.0.

IR (FT-ATR, cm^{-1}, CH_2Cl_2) ν_{max} 3064, 2873, 2359, 2338, 2106, 1837, 1818, 1779, 1756, 1707, 1689, 1669, 1647, 1641, 1629, 1623, 1606, 1576, 1570, 1559, 1526, 1496, 1481, 1465, 1442, 1424, 1404, 1346, 1267, 1168, 1086, 1012, 854, 731, 700, 682.

HRMS: Exact mass calculated for $[C_{26}H_{33}N_7O_{12}S+H]^+$ requires *m*/*z* = 668.1981, found *m*/*z* = 668.1980 (FAB+).

3.8. Synthesis of Fmoc-VC-PABC-MMAE

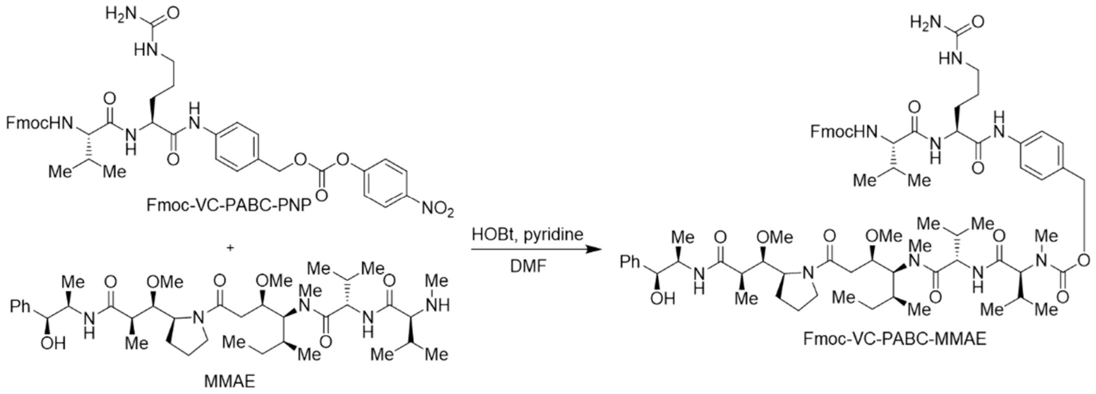

Fmoc-VC-PABC-PNP (200 mg, 0.26 mmol, 1 equiv) and MMAE (280 mg, 0.39 mmol, 1.5 equiv) were dissolved in DMF (40 mL, 6.5 mM). Then, hydroxybenzotriazole (HOBt, 8 mg, 0.52 mmol, 2 equiv) and pyridine (0.6 mL, 7.5 mmol, 28 equiv) were added to the solution. The reaction was stirred at room temperature for 30 hours and monitored by RP-HPLC. Finally, the product was purified with a preparative column to give a white powder (232 mg, 66% yield).

3.9. Synthesis of NH_2-VC-PABC-MMAE

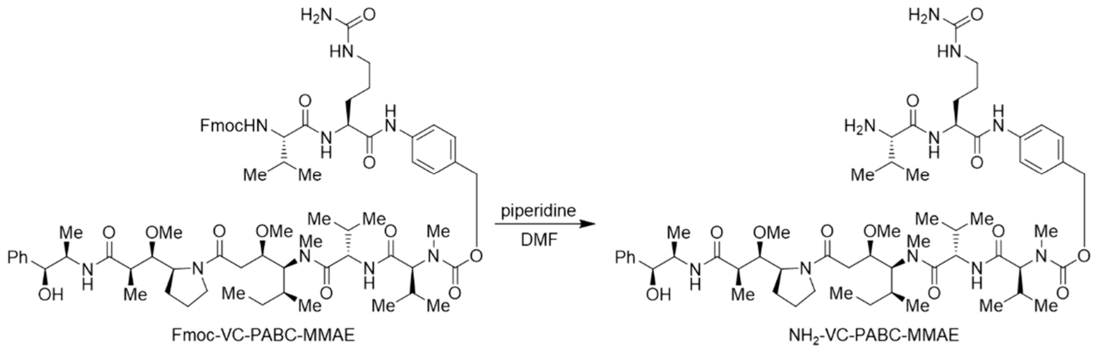

Fmoc-VC-PABC-MMAE (125 mg, 0.09 mmol) was dissolved in DMF (0.9 mL, 0.1 M) containing 20.0% piperidine and the reaction was stirred at room temperature. After 1 hour, the RPLC analysis indicated the completion of de-Fmoc process. Then the solution was concentrated under reduced pressure. The product was combined to give a white powder (88.0 mg, 88% yield).

3.10. Synthesis of BCN-VC-PABC-MMAE

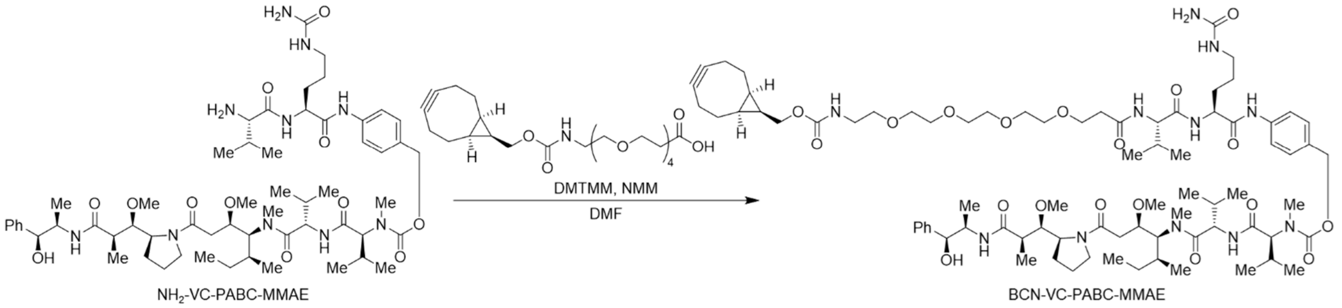

BCN-PEG$_4$-COOH (13 mg, 0.03 mmol, 1 equiv), NH$_2$-VC-PABC-MMAE (40 mg, 0.036 mmol, 1.2 equiv), DMTMM (10 mg, 0.036 mmol, 1.2 equiv) and *N*-methylmorpholine (NMM) (0.01 mL, 0.09 mmol, 3 equiv) were dissolved in DMF (0.14 mL, 0.2 M). The mixture was allowed to stir at room temperature for 8 hours. After removal of the solvent, the resulting mixture was purified with a preparative column to give a white powder (22 mg, 48% yield).

HRMS: Exact mass calculated for $[C_{80}H_{127}N_{11}O_{19}+Na]^+$ requires *m*/*z* = 1568.9202, found *m*/*z* = 1568.9223 (HESI+).

4. Supplementary figures and tables

4.1. Supplementary figures

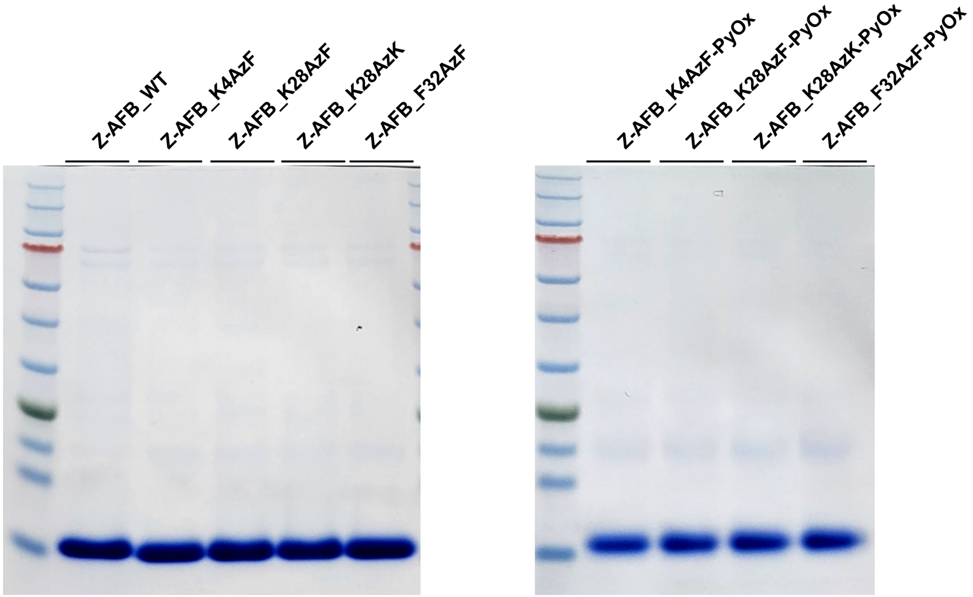

Figure S1. SDS-PAGE images of Z-AFB_WT, Z-AFB_ncAA, and Z-AFB_ncAA-PyOx.

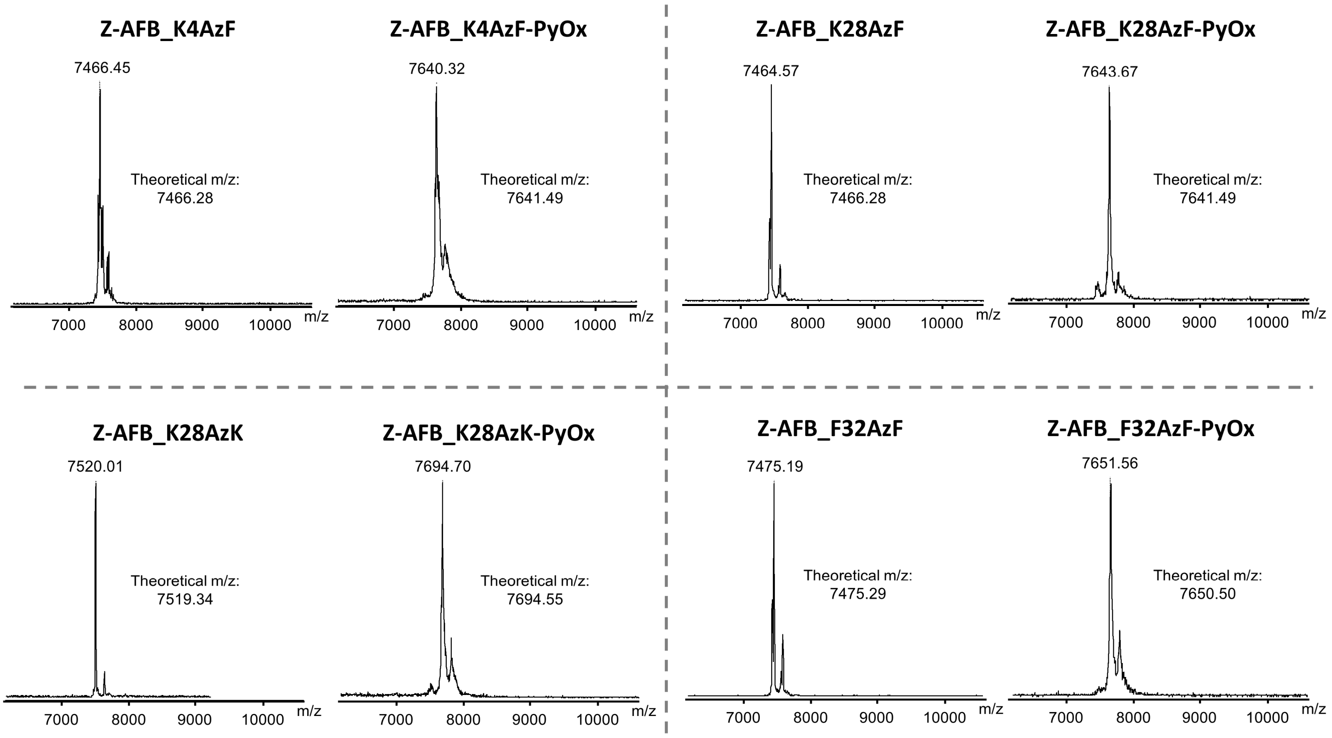

Figure S2. MALDI-TOF MS data of Z-AFB_ncAA, and Z-AFB_ncAA-PyOx. For Z-AFB_F32AzF, K28 was mutated to Arg to prevent the self-labeling of the probes.

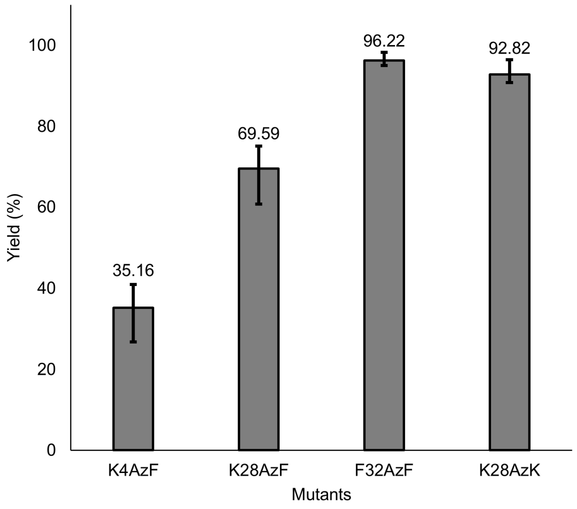

	Affibody K4	Affibody K28	Affibody F32
Distance to the target Lys (Å)	20.8	11.6	7.8

Figure S3. The labeling yields of intact Z-DM using each Z-AFB-PyOX variant. Reaction conditions: 50 mM HEPES pH 7.2, Z-DM (15 µM), Z-AFB-PyOx (30 µM), NASA-FL (75 µM), 37 °C, and 4 hours.

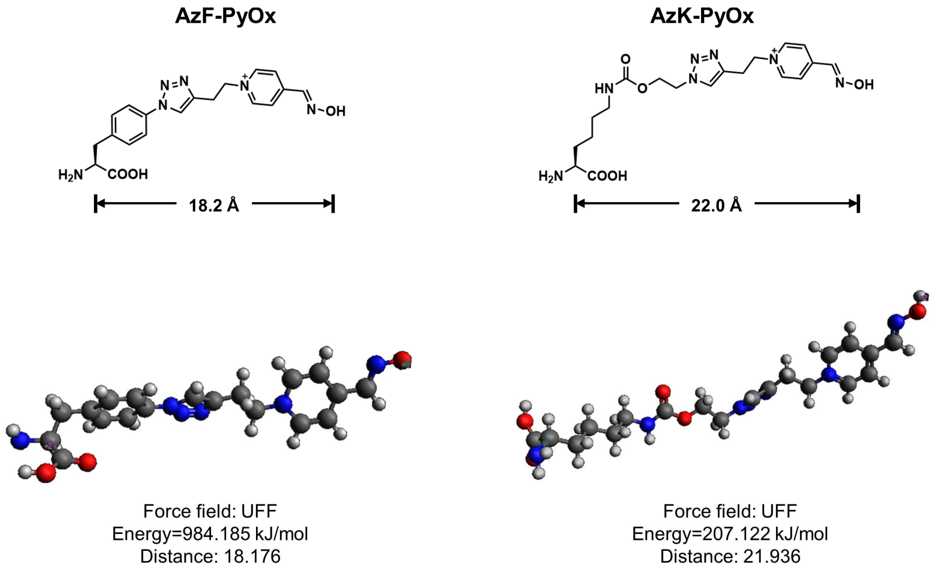

Figure S4. The molecular length analysis of AzF-PyOx and AzK-PyOx using Avogadro software.[6]

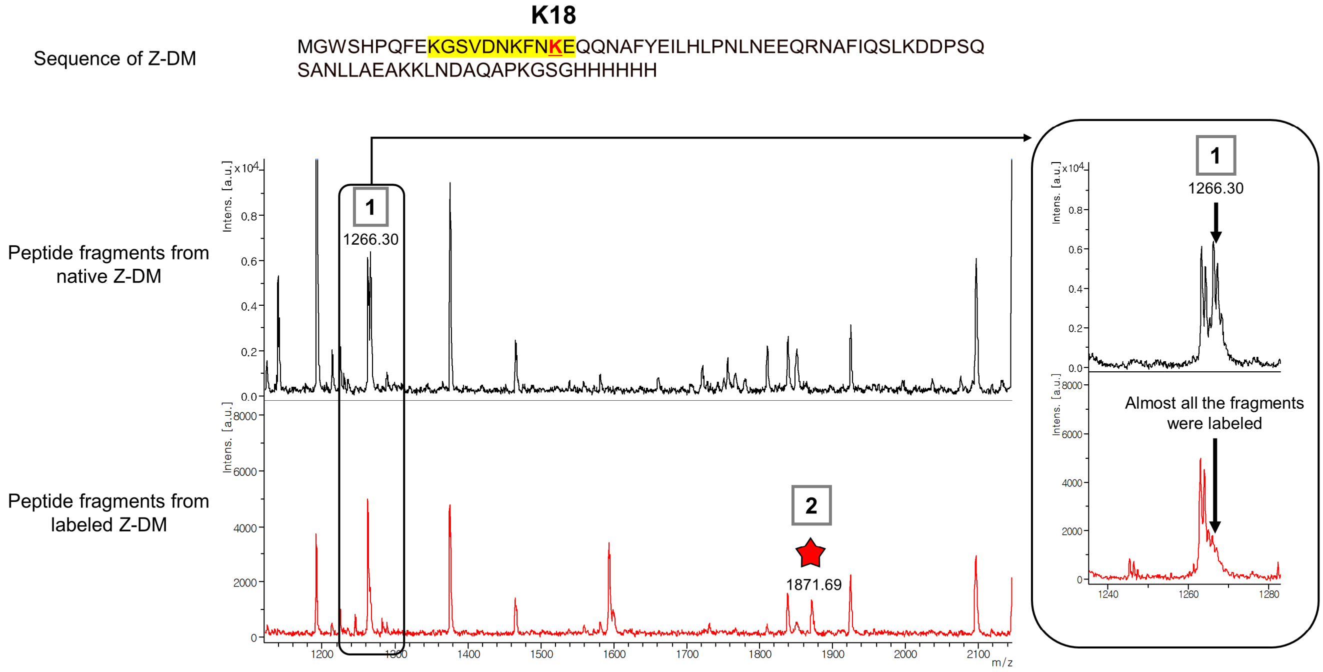

Figure S5. Protease (Glu-C) digestion analysis of native Z-DM and FL-labeled Z-DM. The peptide fragment 1 contains the expected target lysine (K18) and was shifted to show the mass of FL-conjugated form (peptide fragment 2).

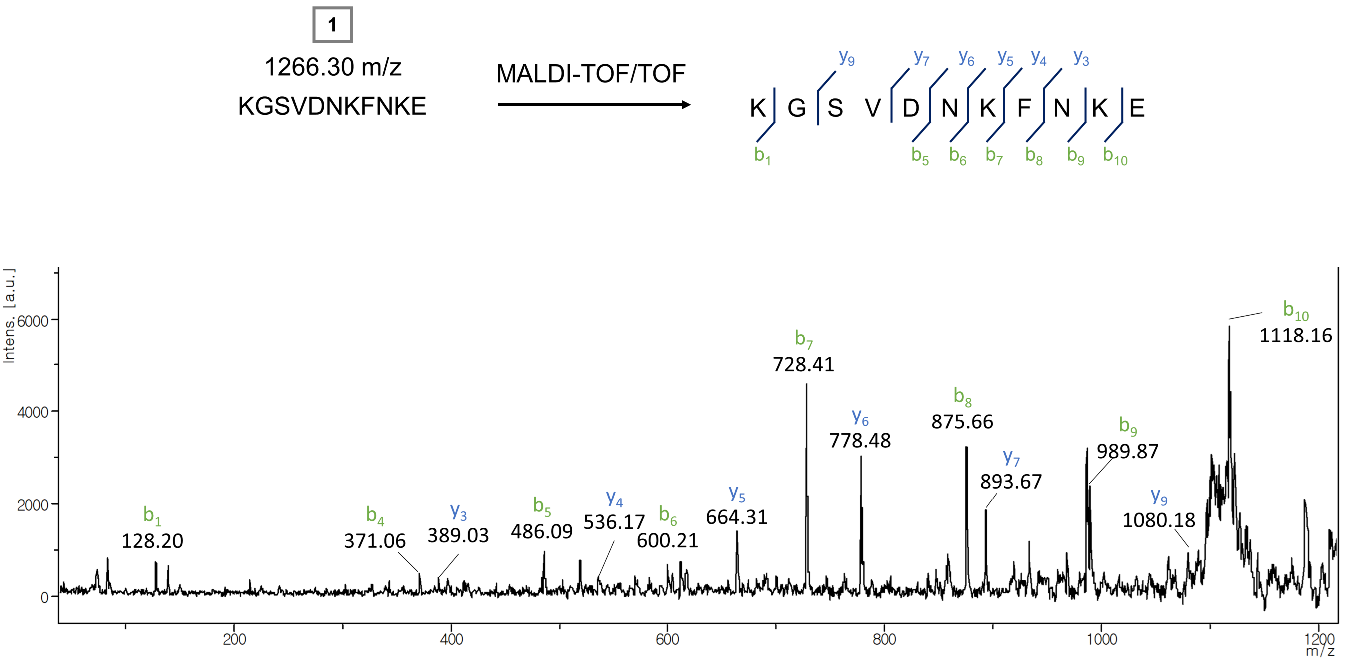

Figure S6. MALDI-TOF/TOF tandem MS analysis of the peptide fragment 1 in Figure S5 (the fragment from unlabeled Z-DM).

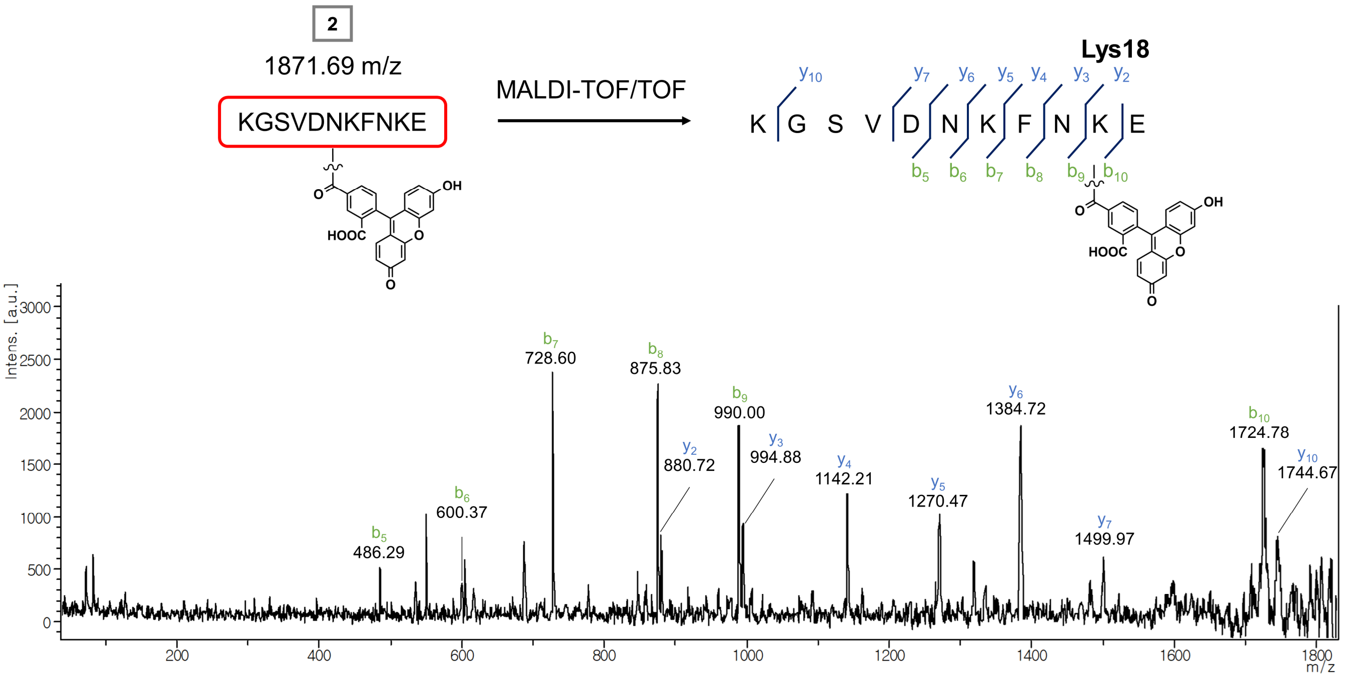

Figure S7. MALDI-TOF/TOF tandem MS analysis of the peptide fragment 2 in Figure S5 (the fragment from labeled Z-DM), demonstrating that K18 was labeled as expected (The spectrum shows the extended mass range of Figure 2D and was included for comparison with the peptide fragment 1).

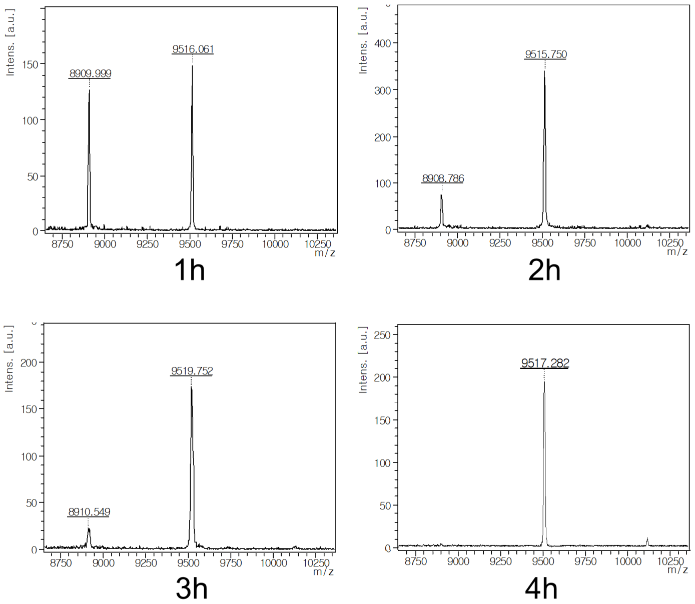

Figure S8. MALDI-TOF MS data for labeling of Z-DM was performed using Z-AFB_F32AzF-PyOx under different time periods. Increasing the reaction time resulted in higher yields. Reaction conditions: 50 mM HEPES pH 7.2, Z-DM (15 μM), Z-AFB-PyOx (30 μM), NASA-FL (75 μM), 37 °C, and 1 to 4 hours.

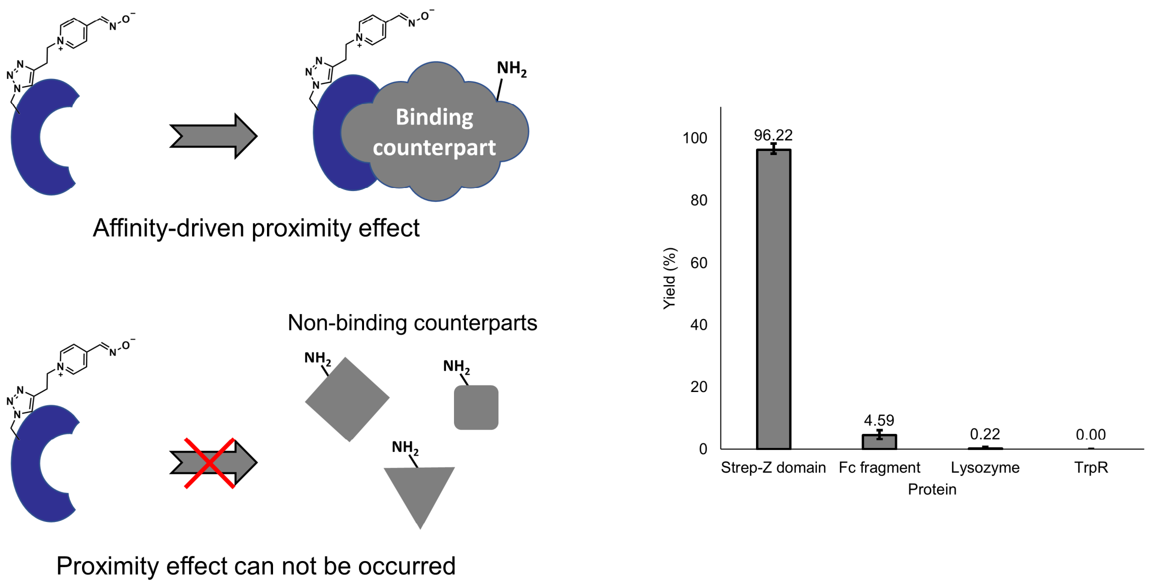

Figure S9. Labeling assessment of non-binding counterparts. Only no or little (< 5%) labeling was observed from non-binding proteins. Reaction conditions: 50 mM HEPES pH 7.2, each protein (15 μM), Z-AFB-PyOx (30 μM), NASA-FL (75 μM), 37 °C, and 4 hours.

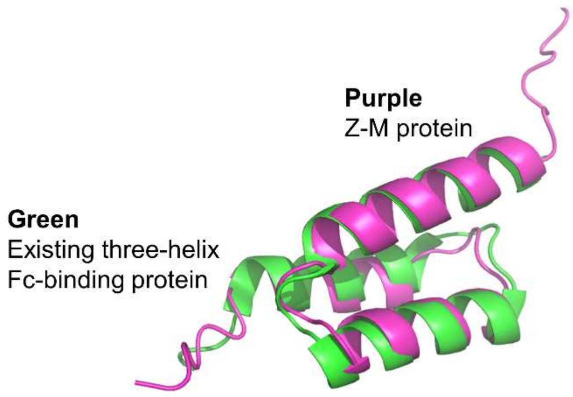

Figure S10. A superimposed image of crystal structures of the Z-M protein constructed by AlphaFold2 and a three-helix Fc-binding protein derived from PDB ID 5U4Y.[7]

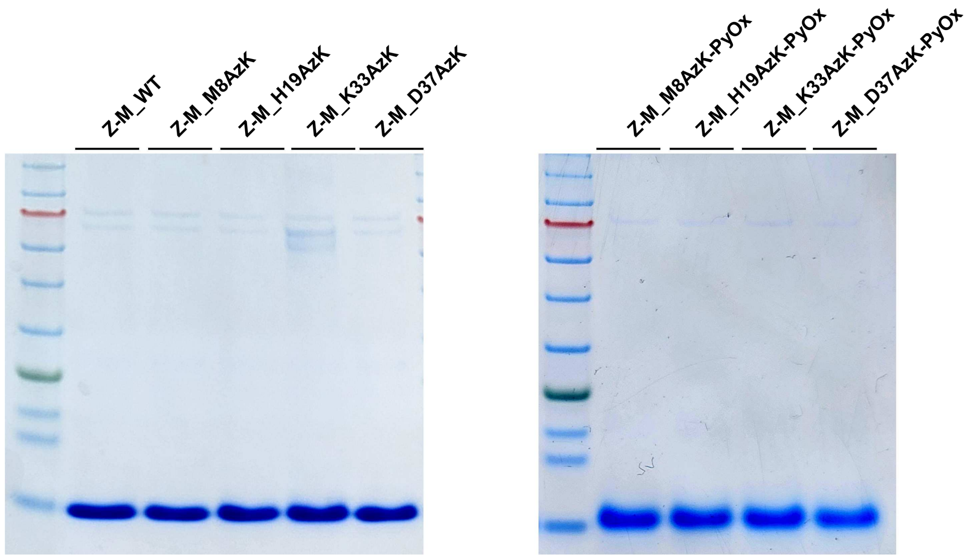

Figure S11. SDS-PAGE images of Z-M_WT, Z-M_ncAA, and Z-M_ncAA-PyOx.

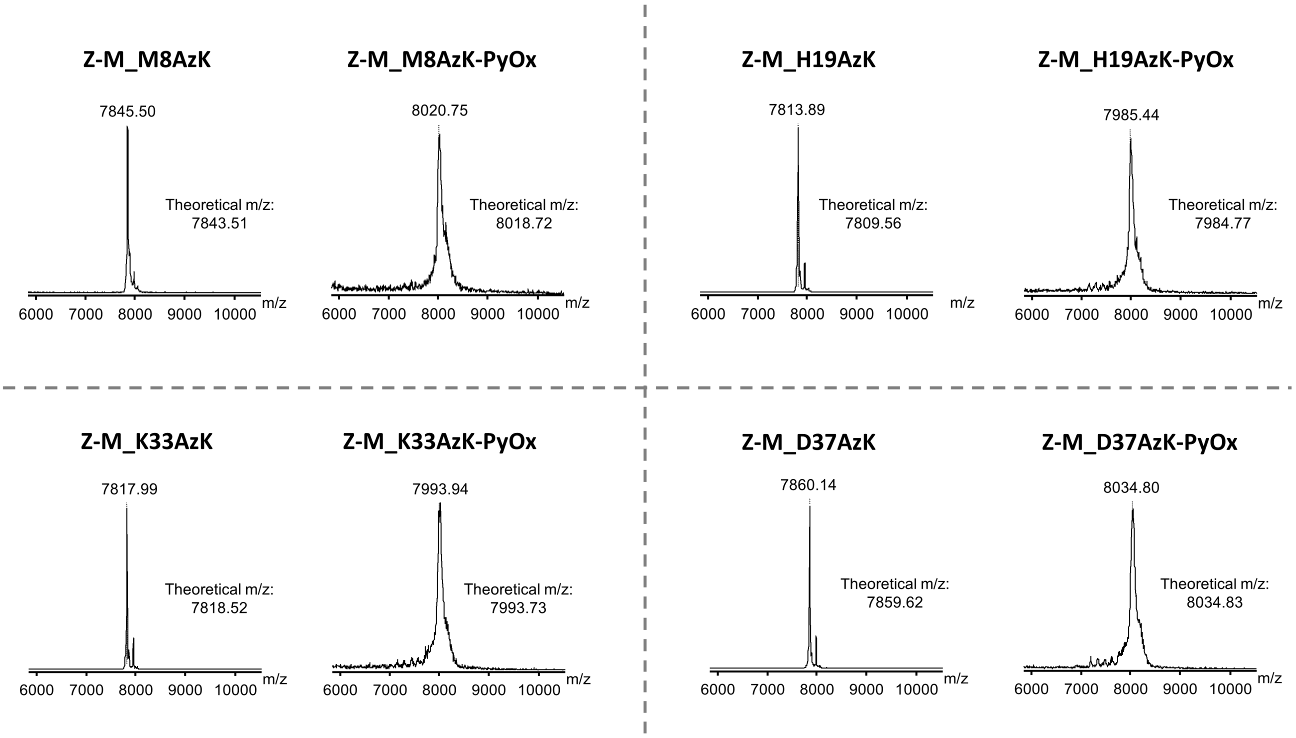

Figure S12. MALDI-TOF MS analysis of Z-M_ncAA, and Z-M_ncAA-PyOx. For Z-M_M8AzK and Z-M_D37AzK, K5 and K33, respectively, were mutated to Arg to prevent the self-labeling of the probes.

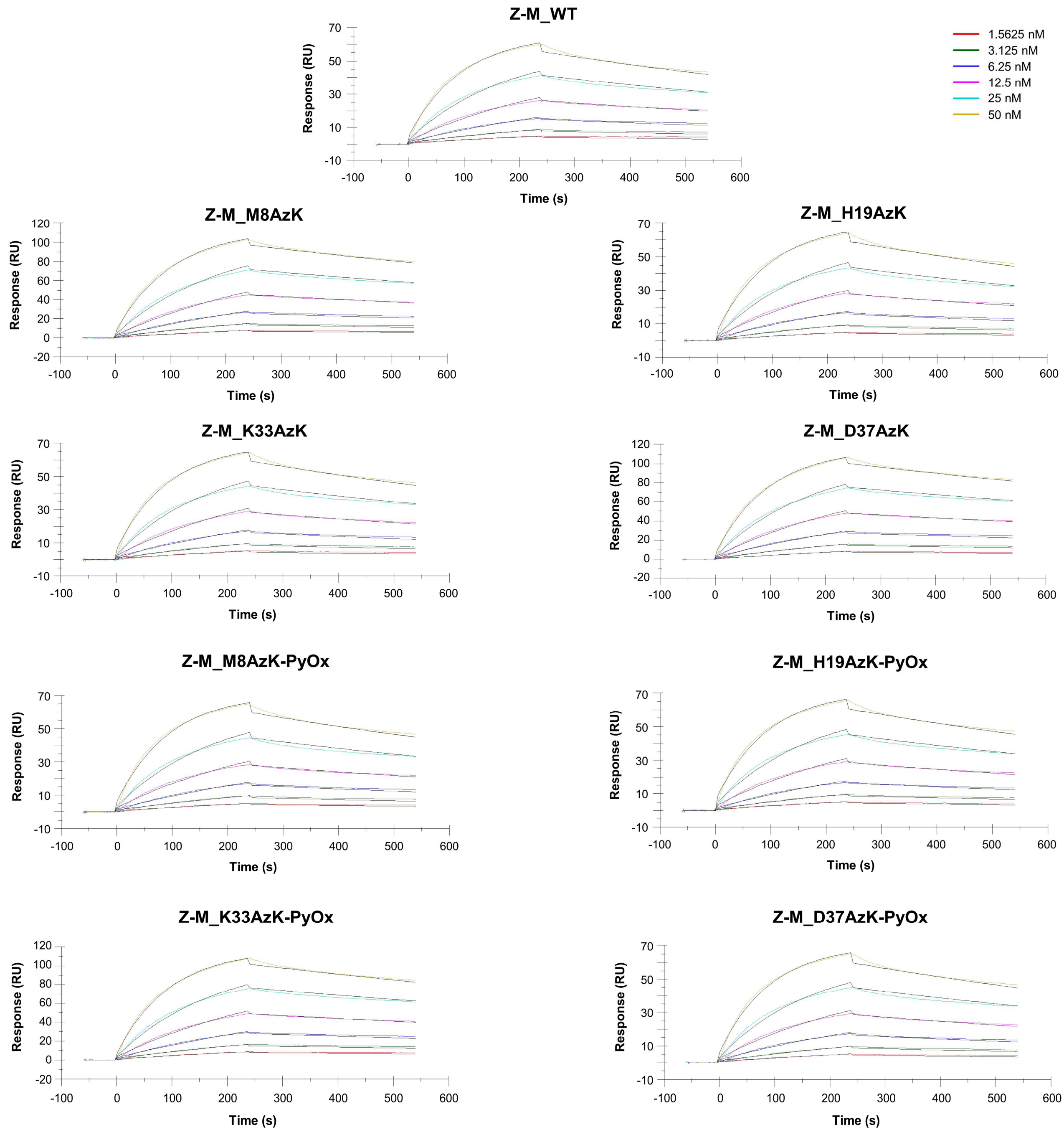

Figure S13. Binding assay of Z-M_WT, M8AzK, H19AzK, K33AzK, D37AzK, M8AzK-PyOx, H19AzK-PyOx, K33AzK-PyOx, and D37AzK-PyOx against trastuzumab WT using SPR.

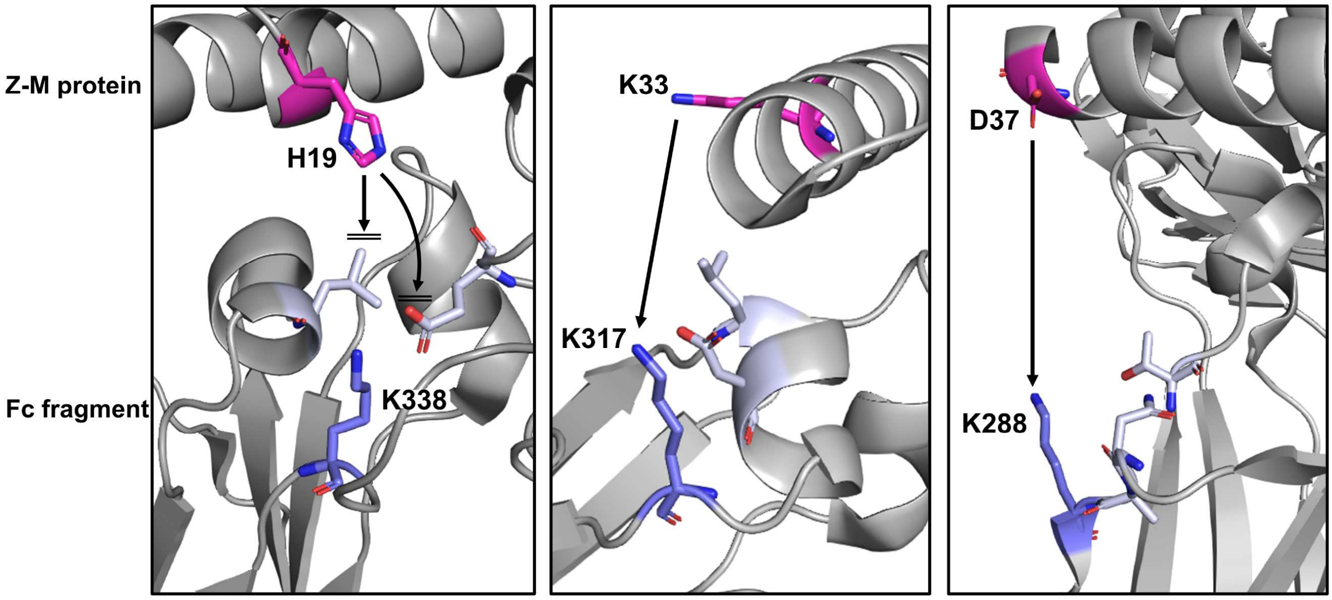

Figure S14. Comparison of structural hindrance regarding the target lysines for H19, K33, and D37. In the case of K33 and D37, the target lysines remain unobstructed by neighboring amino acids, facilitating direct contact between PyOx and the lysines. In contrast, reaching the target lysine from H19 presents greater difficulty due to structural hindrance from nearby residues.

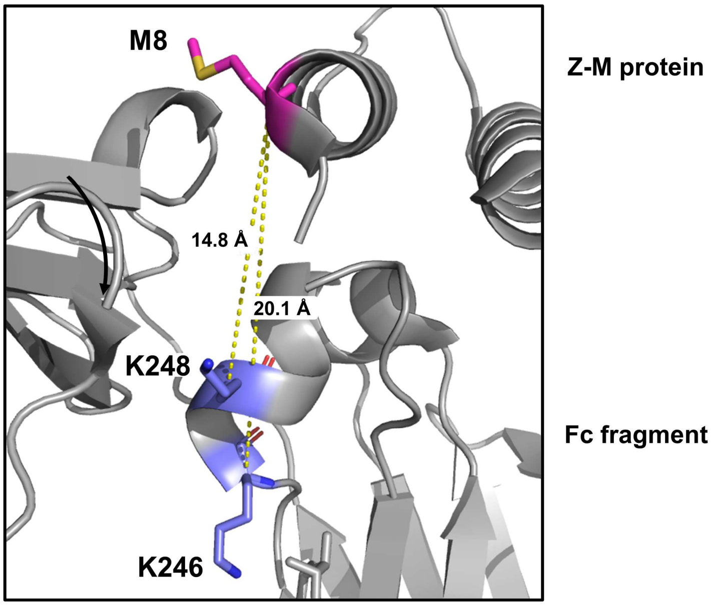

Figure S15. Comparison of the distances between M8 and K248/K246.

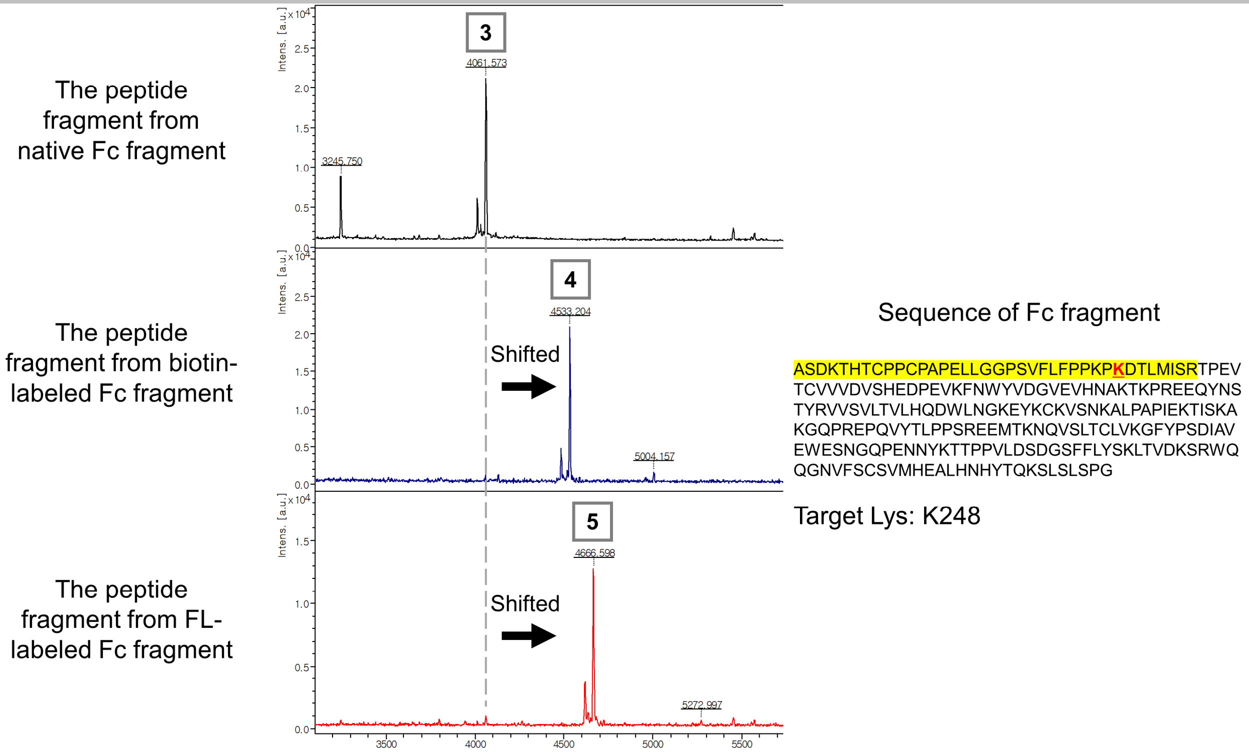

Figure S16. Protease (Trypsin) digestion analysis of WT, FL-labeled, and Bt-labeled Fc fragment. The peptide fragment 3 contains the expected target lysine (K248) and was shifted to show the mass of probe-conjugated forms (peptide fragment 4 and 5).

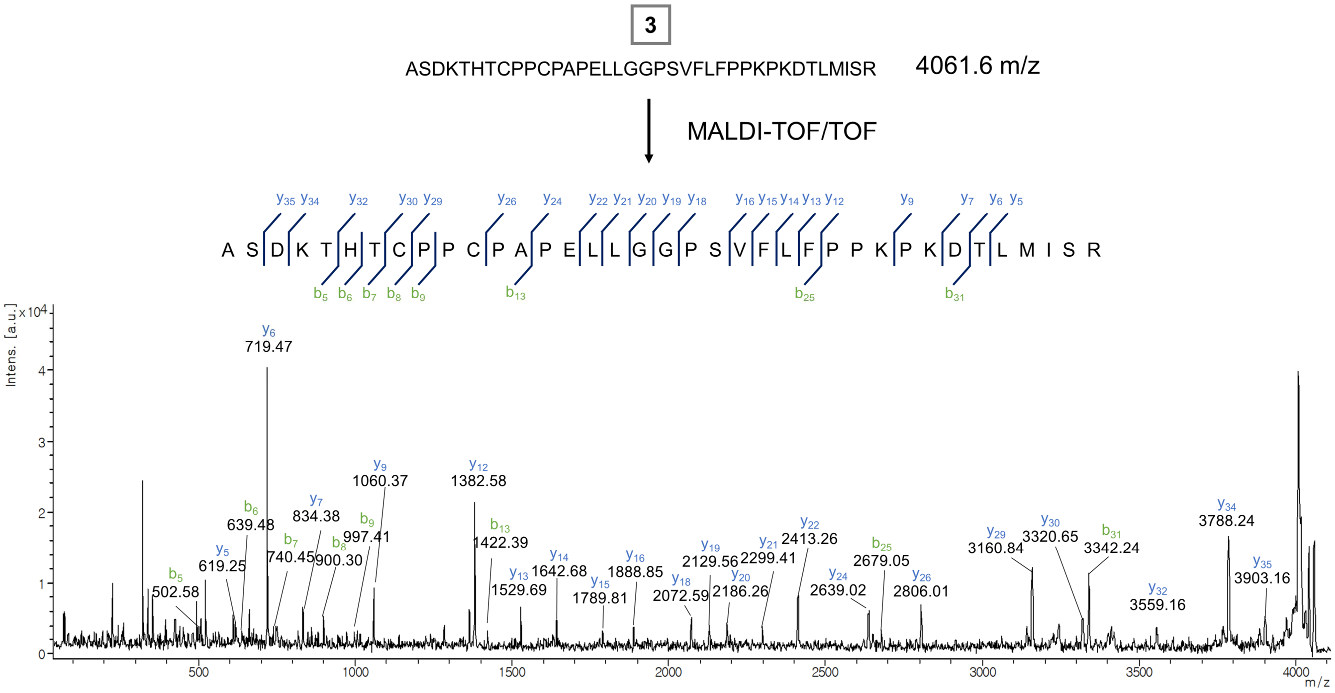

Figure S17. MALDI-TOF/TOF tandem MS analysis of the peptide fragment 3.

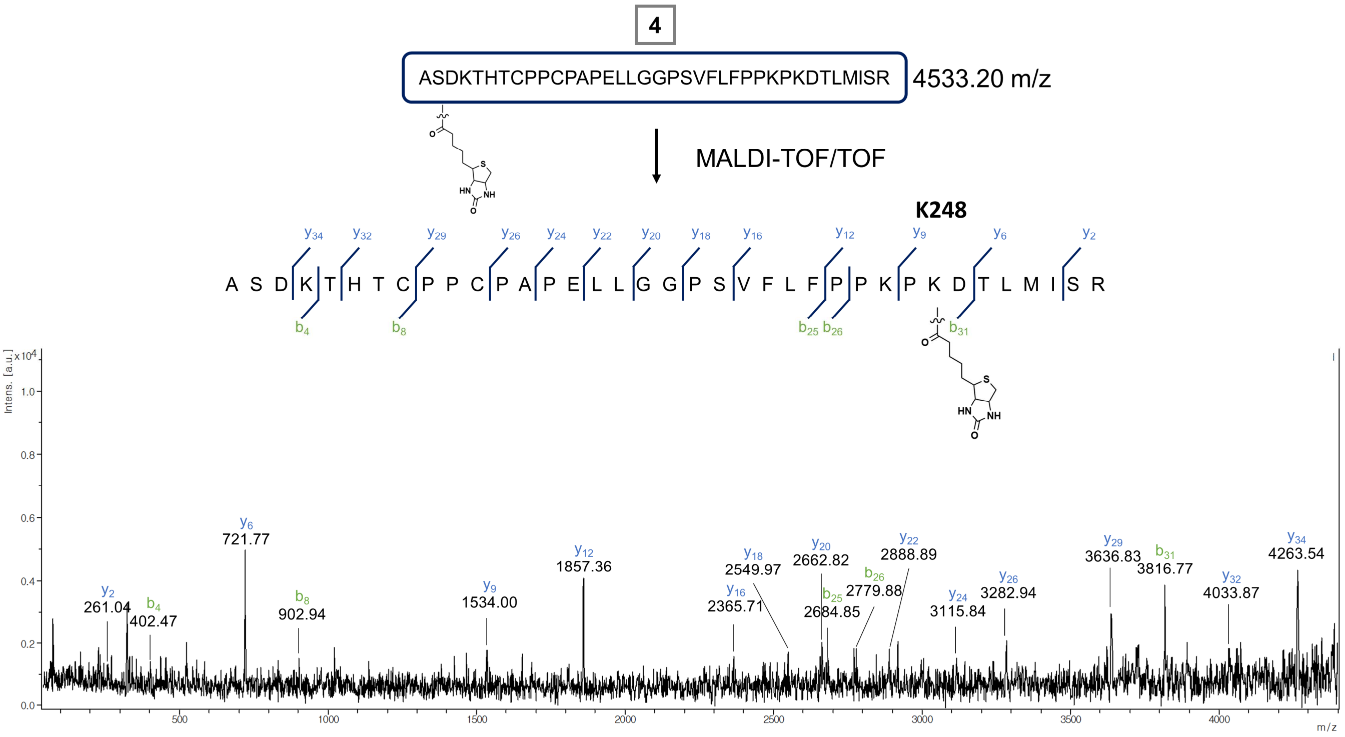

Figure S18. MALDI-TOF/TOF tandem MS analysis of the peptide fragment 4, demonstrating the labeling of biotin at K248.

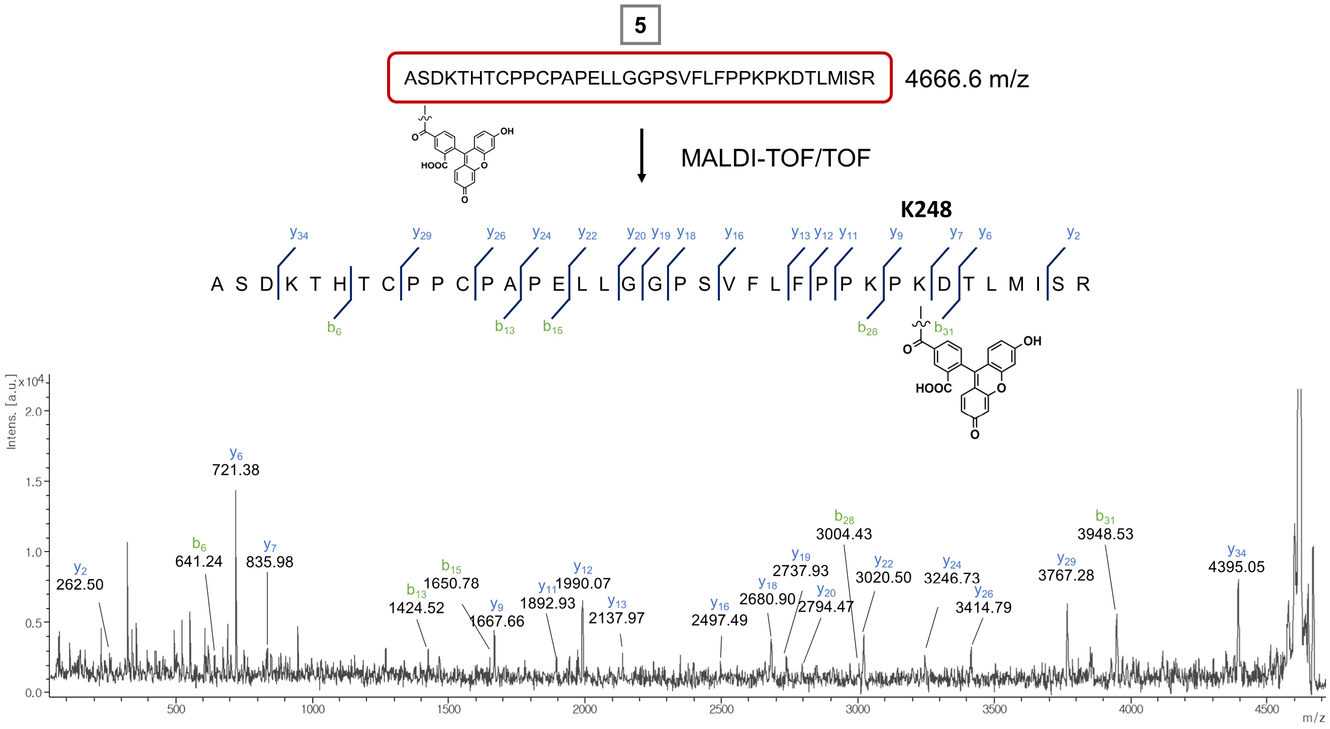

Figure S19. MALDI-TOF/TOF tandem MS analysis of the peptide fragment 5, demonstrating the labeling of FL at K248. (The spectrum shows the extended mass range Figure 3D and was included for comparison with the peptide fragment 3).

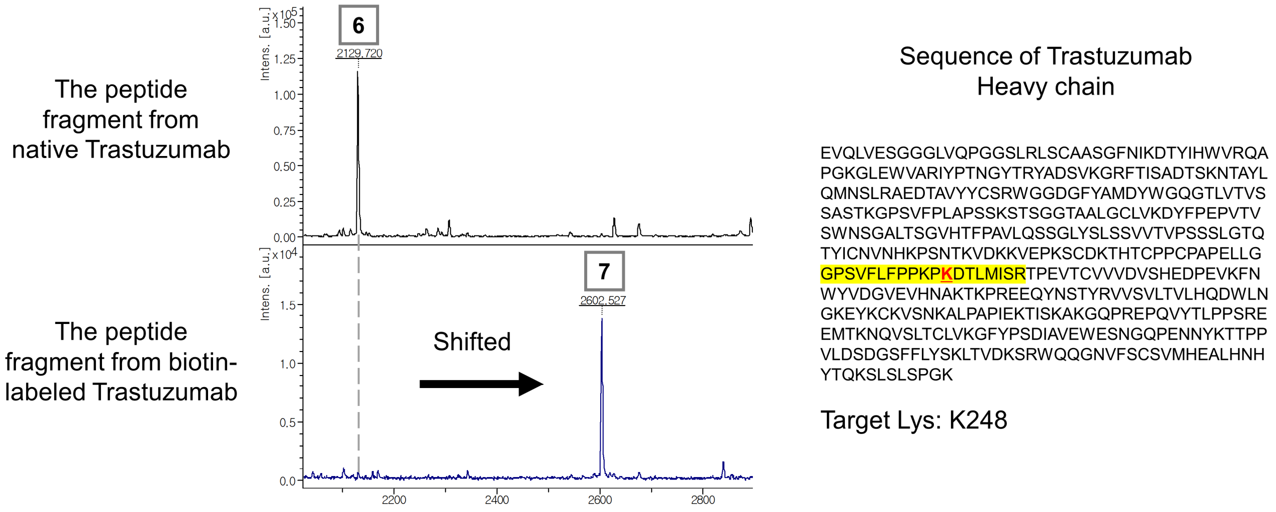

Figure S20. Protease (Trypsin) digestion analysis of WT and Bt-labeled trastuzumab. The peptide fragment 6 contains the expected target lysine (K248) and was shifted to show the mass of Bt-conjugated form (peptide fragment 7).

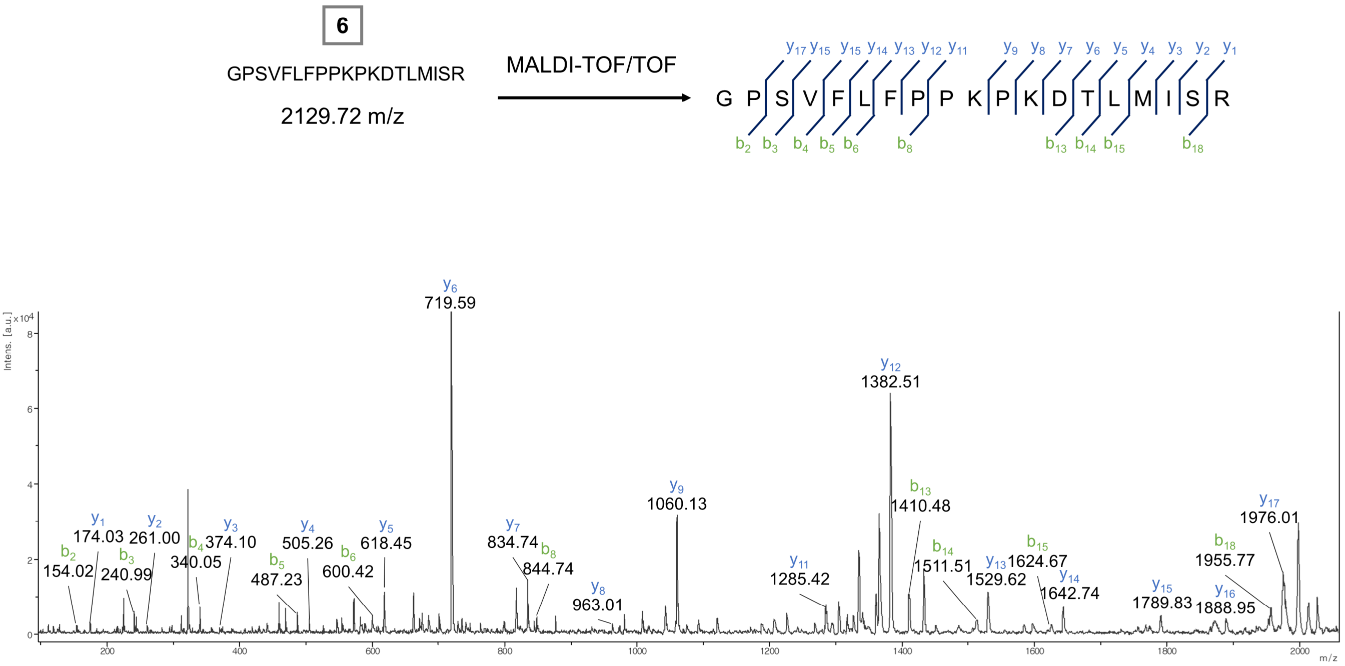

Figure S21. MALDI-TOF/TOF tandem MS analysis of the peptide fragment 6.

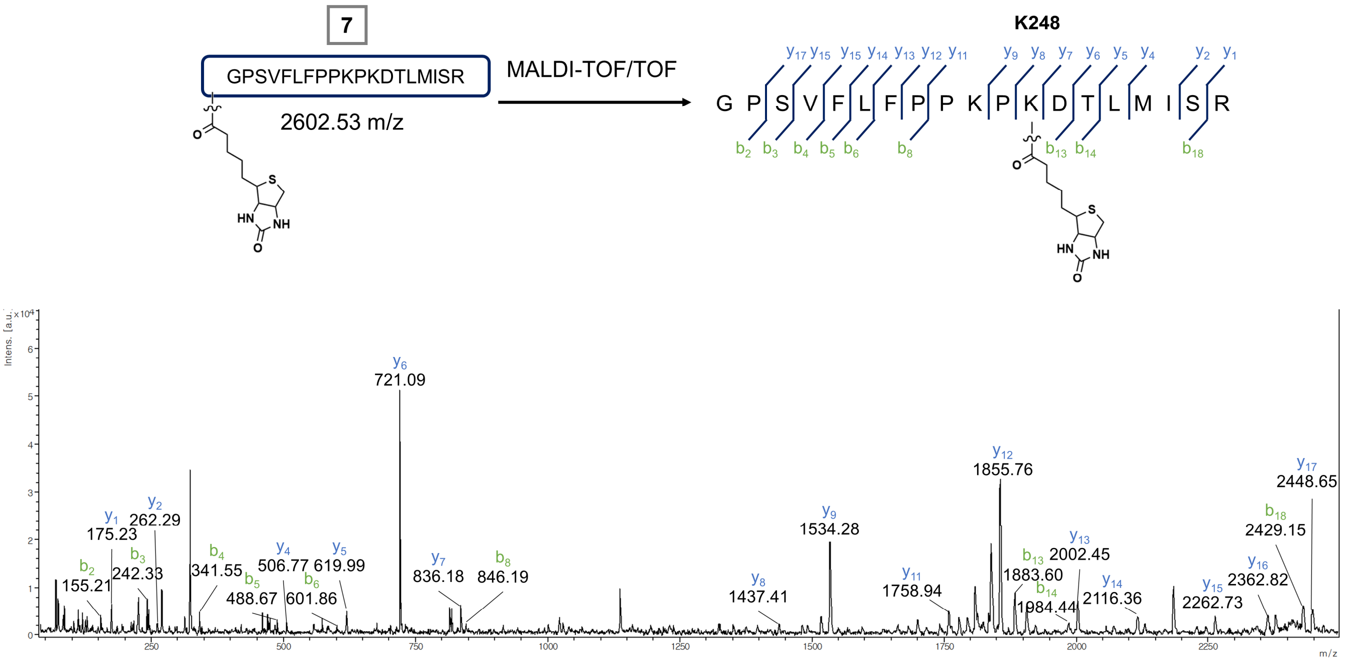

Figure S22. MALDI-TOF/TOF tandem MS analysis of the peptide fragment 7, demonstrating the labeling of biotin at K248. (The spectrum shows the extended spectrum of Figure 4B and was included for comparison with the peptide fragment 6).

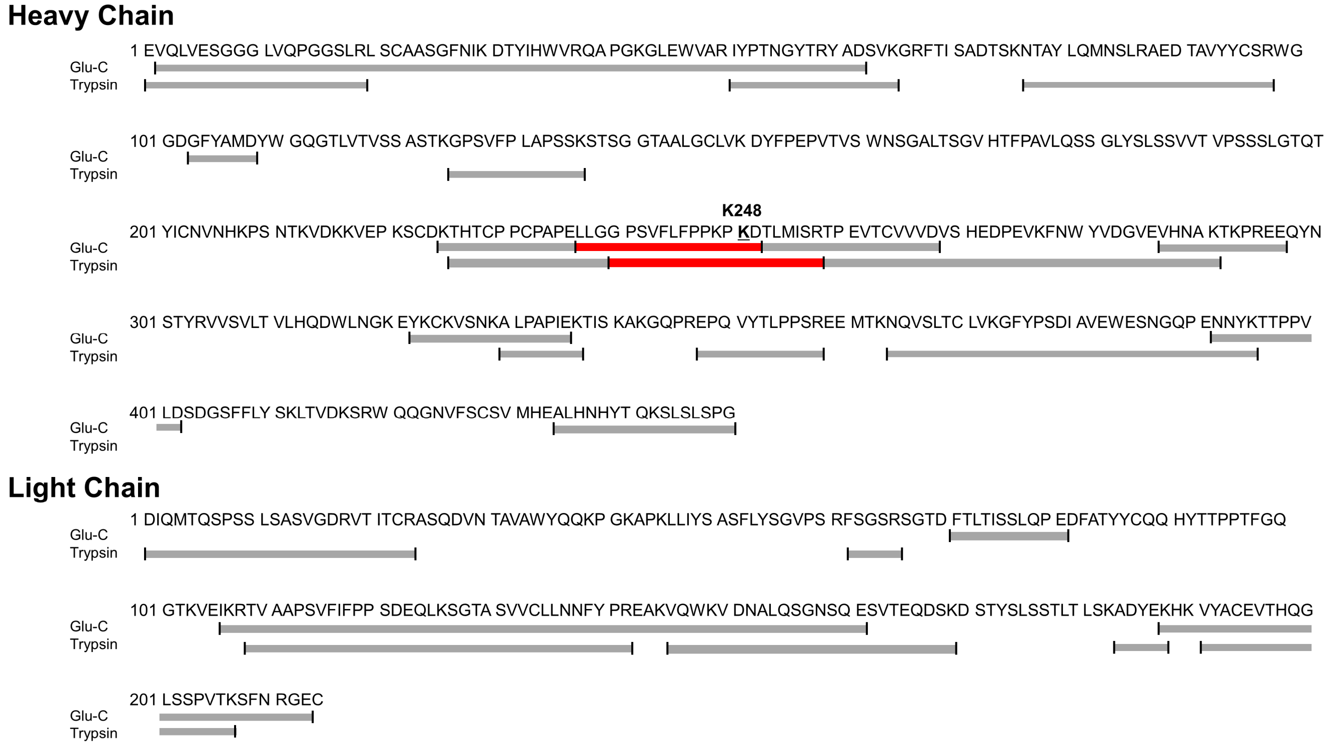

Figure S23. Protease digestion analysis of trastuzumab WT and Bt-conjugates using Glu-C and trypsin. Gray lines indicate the detected fragments. Red lines indicate the detected labeled-fragments.

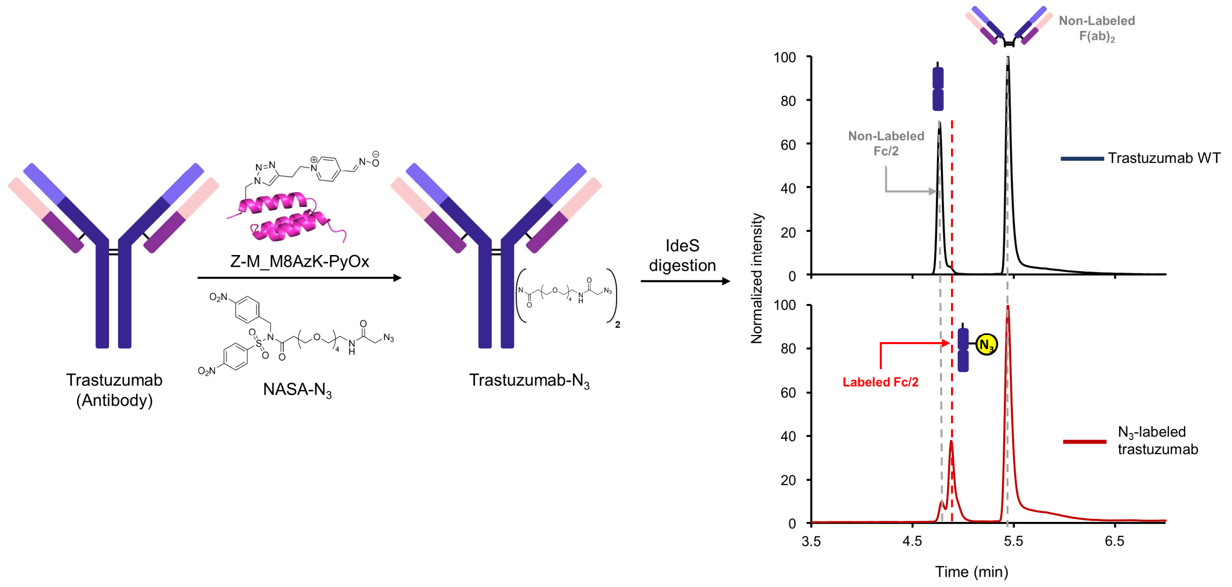

Figure S24. Analysis of trastuzumab WT and N_3-conjugates using HPLC. Trastuzumab was efficiently labeled with N_3, although the efficiency was slightly lower than NASA-Bt. Reaction conditions: trastuzumab (5 µM), Z-M-PyOx (60 µM), NASA-N_3 (60 µM), 50 mM HEPES (pH 8.0), 37 °C, and 6 hours.

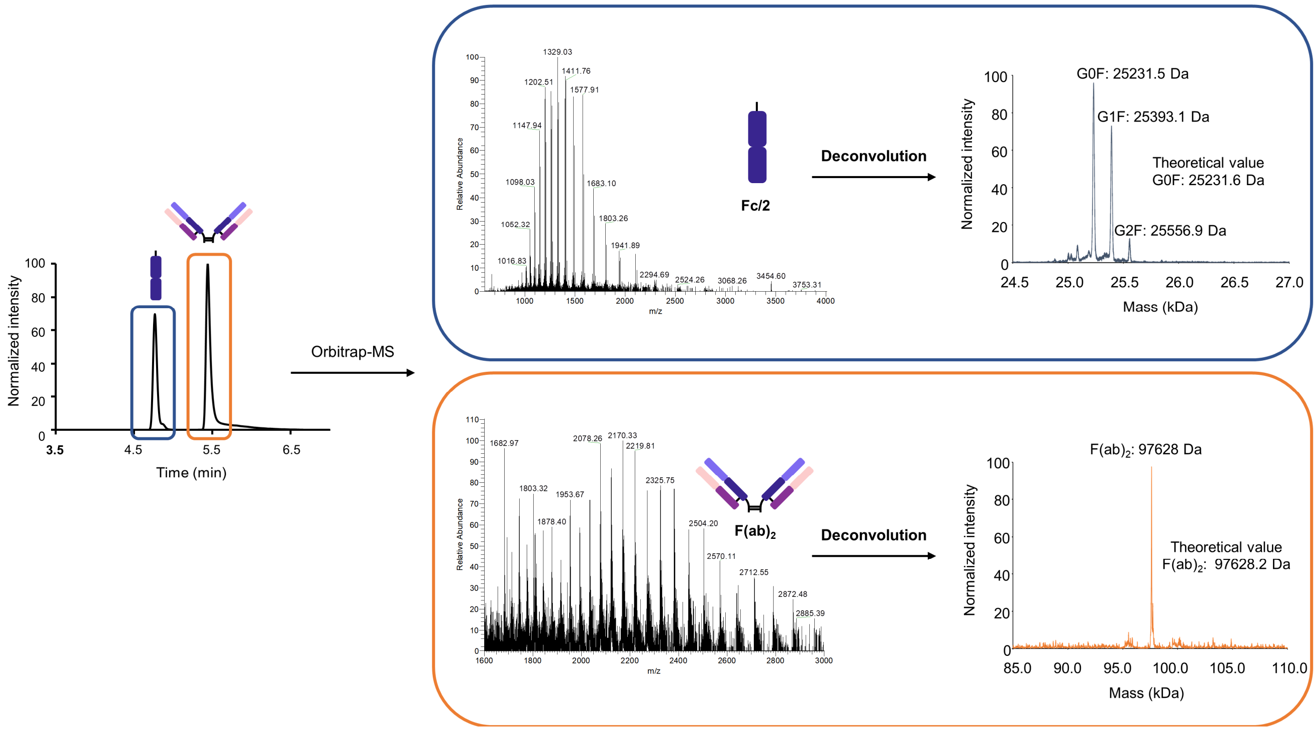

Figure S25. Orbitrap-MS analysis of IdeS-digested trastuzumab-WT. Fc/2 fragment exhibited several peaks corresponding to different glycan patterns.

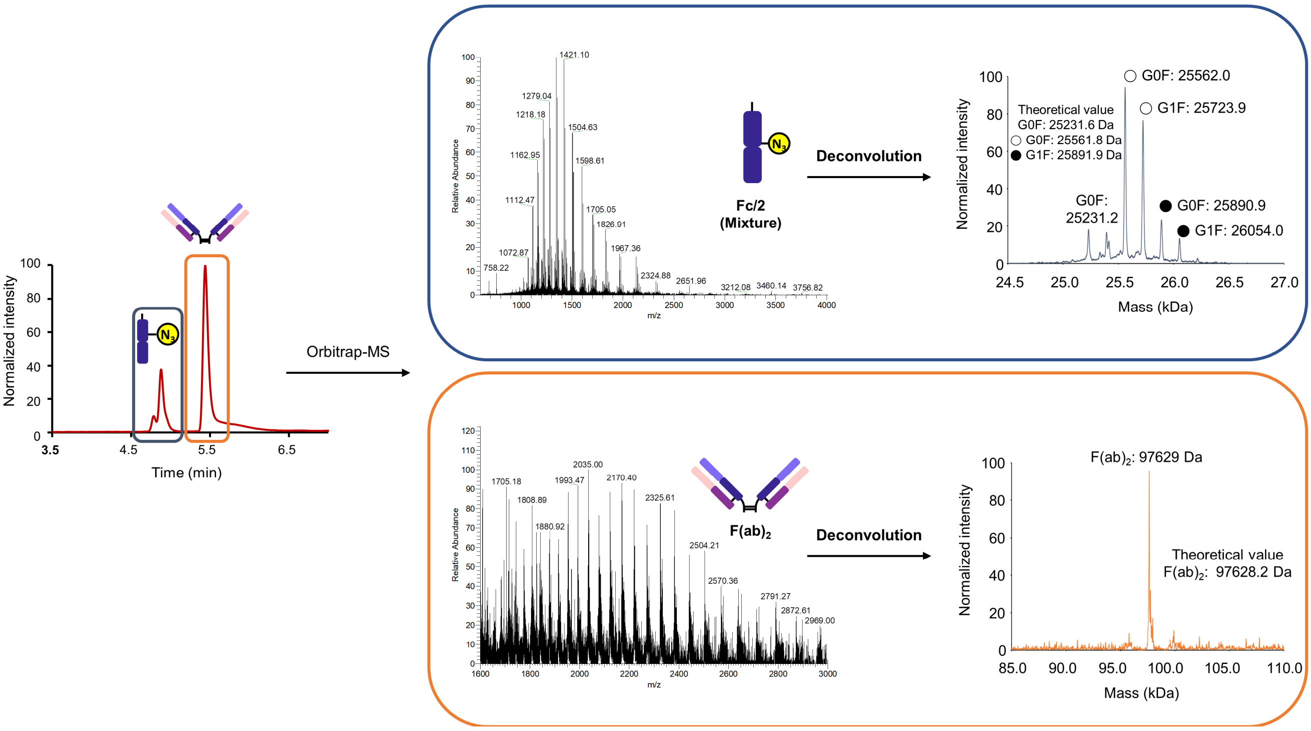

Figure S26. Orbitrap-MS analysis of IdeS-digested trastuzumab-N_3. Fc/2 fragment exhibited several peaks corresponding to different glycan patterns and the mixture of N_3-labeled species. The additional labeling could be attributed to K246-labeling. ○: one-N_3 labeled, ●: two-N_3 labeled (additional labeling)

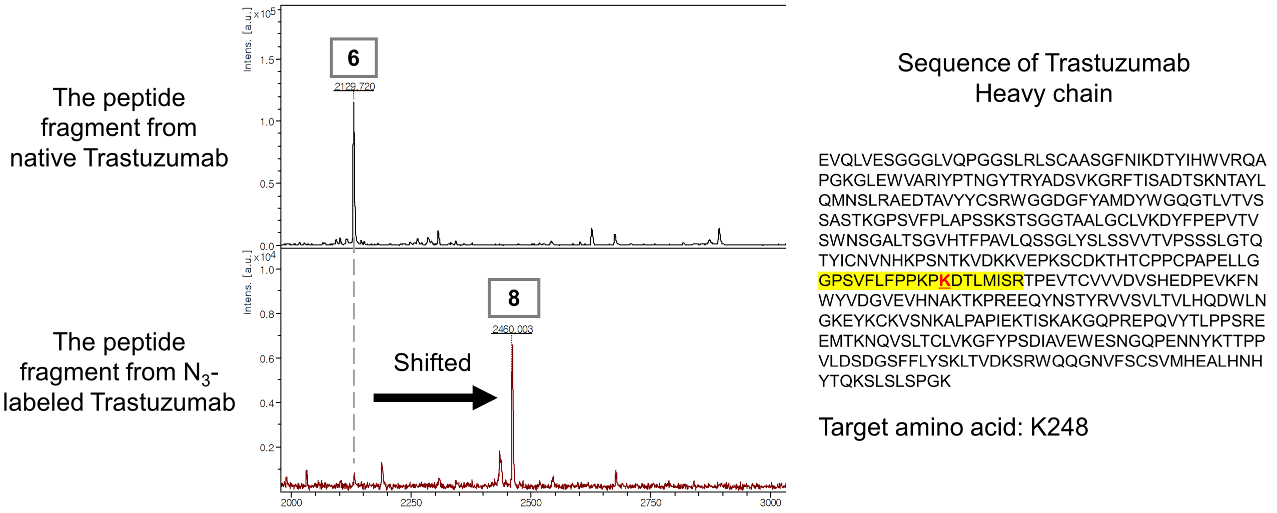

Figure S27. Protease (Trypsin) digestion analysis of WT, N_3-labeled trastuzumab. The peptide fragment 6 contains the expected target lysine (K248) and was shifted to show the mass of N_3-conjugated form (peptide fragment 8).

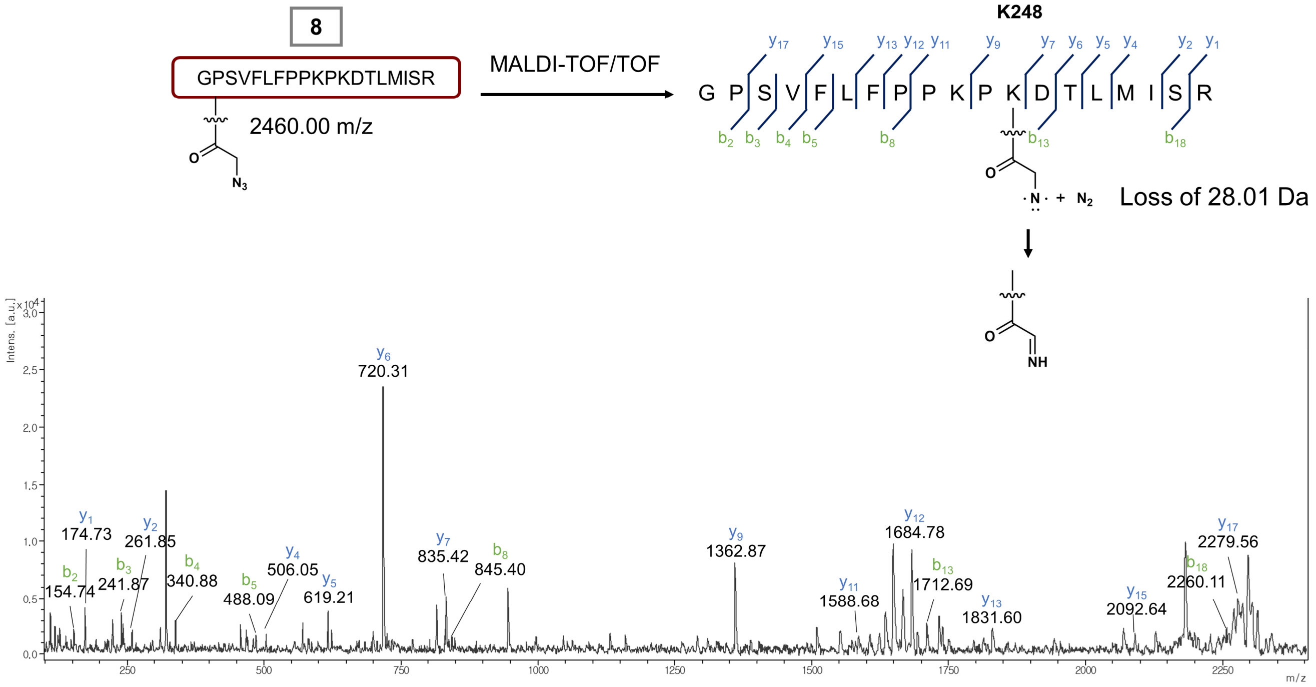

Figure S28. MALDI-TOF/TOF tandem MS analysis of peptide fragments 8, confirming the labeling of azide at K248. Particularly, the fragments exhibited the cleavage of azide to form an imine bond and a loss of 28.01 Da, attributed to the high-energy conditions during tandem MS analysis.[8]

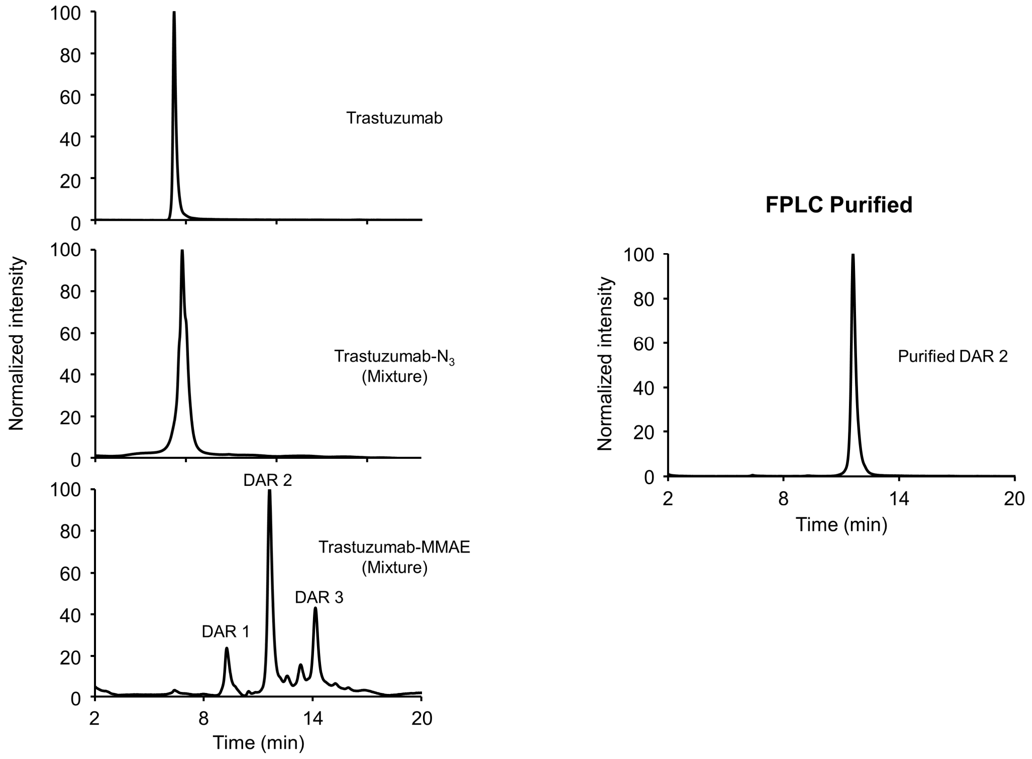

Figure S29. HIC analysis of trastuzumab WT, N3-conjugates, and MMAE-conjugates (mixture and purified). Although N_3 labeling and subsequent BCN-VC-PABC-MMAE conjugation resulted mixture of ADCs, FPLC purification successfully isolated DAR 2 species.

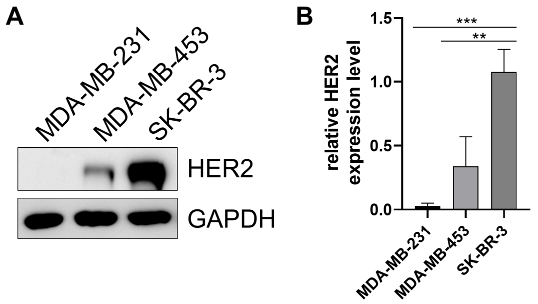

Figure S30. (A) Western blot of HER2 expression in MDA-MB-231, MDA-MB-453, and SK-BR-3 cells (n = 3). (B) Quantification result of the HER2 western blots relative to those of GAPDH. Bar graphs represent the average and the error bars represent the average ± standard deviation. Statistical analysis was conducted by one-way ANOVA analysis and Tukey's multiple comparison test (n = 3, ** p < 0.01, *** p < 0.001).

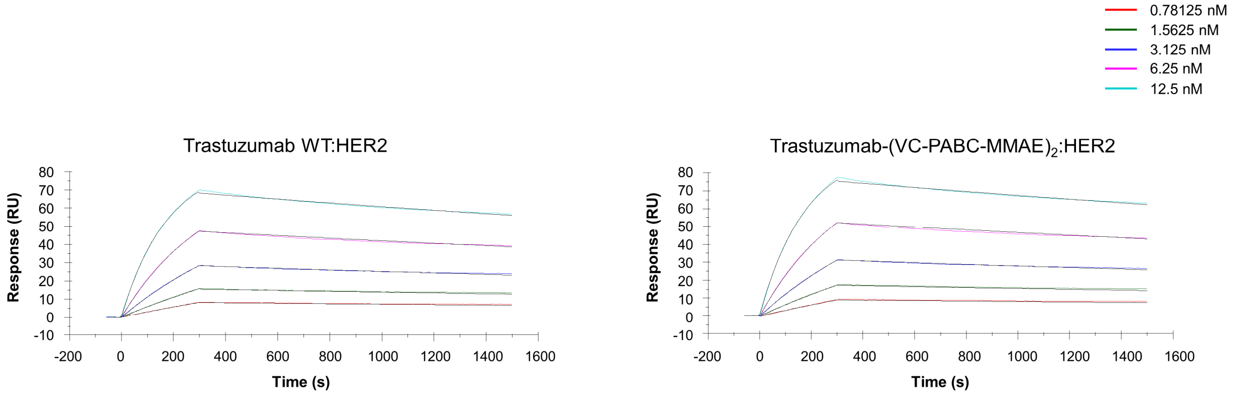

Figure S31. Binding assay of trastuzumab WT and the ADC product against HER2 using SPR.

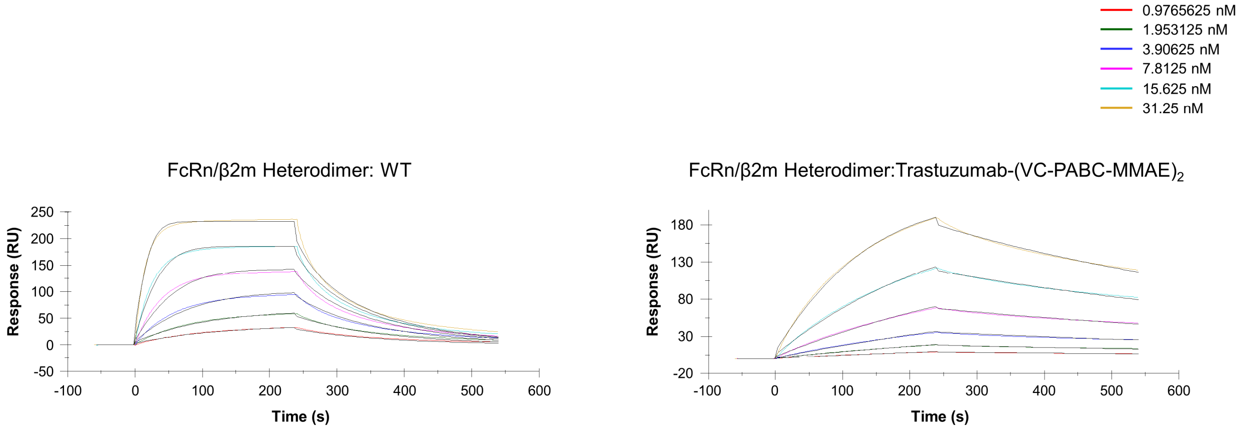

Figure S32. Binding assay of trastuzumab WT and the ADC product against FcRn/β2m Heterodimer at pH 6.0 using SPR.

4.2. Supplementary tables

Table S1. Binding constant of each protein against trastuzumab WT.

Sample	K_D (M)	k_a (1/Ms)	k_d (1/s)	R_{max}(RU)	Chi² (RU²)	U-value
**Z-M_WT**	4.964×10^{-9}	1.897×10^{5}	9.415×10^{-4}	66.66	0.832	2
**Z-M_M8AzK**	3.939×10^{-9}	1.820×10^{5}	7.169×10^{-4}	116.2	1.76	2
**Z-M_H19AzK**	5.101×10^{-9}	1.882×10^{5}	9.597×10^{-4}	62.52497	0.994	2
**Z-M_K33AzK**	4.846×10^{-9}	1.973×10^{5}	9.561×10^{-4}	70.48	0.948	2
**Z-M_D37AzK**	3.521×10^{-9}	1.941×10^{5}	6.835×10^{-4}	117.1	1.84	2
**Z-M_M8AzK-PyOx**	5.016×10^{-9}	1.914×10^{5}	9.602×10^{-4}	80.2403	0.963	2
**Z-M_H19AzK-PyOx**	4.944×10^{-9}	1.934×10^{5}	9.560×10^{-4}	72.21	0.841	1
**Z-M_K33AzK-PyOx**	3.481×10^{-9}	1.966×10^{5}	6.843×10^{-4}	118.3	2	2
**Z-M_D37AzK-PyOx**	4.792×10^{-9}	2.002×10^{5}	9.593×10^{-4}	70.51	0.961	2

Table S2. *In vitro* anti-tumor activities and cytotoxicity of the samples against MDA-MB-231, MDA-MB-453, and SK-BR-3.

Sample	GI_{50}s (nM)		
	**MDA-MB-231**	**MDA-MB-453**	**SK-BR-3**
**Trastuzumab WT**	>10	>10	>10
**Kadcyla**	>10	>10	0.249 ± 0.043
**Trastuzumab-MMAE$_2$**	>10	>10	0.125 ± 0.065

Table S3. Binding constant of trastuzumab WT and the ADC product against HER2 using SPR.

Sample	K_D (M)	k_a (1/Ms)	k_d (1/s)	R_{max}(RU)	Chi² (RU²)	U-value
**Trastuzumab-WT**	3.672×10^{-10}	4.343×10^{5}	1.595×10^{-4}	80.2403	0.256	1
**Trastuzumab-MMAE$_2$**	3.824×10^{-10}	4.340×10^{5}	1.660×10^{-4}	86.64	0.155	1

Table S4. Binding constant of trastuzumab WT and the ADC product against FcRn/β2m Heterodimer at pH 6.0 using SPR.

Sample	K_D (M)	k_a (1/Ms)	k_d (1/s)	R_{max}(RU)	Chi² (RU²)	U-value
**Trastuzumab-WT**	5.718×10^{-9}	3.123×10^{6}	1.786×10^{-2}	231.7	14.1	2
**Trastuzumab-MMAE$_2$**	6.040×10^{-9}	2.950×10^{5}	1.782×10^{-3}	246.6	1.42	1

Table S5. Sequences of the proteins used in this study. Residues used for ncAA incorporation are bolded and underlined.

Protein	Sequence
**Z-DM**	MGWSHPQFEKGSVDNKFNKEQQNAFYEILHLPNLNEEQRNAFIQSLKDDPSQSANLLAEAKKLNDAQAPKGSGHHHHHH
**Z-AFB**	MVDN**K**FNKELSVAGREIVTLPNLNDPQK**K**AFI**F**SLWDDPSQSANLLAEAKKLNDAQAPKGSHHHHHH
**TrpR**	MAQQSPYSAAMAEQRHQEWLRFVDLLKNAYQNDLHLPLLNLMLTPDEREALGTRVRIVEELLRGEMSQRELKNELGAGIATITRGSNSLKAAPVEL RQWLEEVLLKSDGGHHHHHH
**Lysozyme**	KVFGRCELAAAMKRHGLDNYRGYSLGNWVCAAKFESNFNTQATNRNTDGSTDYGILQINSRWWCNDGRTPGSRNLCNIPCSALLSSDITASVNC AKKIVSDGNGMNAWVAWRNRCKGTDVQAWIRGCRL
**Z-M protein**	MVDNKFN**M**QQQRRFYEAL**H**DPNLNEEQRNAKI**K**SIR**D**DPSQSANLLAEAKKLNDAQAPKGSHHHHHH
**Fc Fragment**	ASDKTHTCPPCPAPELLGGPSVFLFPPKPKDTLMISRTPEVTCVVVDVSHEDPEVKFNWYVDGVEVHNAKTKPREEQYNSTYRVVSVLTVLHQD WLNGKEYKCKVSNKALPAPIEKTISKAKGQPREPQVYTLPPSREEMTKNQVSLTCLVKGFYPSDIAVEWESNGQPENNYKTTPPVLDSDGSFFLYS KLTVDKSRWQQGNVFSCSVMHEALHNHYTQKSLSLSPG
**Herceptin**	(Heavy Chain)<br>EVQLVESGGGLVQPGGSLRLSCAASGFNIKDTYIHWVRQAPGKGLEWVARIYPTNGYTRYADSVKGRFTISADTSKNTAYLQMNSLRAEDTAVYY CSRWGGDGFYAMDYWGQGTLVTVSSASTKGPSVFPLAPSSKSTSGGTAALGCLVKDYFPEPVTVSWNSGALTSGVHTFPAVLQSSGLYSLSSVV TVPSSSLGTQTYICNVNHKPSNTKVDKKVEPKSCDKTHTCPPCPAPELLGGPSVFLFPPKPKDTLMISRTPEVTCVVVDVSHEDPEVKFNWYVDG VEVHNAKTKPREEQYNSTYRVVSVLTVLHQDWLNGKEYKCKVSNKALPAPIEKTISKAKGQPREPQVYTLPPSREEMTKNQVSLTCLVKGFYPSDI AVEWESNGQPENNYKTTPPVLDSDGSFFLYSKLTVDKSRWQQGNVFSCSVMHEALHNHYTQKSLSLSPG<br>(Light Chain)<br>DIQMTQSPSSLSASVGDRVTITCRASQDVNTAVAWYQQKPGKAPKLLIYSASFLYSGVPSRFSGSRSGTDFTLTISSLQPEDFATYYCQQHYTTPPT FGQGTKVEIKRTVAAPSVFIFPPSDEQLKSGTASVVCLLNNFYPREAKVQWKVDNALQSGNSQESVTEQDSKDSTYSLSSTLTLSKADYEKHKVYA CEVTHQGLSSPVTKSFNRGEC

5. Spectral data

NMR spectrum of ***PyOx-Butyne***

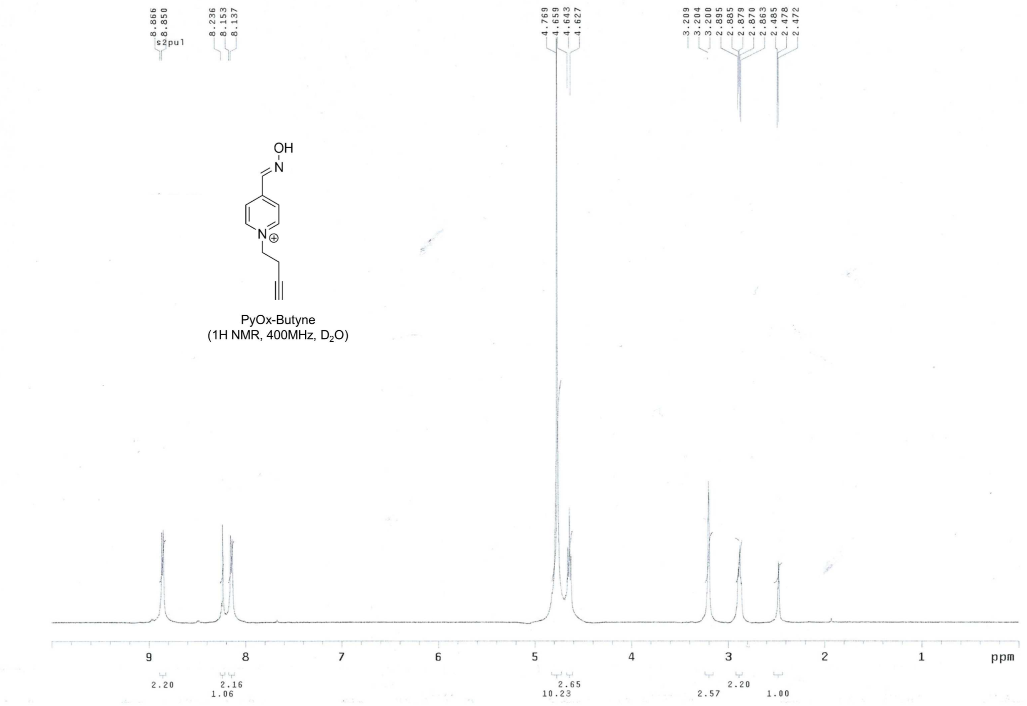

NMR spectrum of ***PyOx-Hexyne***

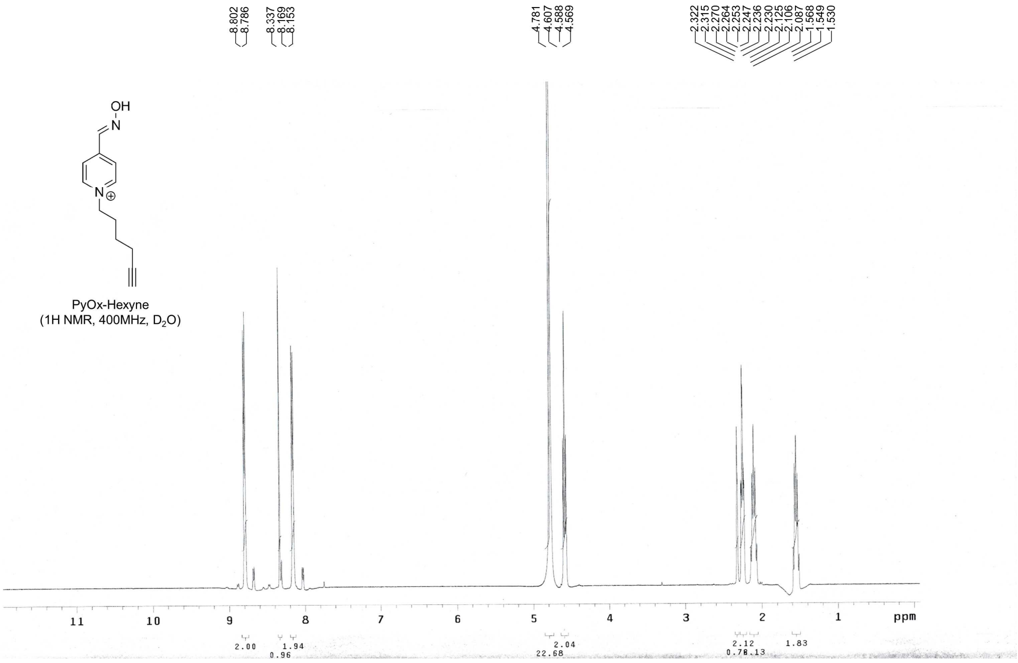

*NMR spectrum of **NASA-Bt***

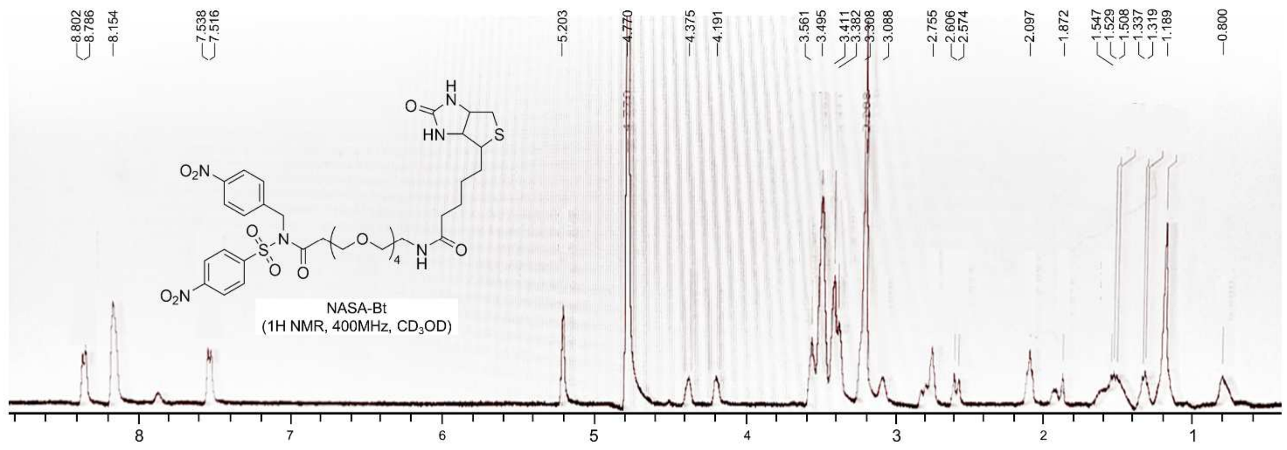

*NMR spectrum of **NASA-FL***

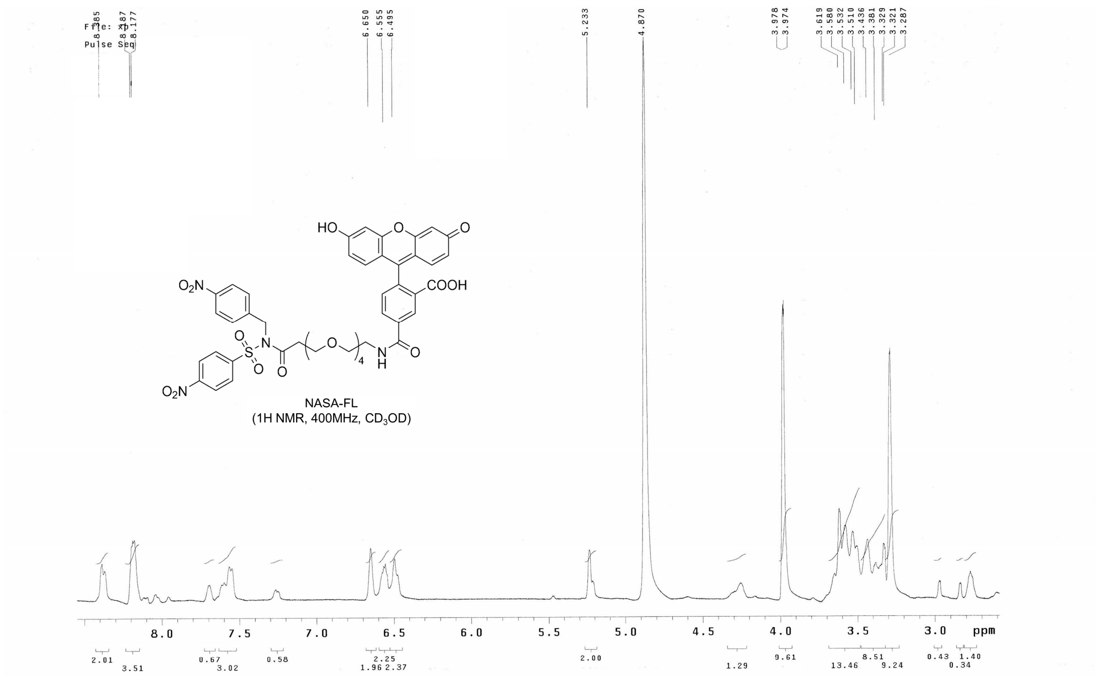

NMR spectrum of ***S1***

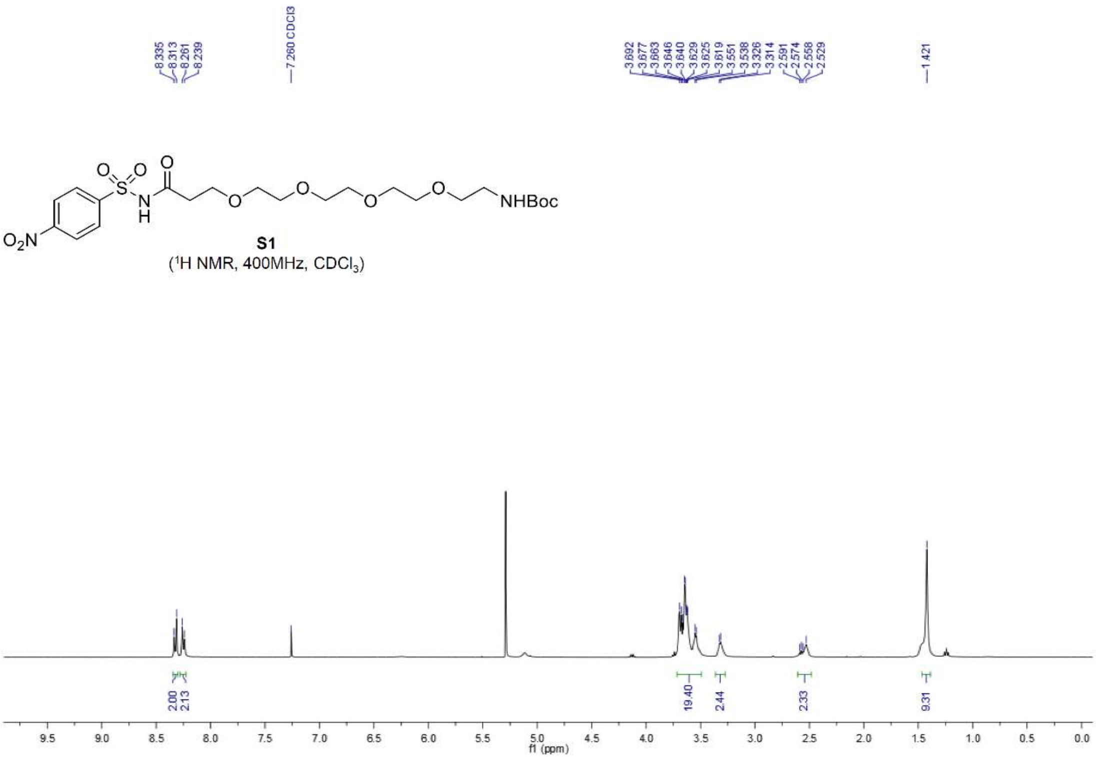

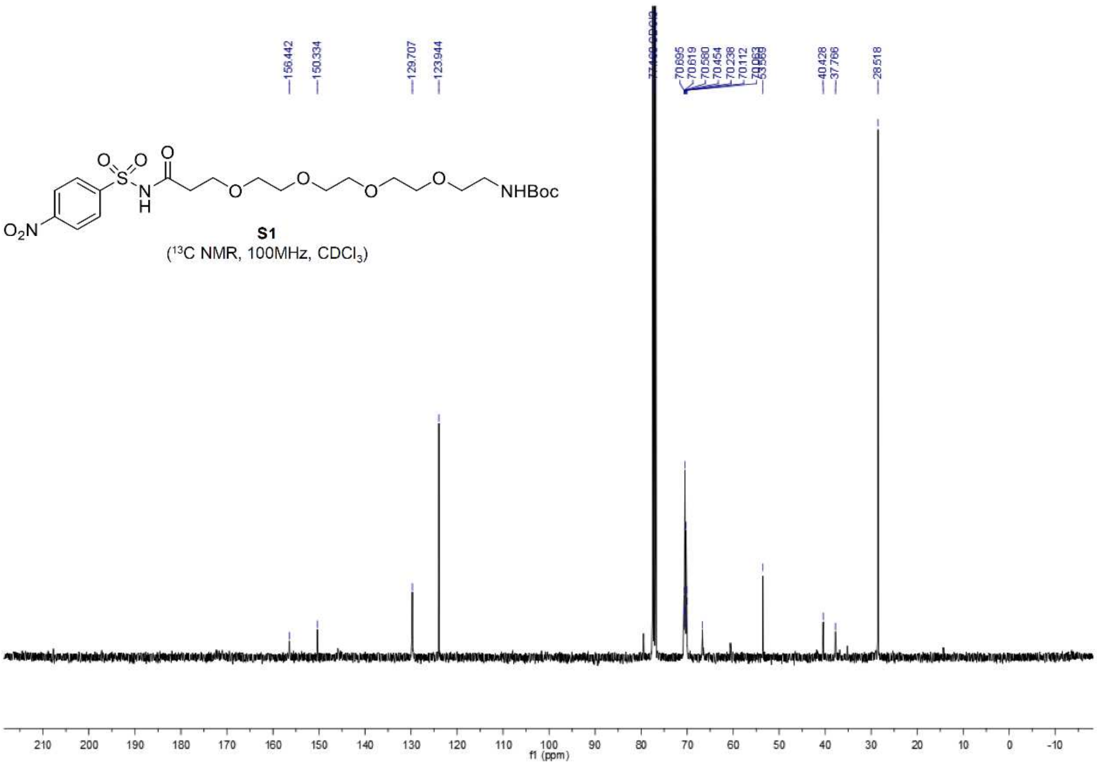

*NMR spectrum of **S2***

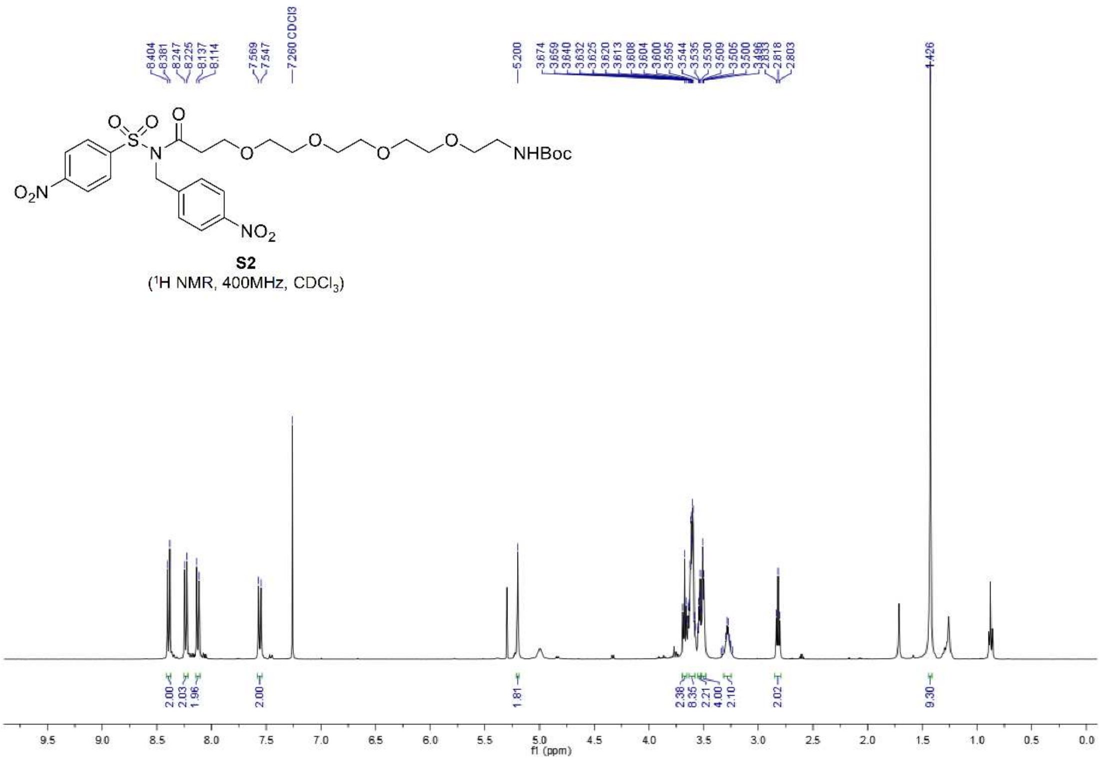

S2
(^{1}H NMR, 400MHz, $CDCl_3$)

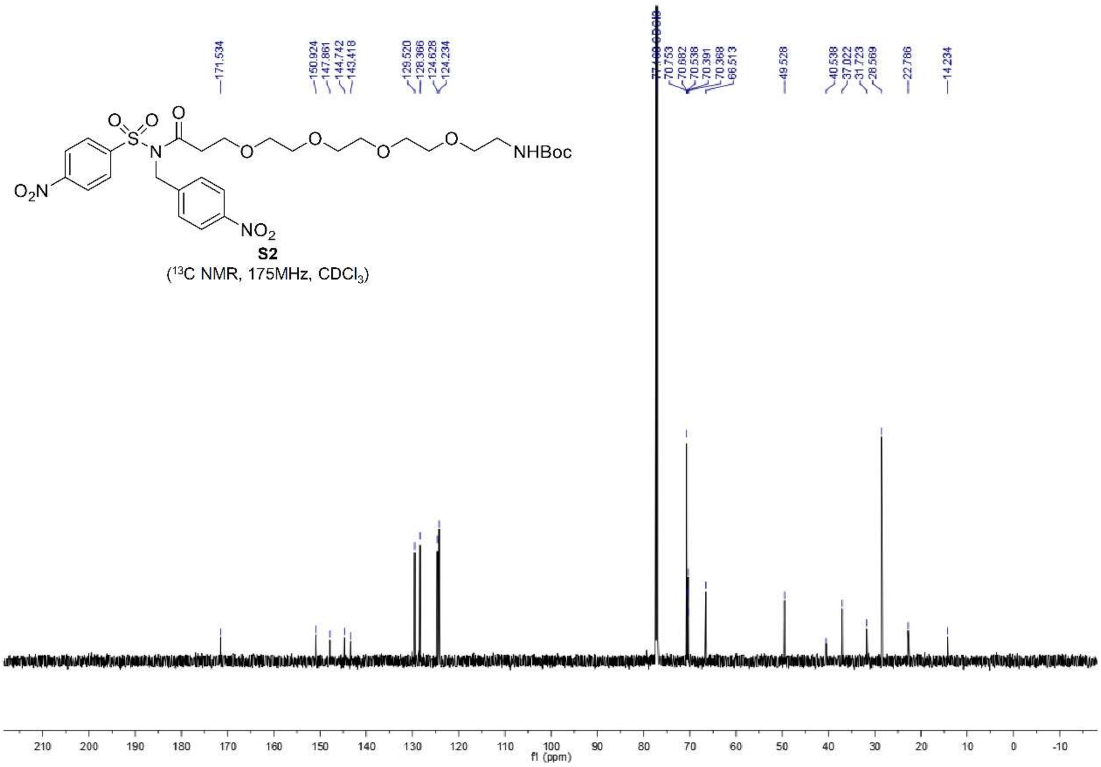

S2
(^{13}C NMR, 175MHz, $CDCl_3$)

*NMR spectrum of **NASA-N3***

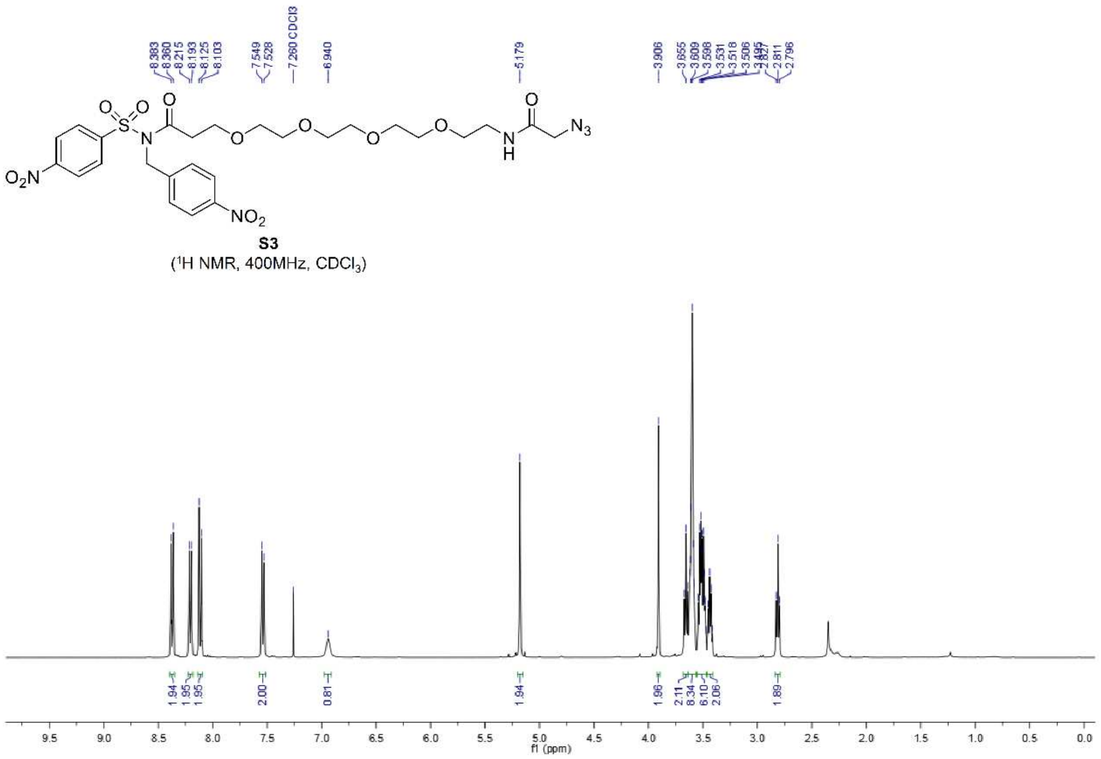

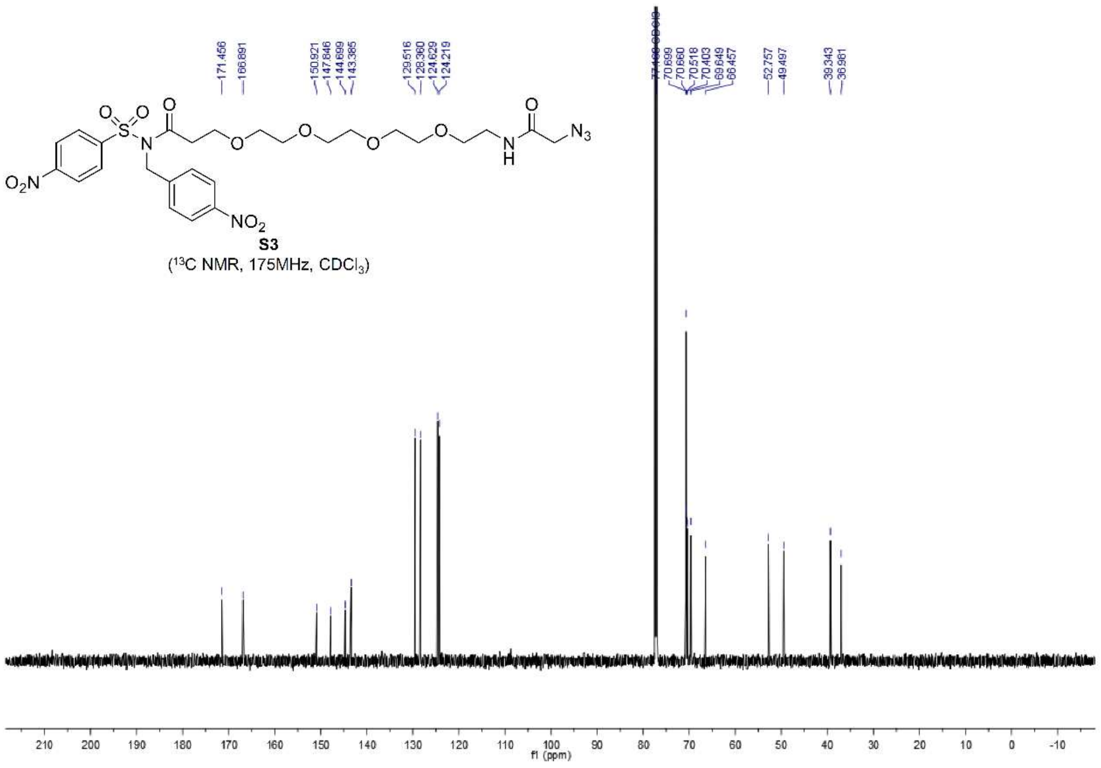

Chromatogram and MS spectrum of **Fmoc-VC-PABC-MMAE**

<Chromatogram>

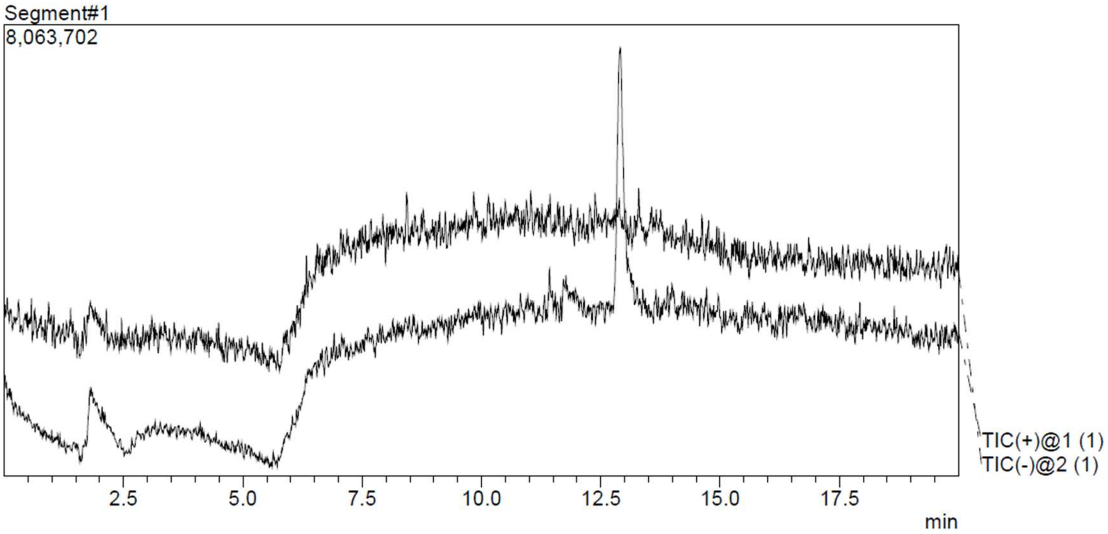

<Spectrum>

R.Time:----(Scan#:----)
MassPeaks:1951 BasePeak:83(1613495)
Spectrum Mode:Averaged 12.667-13.180(3801-3955)
BG Mode:None Polarity:Positive Segment 1 - Event 1

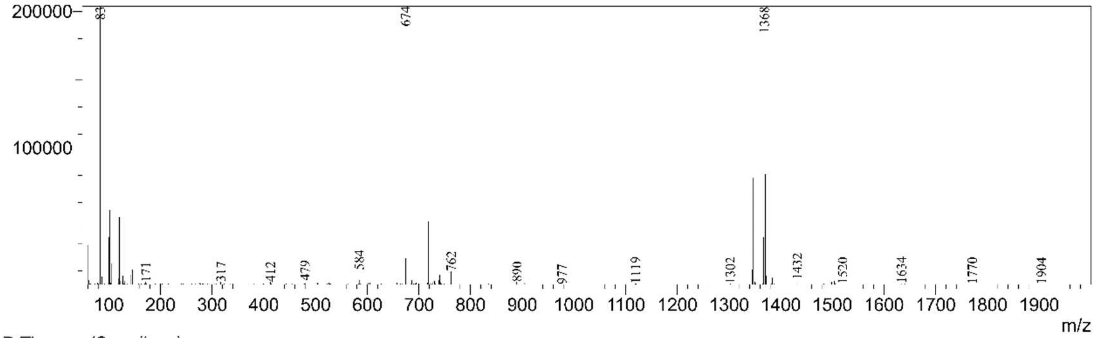

Chromatogram and MS spectrum of **NH_2-VC-PABC-MMAE**

<Chromatogram>

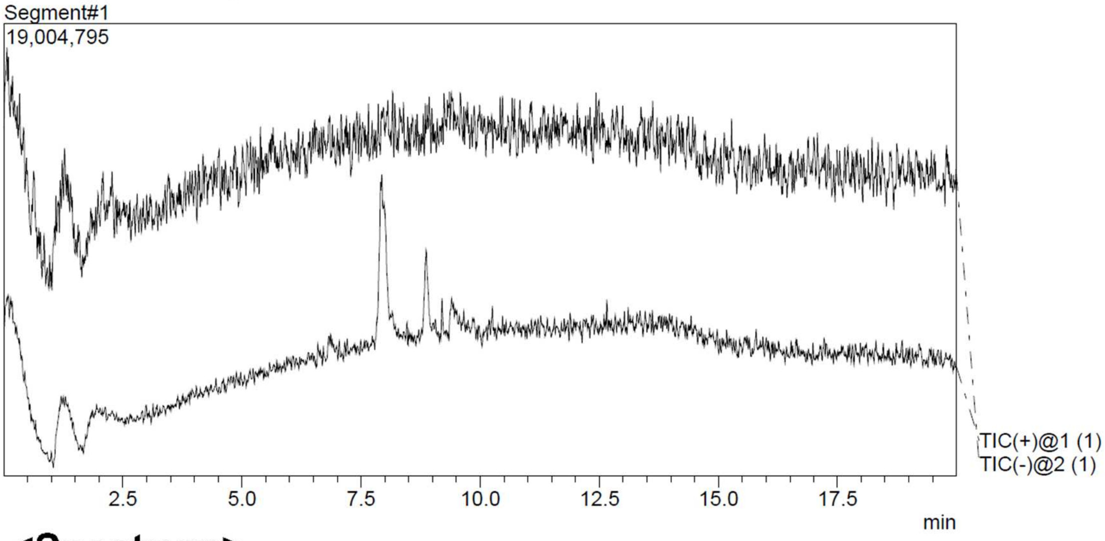

<Spectrum>

R.Time:----(Scan#:----)
MassPeaks:1951 BasePeak:83(2671881)
Spectrum Mode:Averaged 7.820-8.180(2347-2455)
BG Mode:None Polarity:Positive Segment 1 - Event 1

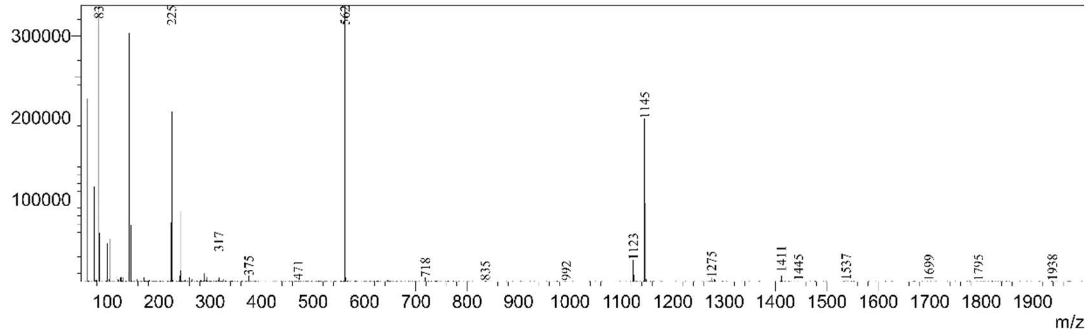

Chromatogram and MS spectrum of ***BCN-VC-PABC-MMAE***

<Chromatogram>

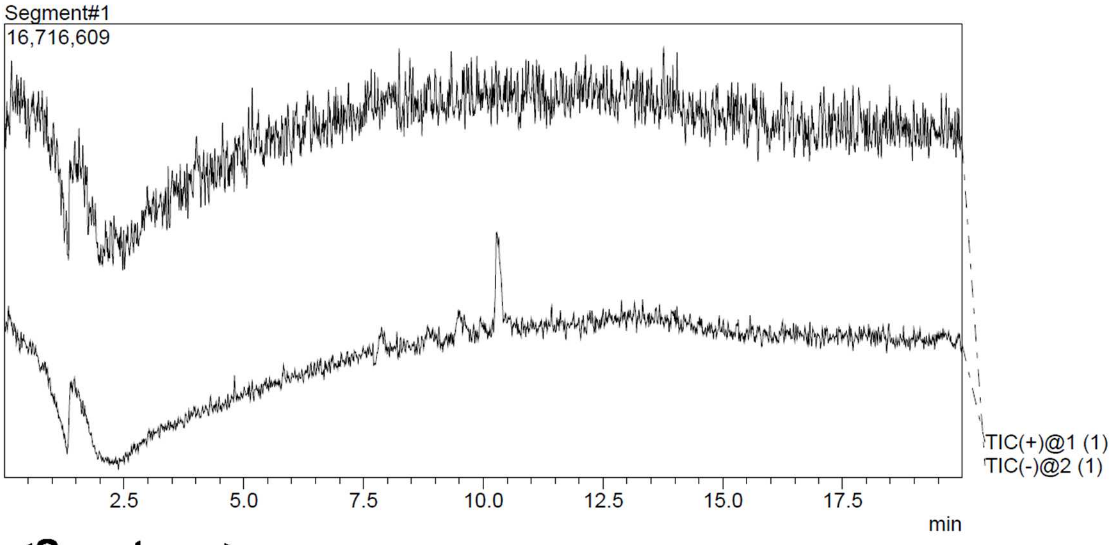

<Spectrum>

R.Time:----(Scan#:----)
MassPeaks:1951 BasePeak:101(2701895)
Spectrum Mode:Averaged 10.093-10.453(3029-3137)
BG Mode:None Polarity:Positive Segment 1 - Event 1

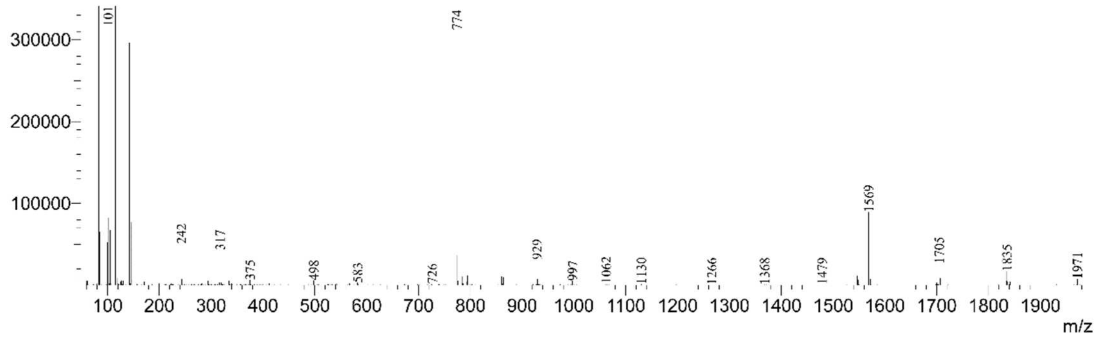

HRMS spectrum of compounds

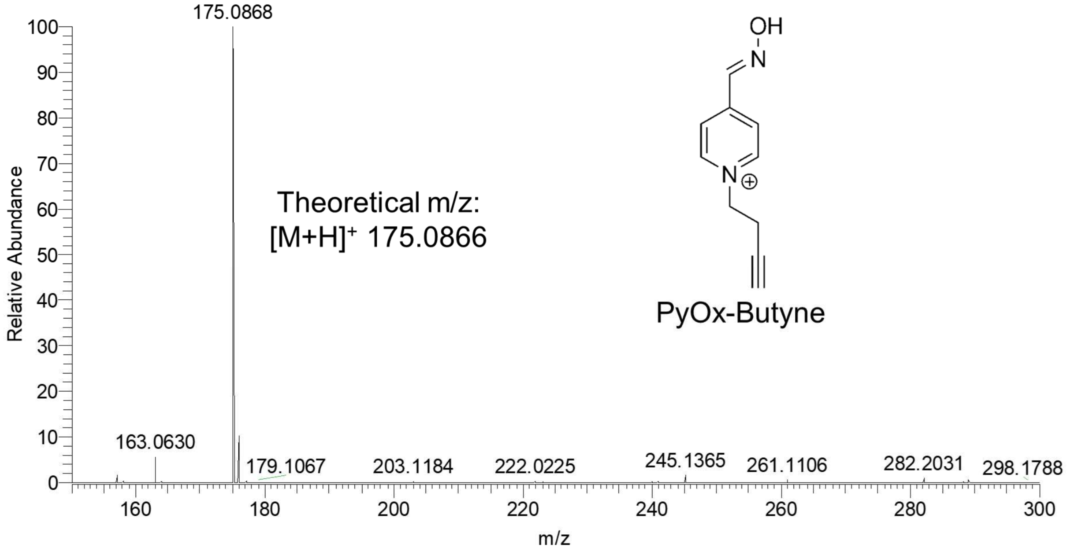

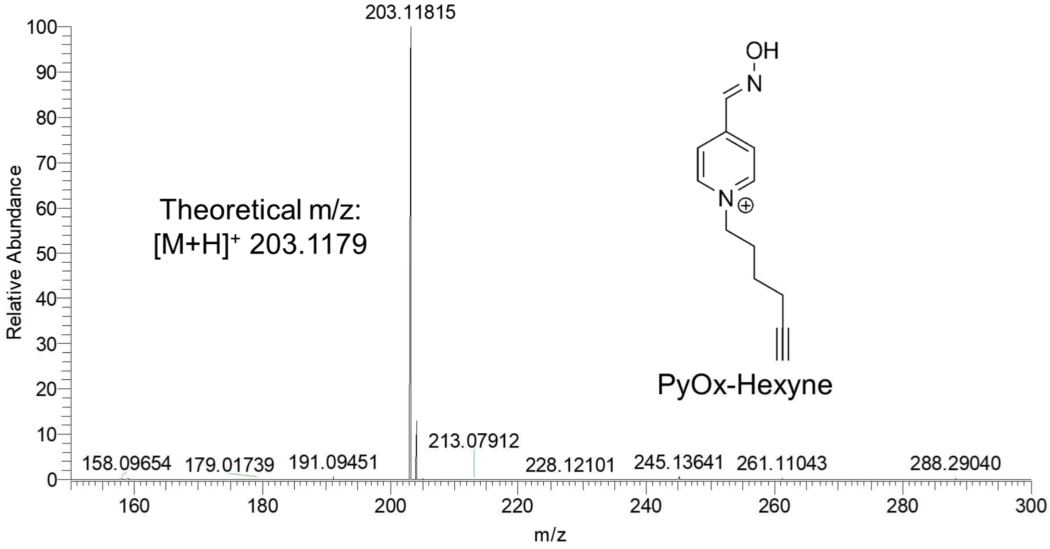

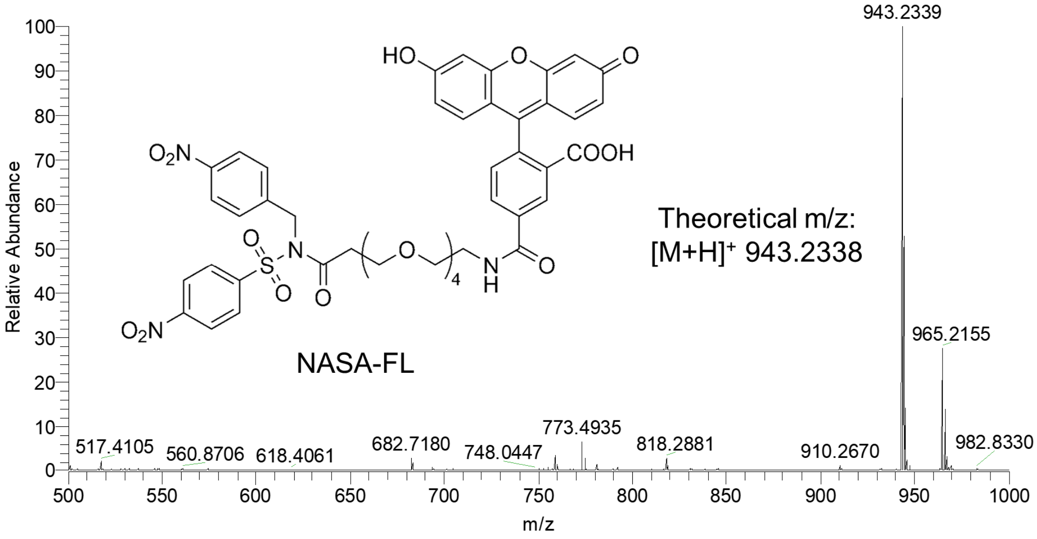

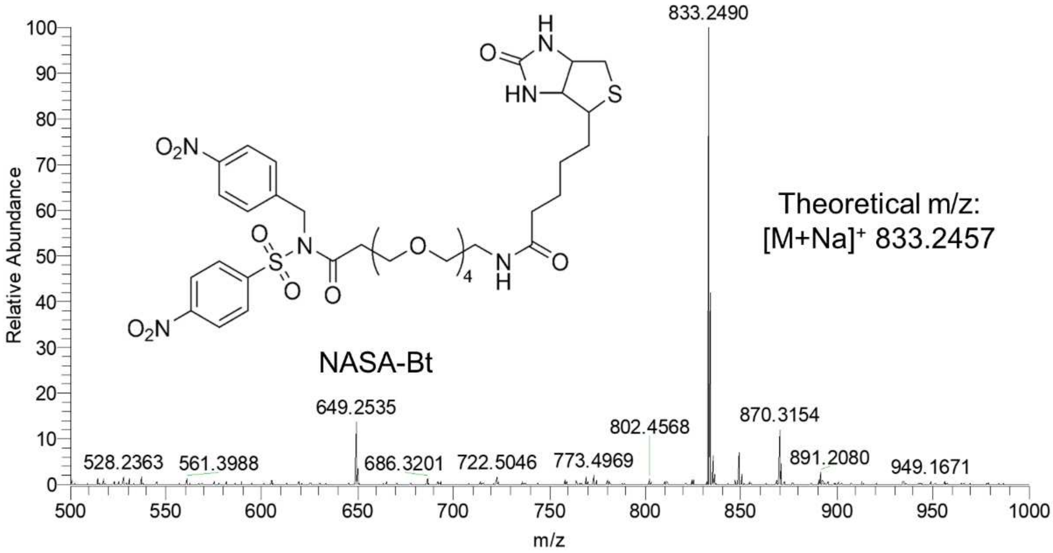
833.2490
Relative Abundance
NASA-Bt
Theoretical m/z:
[M+Na]+ 833.2457
649.2535
802.4568
870.3154
528.2363
561.3988
686.3201
722.5046
773.4969
891.2080
949.1671
m/z

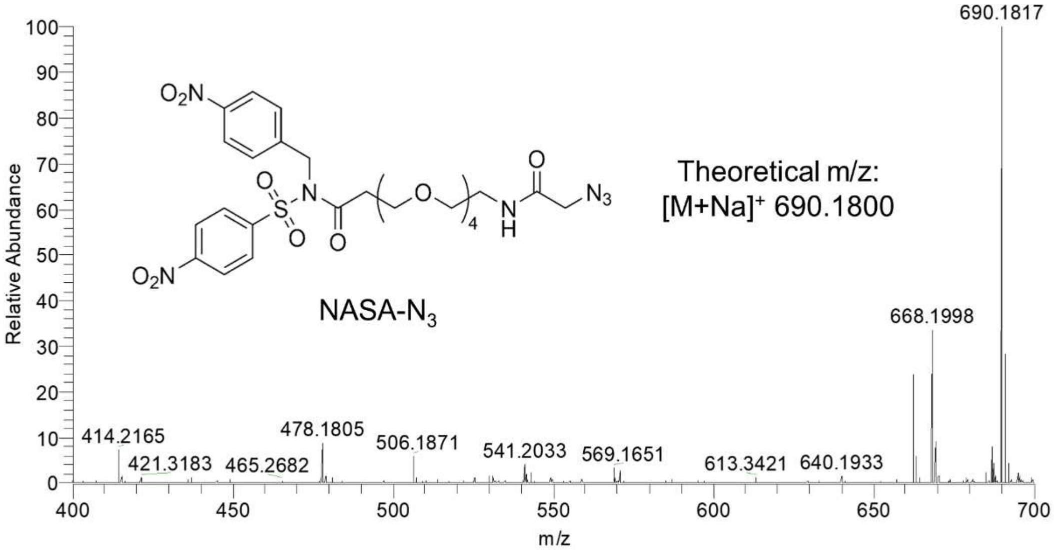
690.1817
Relative Abundance
NASA-N3
Theoretical m/z:
[M+Na]+ 690.1800
668.1998
414.2165
421.3183
465.2682
478.1805
506.1871
541.2033
569.1651
613.3421
640.1933
m/z

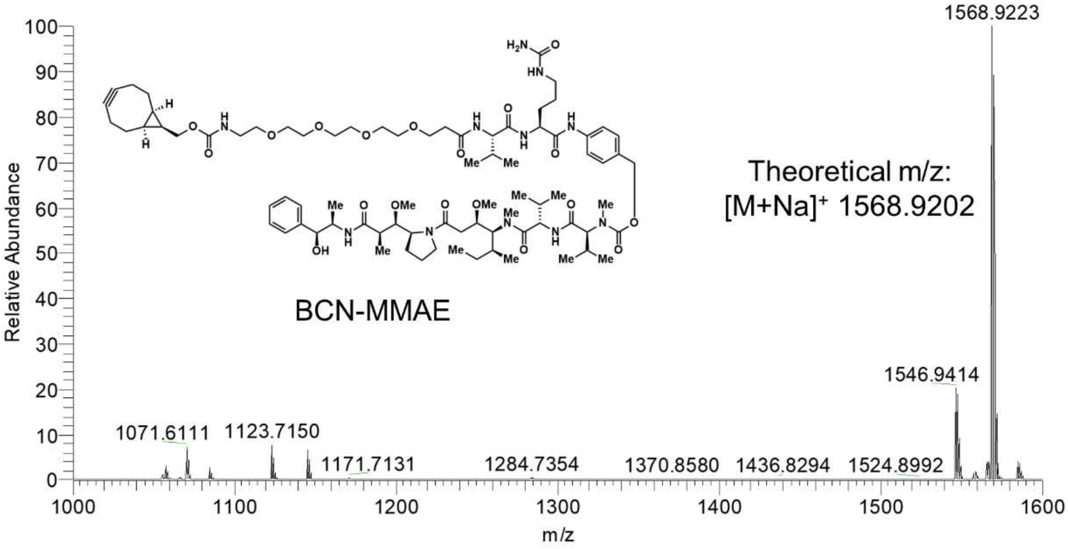
1568.9223
Relative Abundance
BCN-MMAE
Theoretical m/z:
[M+Na]+ 1568.9202
1546.9414
1071.6111
1123.7150
1171.7131
1284.7354
1370.8580
1436.8294
1524.8992
m/z

6. References

[1] M. T. Marty, A. J. Baldwin, E. G. Marklund, G. K. A. Hochberg, J. L. P. Benesch, C. V. Robinson, *Anal. Chem.* **2015**, *87*, 4370–4376.

[2] F. W. Studier, *Protein Expr Purif* **2005**, *41*, 207–234.

[3] A. C. Braisted, J. A. Wells, *Proc. Natl. Acad. Sci. U.S.A.* **1996**, *93*, 5688–5692.

[4] J. W. Chin, S. W. Santoro, A. B. Martin, D. S. King, L. Wang, P. G. Schultz, *J. Am. Chem. Soc.* **2002**, *124*, 9026–9027.

[5] D. P. Nguyen, H. Lusic, H. Neumann, P. B. Kapadnis, A. Deiters, J. W. Chin, *J. Am. Chem. Soc.* **2009**, *131*, 8720–8721.

[6] M. D. Hanwell, D. E. Curtis, D. C. Lonie, T. Vandermeersch, E. Zurek, G. R. Hutchison, *J. Cheminform.* **2012**, *4*, 17.

[7] J. Jumper, R. Evans, A. Pritzel, T. Green, M. Figurnov, O. Ronneberger, K. Tunyasuvunakool, R. Bates, A. Žídek, A. Potapenko, A. Bridgland, C. Meyer, S. A. A. Kohl, A. J. Ballard, A. Cowie, B. Romera-Paredes, S. Nikolov, R. Jain, J. Adler, T. Back, S. Petersen, D. Reiman, E. Clancy, M. Zielinski, M. Steinegger, M. Pacholska, T. Berghammer, S. Bodenstein, D. Silver, O. Vinyals, A. W. Senior, K. Kavukcuoglu, P. Kohli, D. Hassabis, *Nature* **2021**, *596*, 583–589.

[8] a) J. M. Dyke, A. P. Groves, A. Morris, J. S. Ogden, M. I. Catarino, A. A. Dias, A. M. S. Oliveira, M. L. Costa, M. T. Barros, M. H. Cabral, A. M. C. Moutinho, *Phys. Chem. A* **1999**, *103,* 8239–8245; b) H. Fazlıoğlu, J. Hacaloğlu, *J. Anal. Appl. Pyrolysis* **2002**, *63*, 327–338
